# Supplementary material for: Inter-kingdom signaling by the Legionella autoinducer LAI-1 involves the antimicrobial guanylate binding protein GBP
Source: PLoS Pathog. 2025 Apr 29;21(4):e1013026. doi: 10.1371/journal.ppat.1013026 (PMC12040241; doi:10.1371/journal.ppat.1013026)
Supplement: S1 Fig — (A) The Vibrio cholerae α-hydroxyketone reporter strain MM920 was left untreated or treated with the indicated concentrations of synthetic (S)-LAI-1, azido-LAI-1, diazirine-LAI-1, or DMSO (solvent control), and luminescence intensity was measured by a plate reader (30°C, 10 h). RLU, relative light units. Data shown are biological triplicates of means and standard deviations. (B) D. discoideum Ax2 was treated with LAI-1 or clickable azido-LAI-1 (10 µM, 1 h), infected (MOI 5, 4 h) with mCerulean-producing L. pneumophila JR32 (pNP99), clicked with DIBO594 dye, and analyzed by confocal laser scanning microscopy. Scale bars: 20 μm. (C) D. discoideum Ax2 producing P4C-mCherry (pWS032) was treated (10 µM, 1 h) with LAI-1, azido-LAI-1, or DMSO (solvent control), infected (MOI 5, 4 h) with mCerulean-producing L. pneumophila JR32 (pNP99), fixed and analyzed by confocal microscopy. LCV areas were quantified using ImageJ software. Data shown are means and standard deviations of biological triplicates (Student’s t-test; *, p ≤ 0.05; **, p ≤ 0.01). (PDF) [file ppat.1013026.s001.pdf]

Figure S1

A

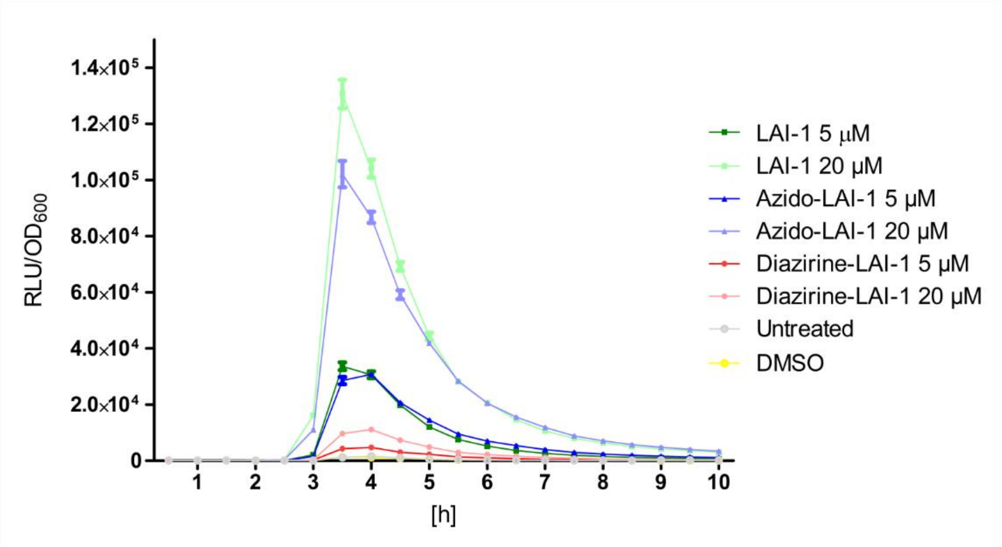

B

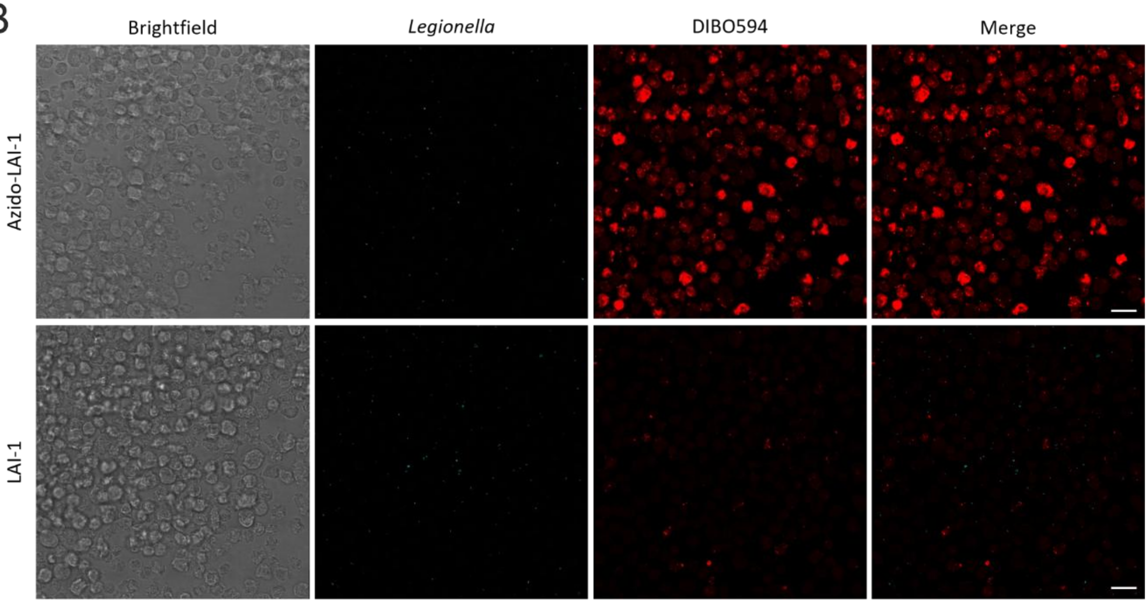

C

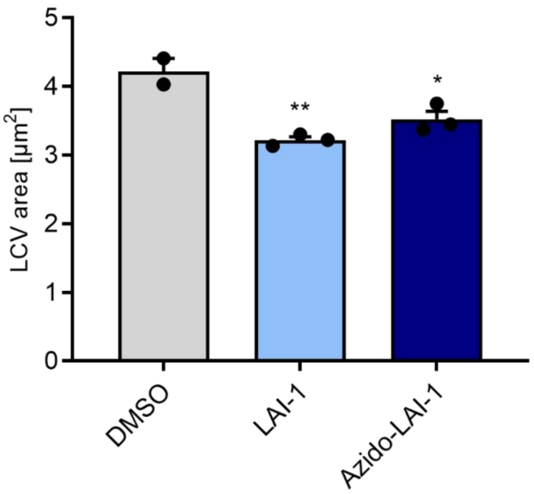

Fig. S1. LAI-1 and clickable derivatives promote luminescence of a *Vibrio* reporter strain and LCV size modulation. (**A**) The *Vibrio cholerae* α-hydroxyketone reporter strain MM920 was left untreated or treated with the indicated concentrations of synthetic (*S*)-LAI-1, azido-LAI-1, diazirine-LAI-1, or DMSO (solvent control), and luminescence intensity was measured by a plate reader (30°C, 10 h). RLU, relative light units. Data shown are biological triplicates of means and standard deviations. (**B**) *D. discoideum* Ax2 was treated with LAI-1 or clickable azido-LAI-1 (10 µM, 1 h), infected (MOI 5, 4 h) with mCerulean-producing *L. pneumophila* JR32 (pNP99), clicked with DIBO594 dye, and analyzed by confocal laser scanning microscopy. Scale bars: 20 µm. (**C**) *D. discoideum* Ax2 producing P4C-mCherry (pWS032) was treated (10 µM, 1 h) with LAI-1, azido-LAI-1, or DMSO (solvent control), infected (MOI 5, 4 h) with mCerulean-producing *L. pneumophila* JR32 (pNP99), fixed and analyzed by confocal microscopy. LCV areas were quantified using ImageJ software. Data shown are means and standard deviations of biological triplicates (Student's t-test; *, $p \leq 0.05$; **, $p \leq 0.01$).
