# Supplementary material for: Inter-kingdom signaling by the Legionella autoinducer LAI-1 involves the antimicrobial guanylate binding protein GBP
Source: PLoS Pathog. 2025 Apr 29;21(4):e1013026. doi: 10.1371/journal.ppat.1013026 (PMC12040241; doi:10.1371/journal.ppat.1013026)
Supplement: S2 Fig — (A) Sequence alignment of DdGBP and human GBP1 (hGBP1) was made using T-Coffee (www.ebi.ac.uk/ jdispatcher/msa/tcoffee) and analyzed with ESPript 3.0 (espript.ibcp.fr/ESPript/ESPript/) with published (hGBP1) or predicted (DdGBP) structural data (hGBP1 – UniProtKB: P32455, PDB: 1DG3; DdGBP – UniProtKB: Q54TN9). P-Loop, GTPase, and helical domains are displayed, as well as the predicted N-terminal signal peptide and C-terminal transmembrane domains of DdGBP, and the C-terminal CaaX prenylation motif of hGBP1. The α-helical domain is more extended in DdGBP than in hGBP1. (B) Structural model of DdGBP as predicted by AlphaFold3 (AlphaFoldDB: AF-Q54TN9-F1-v4): N-terminus (blue), C-terminus (red). (C) Proteinase K digestion (30 min) using intact or Triton X-100-treated crude membranes of D. discoideum producing the indicated GFP-tagged proteins. Immunoblot using anti-GFP and anti-protein disulfide isomerase (PDI) antibodies is shown (left: molecular weight markers (kDa), below: digestion (%) of GFP-tagged proteins). PDI was protected from proteinase K digestion due to its localization to the lumen of the ER, while GFP-tagged calnexin A (CnxA-GFP) was susceptible to proteinase K treatment, as the GFP tag is positioned towards the cytosol. Both N- and C-terminally GFP-tagged DdGBP was degraded by proteinase K, indicating that DdGBP is anchored in the ER membrane with its GTPase domain facing the cytosol. The data shown is representative of two independent biological replicates. (D) Presumed topology of GFP-tagged DdGBP and CnxA, taking into account the predicted C-terminal transmembrane domain of DdGBP. (PDF) [file ppat.1013026.s002.pdf]

Figure S2

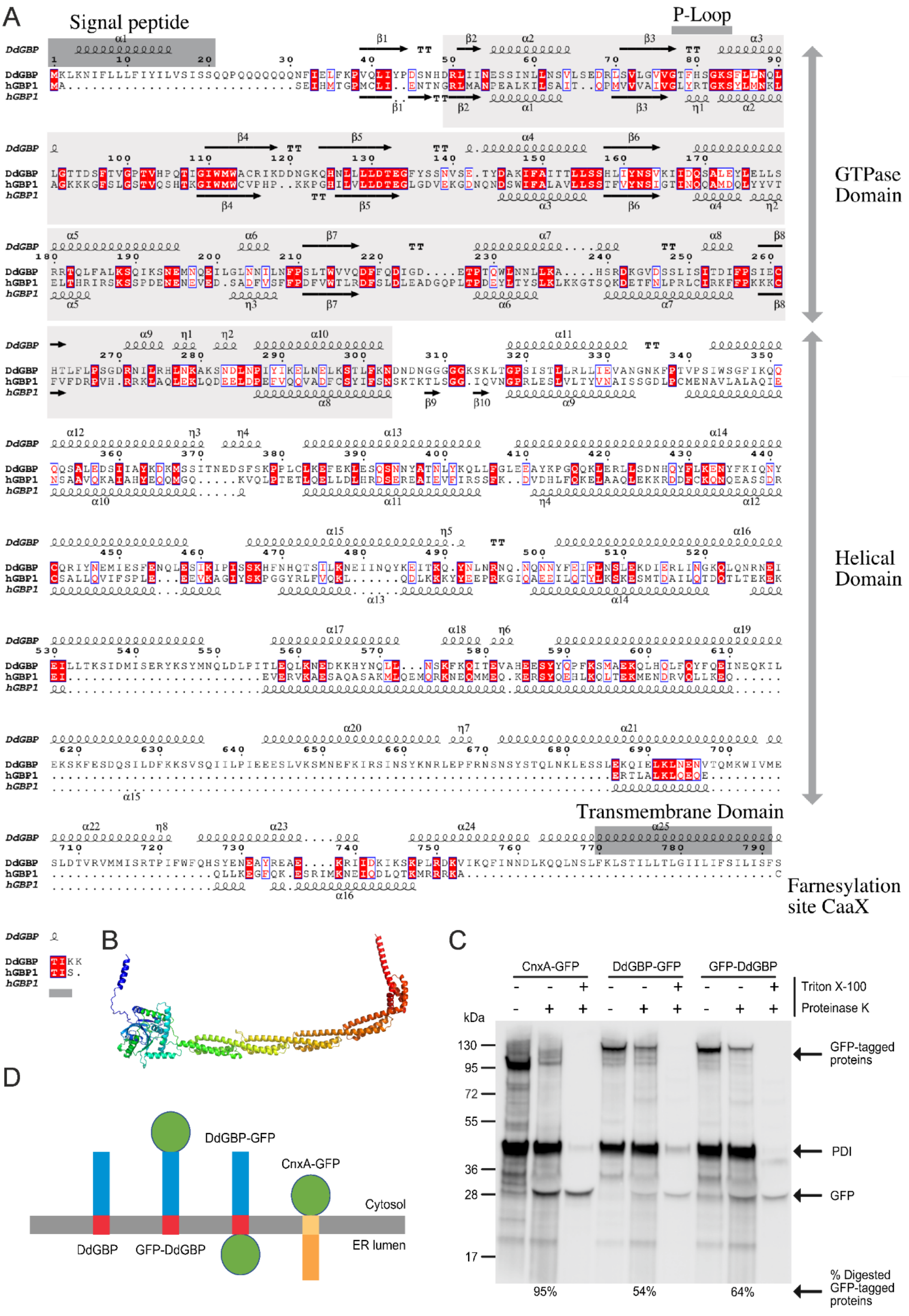
A
Signal peptide
P-Loop
GTPase Domain
Helical Domain
Transmembrane Domain
Farnesylation site CaaX
DdGBP
hGBP1
B
C
CnxA-GFP
DdGBP-GFP
GFP-DdGBP
Triton X-100
Proteinase K
kDa
130
95
72
55
36
28
17
GFP-tagged proteins
PDI
GFP
% Digested GFP-tagged proteins
95%
54%
64%
D
DdGBP-GFP
CnxA-GFP
Cytosol
DdGBP
GFP-DdGBP
ER lumen

Fig. S2. Analysis of DdGBP topology in the ER membrane. (**A**) Sequence alignment of DdGBP and human GBP1 (hGBP1) was made using T-Coffee (www.ebi.ac.uk/ jdispatcher/msa/tcoffee) and analyzed with ESPript 3.0 (espript.ibcp.fr/ESPript/ESPript/) with published (hGBP1) or predicted (DdGBP) structural data (hGBP1 – UniProtKB: P32455, PDB: 1DG3; DdGBP – UniProtKB: Q54TN9). P-Loop, GTPase, and helical domains are displayed, as well as the predicted N-terminal signal peptide and C-terminal transmembrane domains of DdGBP, and the C-terminal CaaX farnesylation motif of hGBP1. The α-helical domain is more extended in DdGBP than in hGBP1. (**B**) Structural model of DdGBP as predicted by AlphaFold3 (AlphaFoldDB: AF-Q54TN9-F1-v4): N-terminus (blue), C-terminus (red). (**C**) Proteinase K digestion (30 min) using intact or Triton X-100-treated crude membranes of *D. discoideum* producing the indicated GFP-tagged proteins. Immunoblot using anti-GFP and anti-protein disulfide isomerase (PDI) antibodies is shown (left: molecular weight markers (kDa), below: digestion (%) of GFP-tagged proteins). The data shown is representative of two independent biological replicates. (**D**) Presumed topology of GFP-tagged DdGBP and CnxA, taking into account the predicted C-terminal transmembrane domain of DdGBP.
