# Supplementary material for: Inter-kingdom signaling by the Legionella autoinducer LAI-1 involves the antimicrobial guanylate binding protein GBP
Source: PLoS Pathog. 2025 Apr 29;21(4):e1013026. doi: 10.1371/journal.ppat.1013026 (PMC12040241; doi:10.1371/journal.ppat.1013026)
Supplement: S3 Fig — D. discoideum Ax2 or Δgnbp producing GFP (pDM317) was left untreated or treated with LAI-1 (1 µM, 5 µM or 10 µM; 1 h) or DMSO (solvent control), and cell migration towards 1 mM folate (4 h) was assessed by under-agarose assay. The white lines represent the edge of the sample wells. (PDF) [file ppat.1013026.s003.pdf]

Figure S3

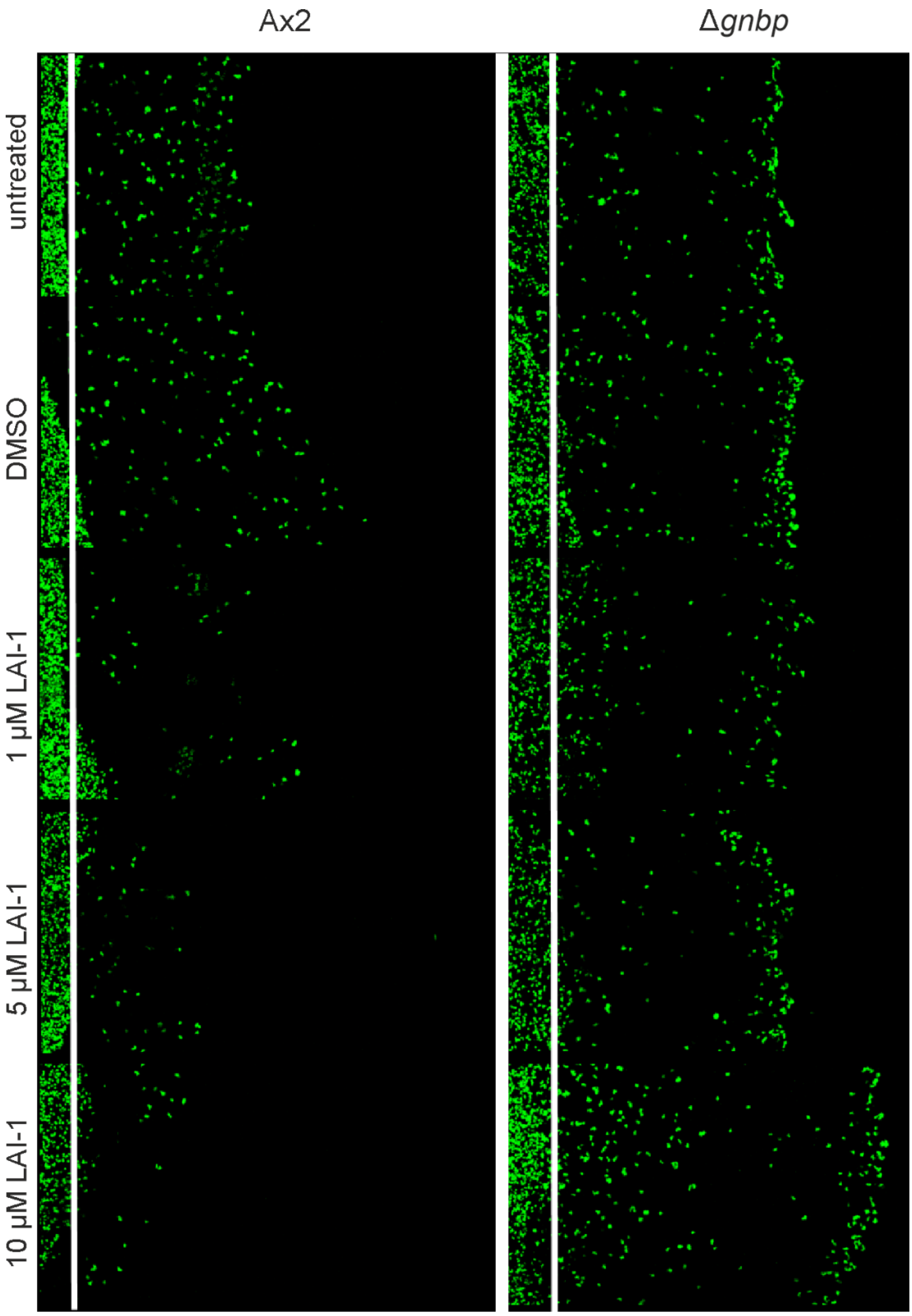

Fig. S3. LAI-1-dependent migration inhibition of *D. discoideum* involves GBP. *D. discoideum* Ax2 or Δ*gnbp* producing GFP (pDM317) was left untreated or treated with LAI-1 (1 µM, 5 µM or 10 µM; 1 h) or DMSO (solvent control), and cell migration towards 1 mM folate (4 h) was assessed by under-agarose assay. The white lines represent the edge of the sample wells.
