# Supplementary material for: Inter-kingdom signaling by the Legionella autoinducer LAI-1 involves the antimicrobial guanylate binding protein GBP
Source: PLoS Pathog. 2025 Apr 29;21(4):e1013026. doi: 10.1371/journal.ppat.1013026 (PMC12040241; doi:10.1371/journal.ppat.1013026)
Supplement: S4 Fig — (A) D. discoideum Ax2 or Δgnbp was infected (MOI 1, 10 d) with GFP-producing L. pneumophila JR32 or ΔicmT (pNT28), and intracellular replication was assessed by RFU. Data shown are means and standard deviations of biological triplicates (Student’s t-test; *, p ≤ 0.05; **, p ≤ 0.01). (B, C) D. discoideum Ax2 or Δgnbp was treated with LAI-1 (10 µM, 1 h) or DMSO (solvent control), infected (MOI 50, 30 min) with GFP-producing L. pneumophila JR32 (pNT28) and analyzed by flow cytometry. Untreated, uninfected amoebae were used for gating. Data shown are (B) counts vs. GFP fluorescence intensity, and (C) percentage of GFP-positive amoebae (means and standard deviations of biological triplicates). (PDF) [file ppat.1013026.s004.pdf]

Figure S4

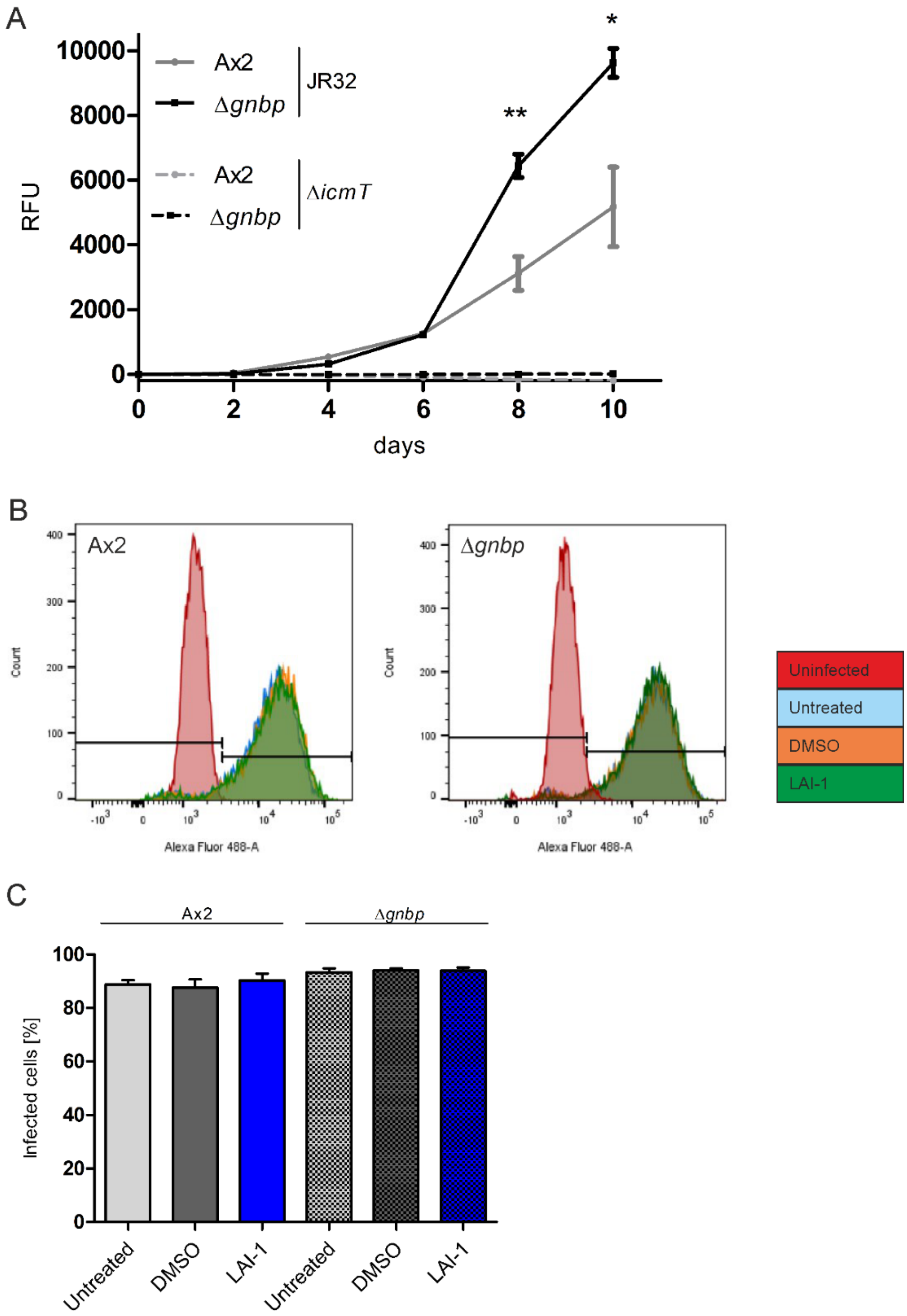

Fig. S4. *D. discoideum* Δ*gnbp* does not permit replication of *L. pneumophila* Δ*icmT* and does not affect *L. pneumophila* uptake. (**A**) *D. discoideum* Ax2 or Δ*gnbp* was infected (MOI 1, 10 d) with GFP-producing *L. pneumophila* JR32 or Δ*icmT* (pNT28), and intracellular replication was assessed by RFU. Data shown are means and standard deviations of biological triplicates (Student's t-test; *, $p \leq 0.05$; **, $p \leq 0.01$). (**B**, **C**) *D. discoideum* Ax2 or Δ*gnbp* was treated with LAI-1 (10 μM, 1 h) or DMSO (solvent control), infected (MOI 50, 30 min) with GFP-producing *L. pneumophila* JR32 (pNT28) and analyzed by flow cytometry. Untreated, uninfected amoebae were used for gating. Data shown are (B) counts vs. GFP fluorescence intensity, and (C) percentage of GFP-positive amoebae (means and standard deviations of biological triplicates).
