# Supplementary material for: Inter-kingdom signaling by the Legionella autoinducer LAI-1 involves the antimicrobial guanylate binding protein GBP
Source: PLoS Pathog. 2025 Apr 29;21(4):e1013026. doi: 10.1371/journal.ppat.1013026 (PMC12040241; doi:10.1371/journal.ppat.1013026)
Supplement: S5 Fig — (A) D. discoideum Ax2 or Δgnbp was infected (MOI 10) for the time indicated with luciferase-producing M. marinum, and intracellular growth was assessed by bioluminescence. Means and SEM of biological triplicates are shown for three independent Δgnbp clones (c3, c17, c19); r.l.u., relative light units. (B) D. discoideum Ax2 producing GBP-GFP was infected (MOI 10, 1.5 h or 24 h) with mCherry-producing M. marinum, fixed and analyzed by confocal microscopy. Representative maximum projections of live time-lapse spinning disk confocal images are shown. Scale bars, 10 µm. Images are representative of at least 3 independent experiments. (PDF) [file ppat.1013026.s005.pdf]

Figure S5

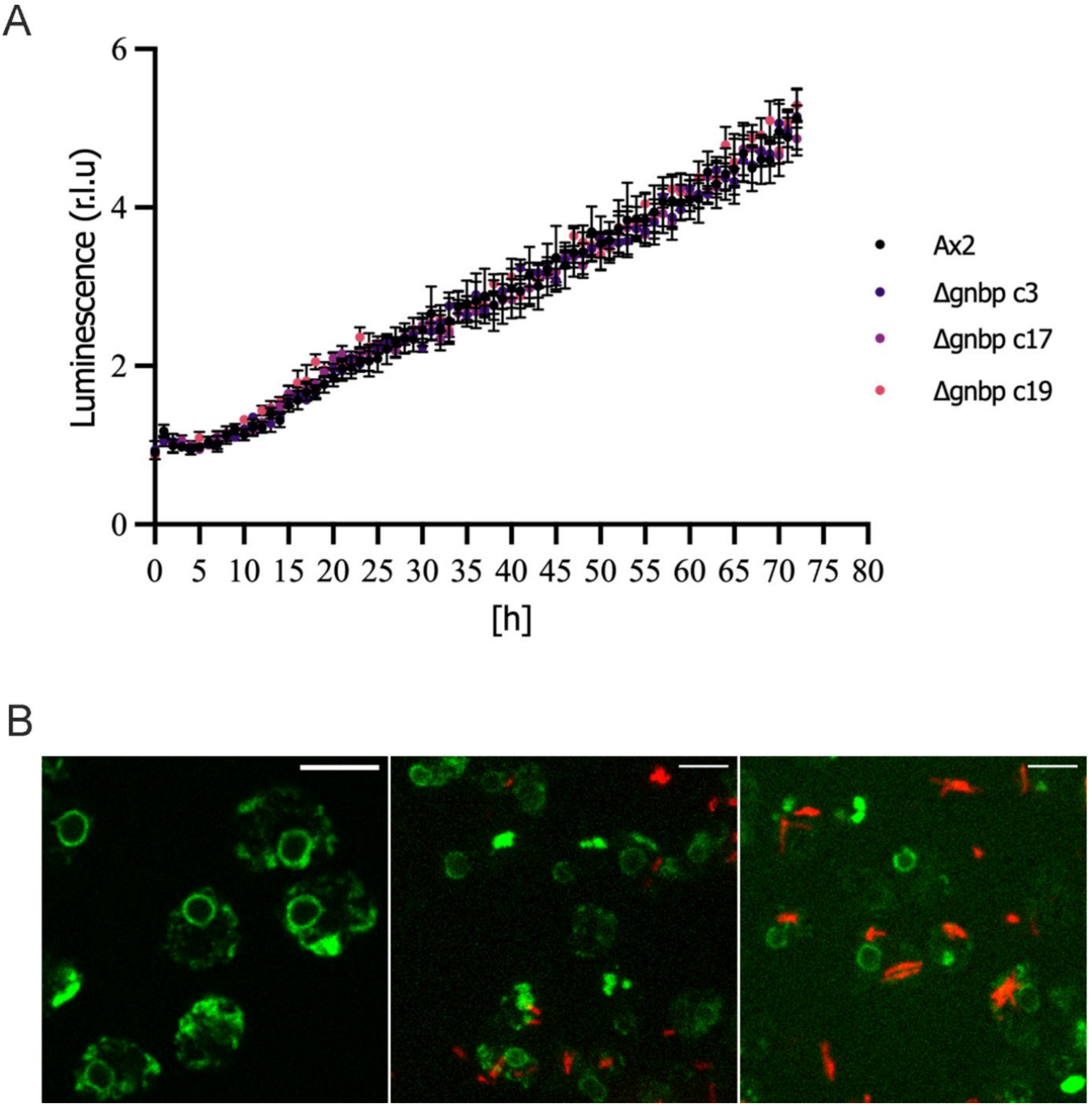

Fig. S5. GBP does not affect growth of intracellular *M. marinum*. (**A**) *D. discoideum* Ax2 or Δ*gnbp* was infected (MOI 10) for the time indicated with luciferase-producing *M. marinum*, and intracellular growth was assessed by bioluminescence. Means and SEM of biological triplicates are shown for three independent Δ*gnbp* clones (c3, c17, c19); r.l.u., relative light units. (**B**) *D. discoideum* Ax2 producing GBP-GFP was infected (MOI 10, 1.5 h or 24 h) with mCherry-producing *M. marinum*, fixed and analyzed by confocal microscopy. Representative maximum projections of live time-lapse spinning disk confocal images are shown. Scale bars, 10 µm. Images are representative of at least 3 independent experiments.
