# Supplementary material for: Inter-kingdom signaling by the Legionella autoinducer LAI-1 involves the antimicrobial guanylate binding protein GBP
Source: PLoS Pathog. 2025 Apr 29;21(4):e1013026. doi: 10.1371/journal.ppat.1013026 (PMC12040241; doi:10.1371/journal.ppat.1013026)
Supplement: S6 Fig — Dually labeled D. discoideum Ax2 producing GBP-GFP and (A) calnexin (CnxA)-mCherry (pAW012) or (B) P4C-mCherry (pWS032) was left untreated or treated with LAI-1 (10 µM, 1 h), or DMSO (solvent control), infected (MOI 5, 4 h) with mCerulean-producing L. pneumophila JR32 (pNP99), fixed, and analyzed by confocal microscopy. Scale bars, 3 µm. Single channels and merge are shown (related to Fig 5). (PDF) [file ppat.1013026.s006.pdf]

Figure S6

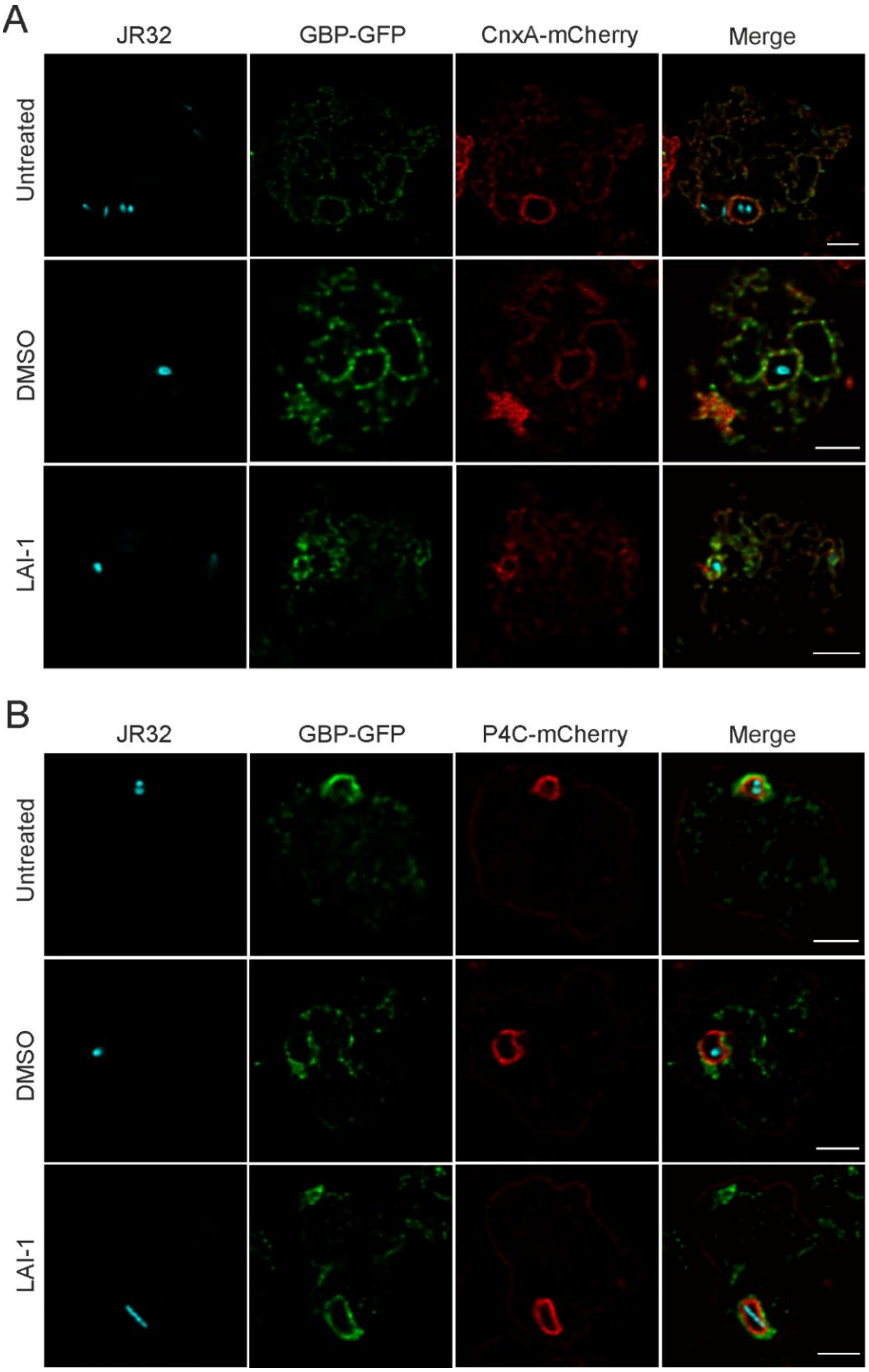

Fig. S6. GBP localizes to ER at LCV-ER contact sites. Dually labelled *D. discoideum* Ax2 producing GBP-GFP and (**A**) calnexin (CnxA)-mCherry (pAW012) or (**B**) P4C-mCherry (pWS032) was left untreated or treated with LAI-1 (10 µM, 1 h), or DMSO (solvent control), infected (MOI 5, 4 h) with mCerulean-producing *L. pneumophila* JR32 (pNP99), fixed, and analyzed by confocal microscopy. Scale bars, 3 µm. Single channels and merge are shown (related to **Fig. 5**).
