# Supplementary material for: Inter-kingdom signaling by the Legionella autoinducer LAI-1 involves the antimicrobial guanylate binding protein GBP
Source: PLoS Pathog. 2025 Apr 29;21(4):e1013026. doi: 10.1371/journal.ppat.1013026 (PMC12040241; doi:10.1371/journal.ppat.1013026)
Supplement: S7 Fig — Dually labeled D. discoideum Ax2 producing GBP-GFP and AmtA-mCherry (pDM1044-AmtA-mCherry) was left untreated or treated with LAI-1 (10 µM, 1 h), or DMSO (solvent control), infected (MOI 5, 8 h) with mCerulean-producing L. pneumophila JR32 or ΔicmT (pNP99) and analyzed by confocal microscopy. Scale bars, 3 μm. Single channels and merge are shown (related to Fig 6). (PDF) [file ppat.1013026.s007.pdf]

Figure S7

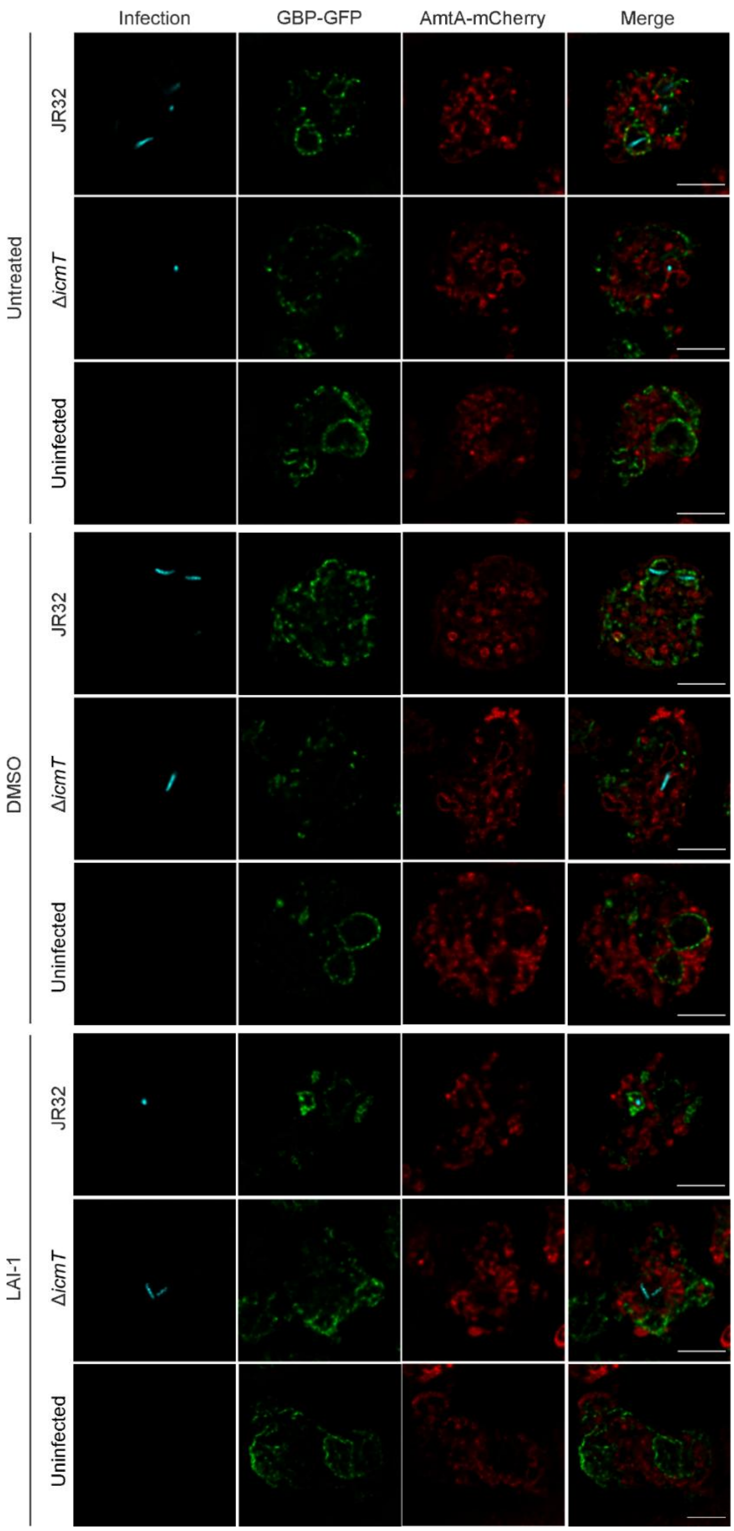

Fig. S7. LAI-1 prevents expansion of GBP-positive LCVs. Dually labelled *D. discoideum* Ax2 producing GBP-GFP and AmtA-mCherry (pDM1044-AmtA-mCherry) was left untreated or treated with LAI-1 (10 µM, 1 h), or DMSO (solvent control), infected (MOI 5, 8 h) with mCerulean-producing *L. pneumophila* JR32 or Δ*icmT* (pNP99) and analyzed by confocal microscopy. Scale bars, 3 µm. Single channels and merge are shown (related to **Fig. 6**).
