# Supplementary material for: Inter-kingdom signaling by the Legionella autoinducer LAI-1 involves the antimicrobial guanylate binding protein GBP
Source: PLoS Pathog. 2025 Apr 29;21(4):e1013026. doi: 10.1371/journal.ppat.1013026 (PMC12040241; doi:10.1371/journal.ppat.1013026)
Supplement: S8 Fig — (A) Dually labeled D. discoideum Ax2 or Δgnbp producing calnexin (CnxA)-GFP (pAW016) and P4C-mCherry (pWS032) was treated with LAI-1 (10 µM, 1 h), or DMSO (solvent control), infected (MOI 5, 4 h) with mCerulean-producing L. pneumophila JR32 (pNP99), fixed and analyzed by confocal laser microscopy. Scale bars, 3 µm. (B) D. discoideum Ax2 or Δgnbp producing CnxA-mCherry (pAW012) or P4C-mCherry (pWS032) was infected (MOI 5, 4 h) with GFP-producing L. pneumophila JR32 harboring pMF16 (P6SRNA-lqsA) or pMF17 (P6SRNA-lqsAK258A), fixed and analyzed by confocal microscopy. Scale bars, 3 µm. Single channels and merge are shown (related to Fig 7). (PDF) [file ppat.1013026.s008.pdf]

Figure S8

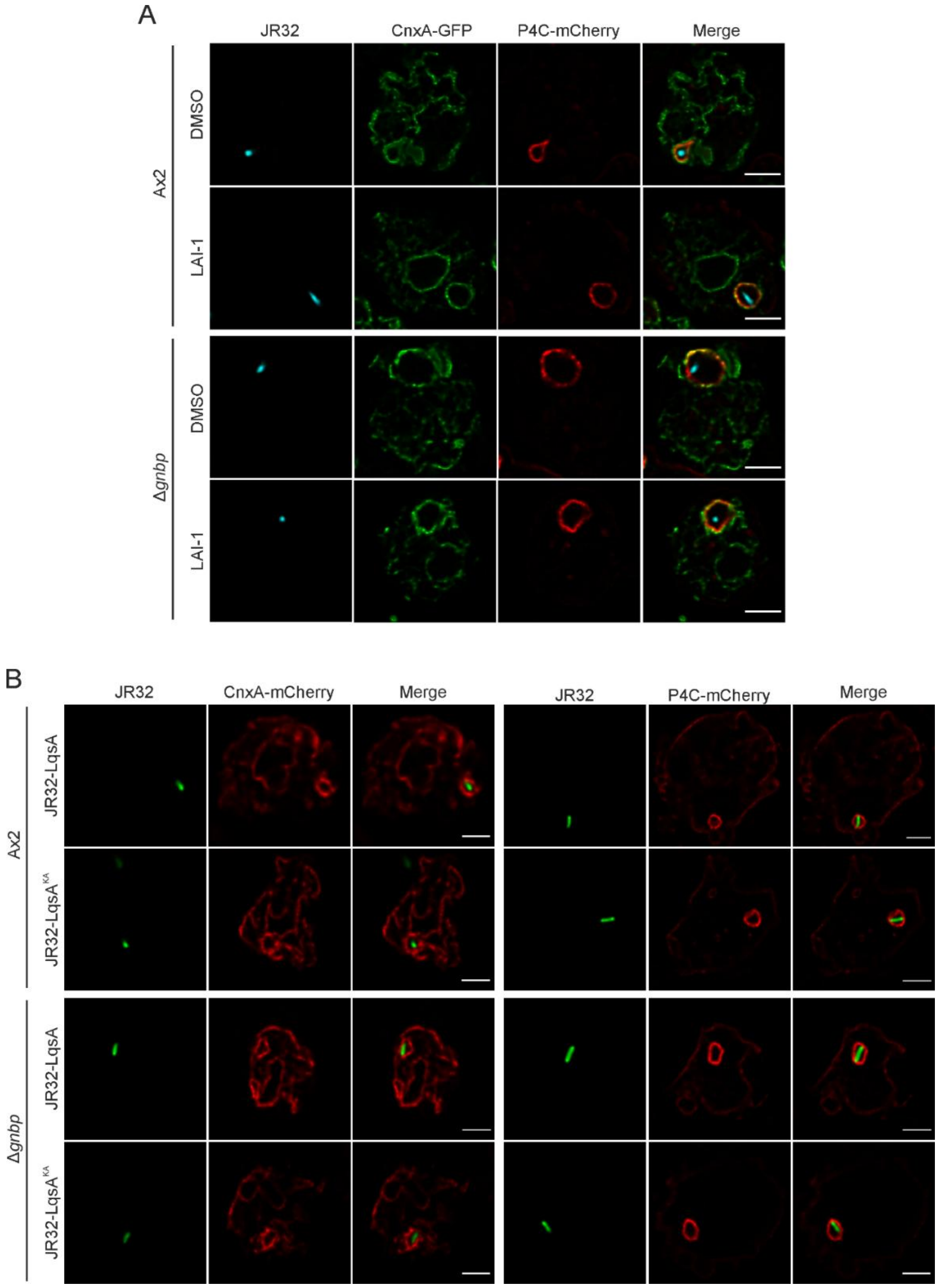

Fig. S8. LAI-1-dependent LCV remodeling involves GBP. (**A**) Dually labelled *D. discoideum* Ax2 or Δ*gnbp* producing calnexin (CnxA)-GFP (pAW016) and P4C-mCherry (pWS032) was treated with LAI-1 (10 µM, 1 h), or DMSO (solvent control), infected (MOI 5, 4 h) with mCerulean-producing *L. pneumophila* JR32 (pNP99), fixed and analyzed by confocal laser microscopy. Scale bars, 3 µm. (**B**) *D. discoideum* Ax2 or Δ*gnbp* producing CnxA-mCherry (pAW012) or P4C-mCherry (pWS032) was infected (MOI 5, 4 h) with GFP-producing *L. pneumophila* JR32 harboring pMF16 (P_{6SRNA}-*lqsA*) or pMF17 (P_{6SRNA}-$lqsA^{K258A}$), fixed and analyzed by confocal microscopy. Scale bars, 3 µm. Single channels and merge are shown (related to **Fig. 7**).
