# Supplementary material for: Inter-kingdom signaling by the Legionella autoinducer LAI-1 involves the antimicrobial guanylate binding protein GBP
Source: PLoS Pathog. 2025 Apr 29;21(4):e1013026. doi: 10.1371/journal.ppat.1013026 (PMC12040241; doi:10.1371/journal.ppat.1013026)
Supplement: S9 Fig — Dually labeled D. discoideum Ax2 or Δgnbp producing cytoplasmic mCherry (pDM1042) and P4C-GFP (pWS034) was left untreated or treated with LAI-1 (10 µM, 1 h), or DMSO (solvent control), infected (MOI 5, 4 h) with mCerulean-producing L. pneumophila JR32 (pNP99) and analyzed by confocal microscopy. Scale bars, 3 μm. Single channels and merge are shown. (PDF) [file ppat.1013026.s009.pdf]

Figure S9

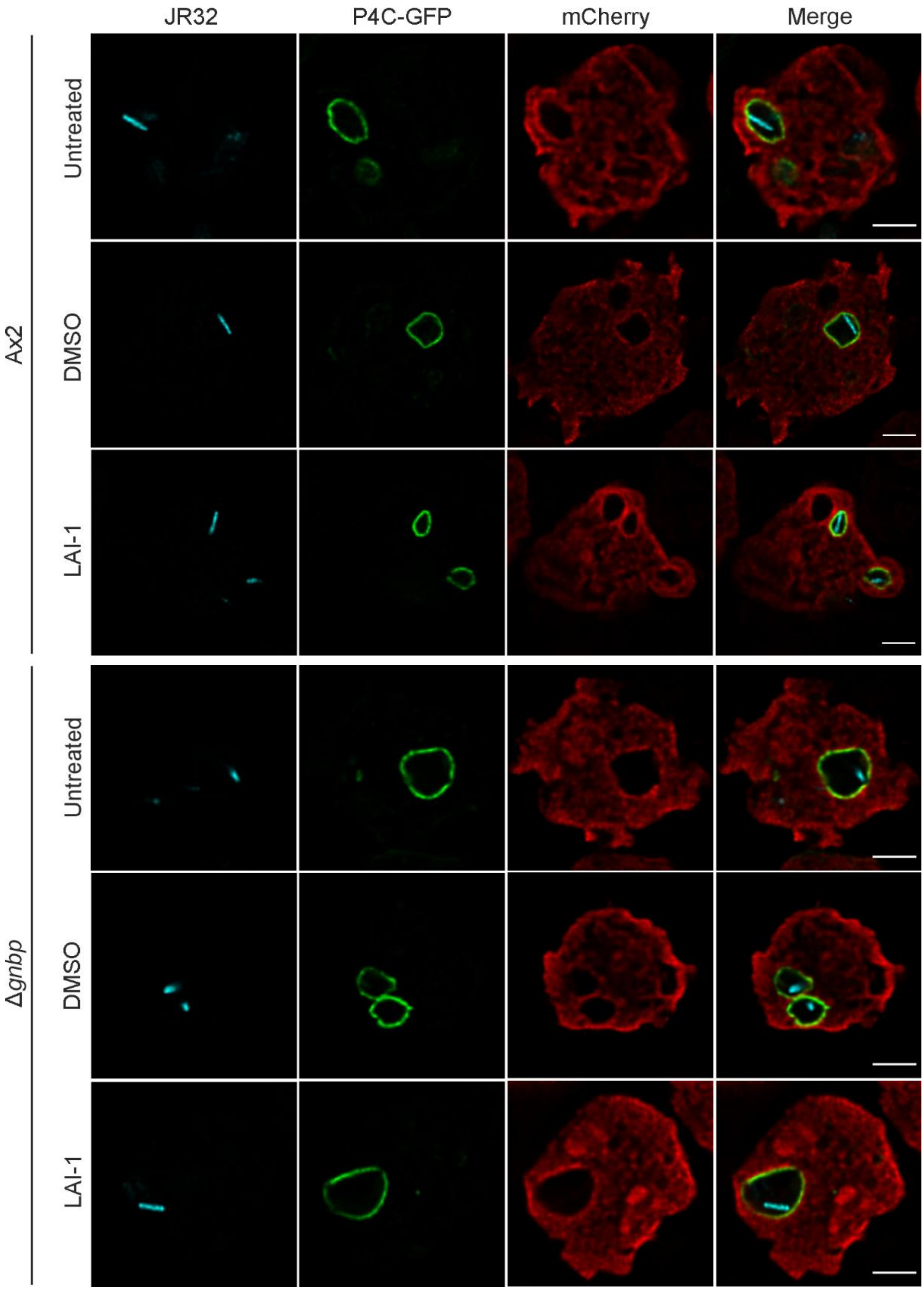

Fig. S9. LAI-1 and GBP do not affect LCV integrity. Dually labelled *D. discoideum* Ax2 or Δ*gnbp* producing cytosolic mCherry (pDM1042) and P4C-GFP (pWS034) was left untreated or treated with LAI-1 (10 µM, 1 h), or DMSO (solvent control), infected (MOI 5, 4 h) with mCerulean-producing *L. pneumophila* JR32 (pNP99) and analyzed by confocal microscopy. Scale bars, 3 µm. Single channels and merge are shown.
