# Supplementary material for: Inter-kingdom signaling by the Legionella autoinducer LAI-1 involves the antimicrobial guanylate binding protein GBP
Source: PLoS Pathog. 2025 Apr 29;21(4):e1013026. doi: 10.1371/journal.ppat.1013026 (PMC12040241; doi:10.1371/journal.ppat.1013026)
Supplement: S10 Fig — (A) Reagents and conditions: a) HNMe(OMe)•HCl (1.15 eq.), NMM (1.15 eq.), EDC•HCl (1.15 eq.), CH2Cl2 –15 °C→rt, 65 h, quant.; b) TBDPSCl (1.15 eq.), imidazole (4.60 eq.), DMF, 0 °C→rt→55 °C, 85%; c) DHP (1.50 eq.), PpTs (0.10 eq.), CH2Cl2, 0 °C→rt, 19 h, 99%; d), Mg (8.00 eq.), 4 (2.10 eq.), THF, 0 °C→rt, 16 h, 56%; e) PpTs (0.30 eq.), THF:MeOH (3:1), 60 °C, 20 h, 92%; f) CBr4 (1.50 eq.), PPh3 (1.50 eq.), CH2Cl2, 0 °C→rt, 17 h, 99%; NaN3 (3.00 eq.), DMF, 60 °C, 16 h, quant.; h) TBAF (1 m in THF, 1.20 eq.), THF, 0 °C→rt, 1.5 h, 90%. DHP = 3,4-Dihydro-2H-pyran, DMF = N,N-Dimethylformamide, EDC = 1-Ethyl-3-(3-dimethylaminopropyl)carbodiimide, eq. = equivalents, NMM = 4-Methylmorpholine, PpTs = pyridinium p-toluenesulfonate, quant. = quantitative; TBAF = tetra-n-butylammonium fluoride; TBDPSCl = tert-butyldiphenylsilyl chloride, THF = tetrahydrofuran. (B) Azido-LAI-1 can be attached to various conjugation partners (e.g., dyes) using SPAAC. (PDF) [file ppat.1013026.s010.pdf]

Figure S10

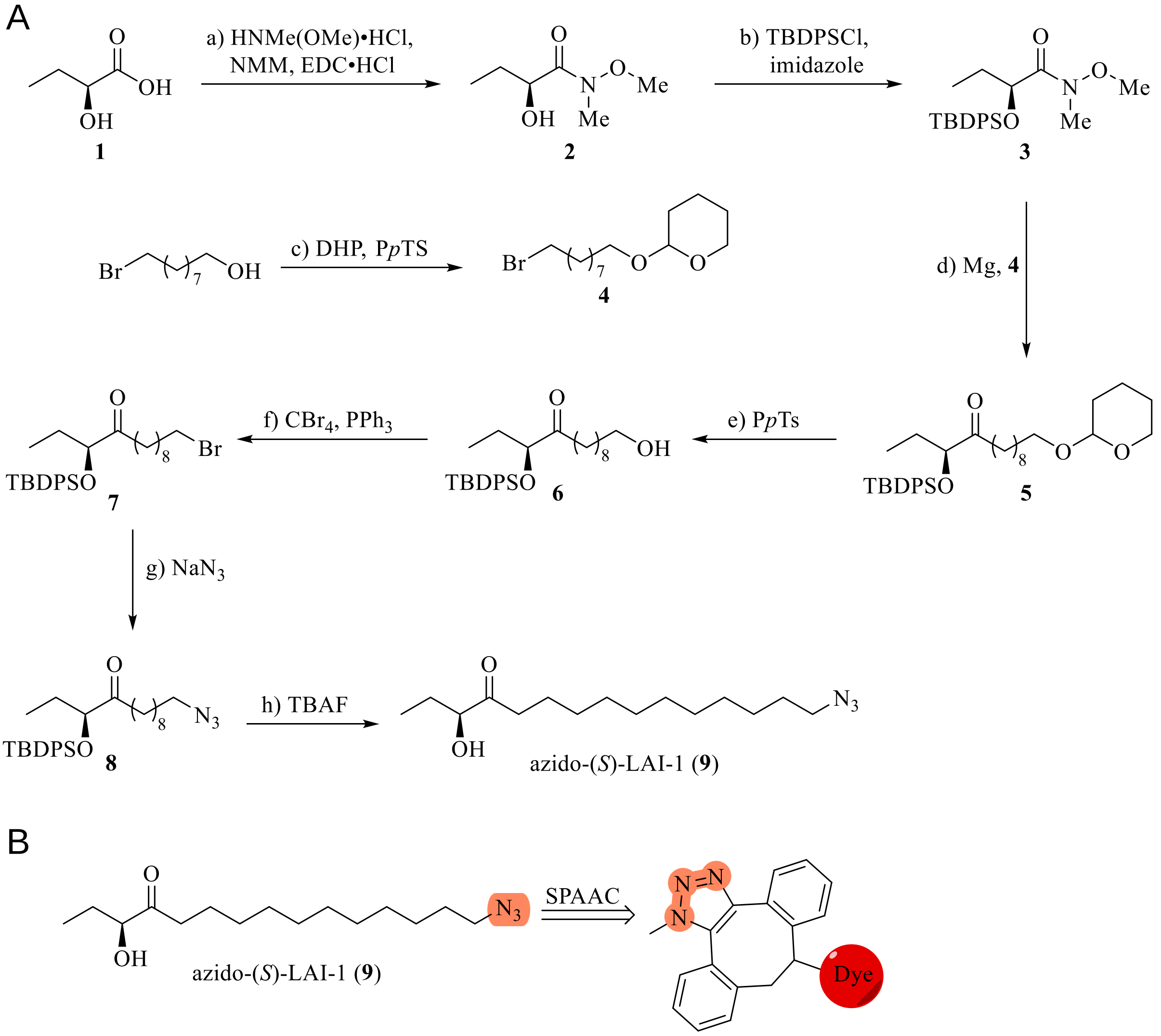

Fig. S10. Synthesis and application of azido-(*S*)-LAI-1. (**A**) Reagents and conditions: a) HNMe(OMe)•HCl (1.15 eq.), NMM (1.15 eq.), EDC•HCl (1.15 eq.), CH_2Cl_2 –15 °C→rt, 65 h, quant.; b) TBDPSCl (1.15 eq.), imidazole (4.60 eq.), DMF, 0 °C→rt→55 °C, 85%; c) DHP (1.50 eq.), P*p*Ts (0.10 eq.), CH_2Cl_2, 0 °C→rt, 19 h, 99%; d), Mg (8.00 eq.), **4** (2.10 eq.), THF, 0 °C→rt, 16 h, 56%; e) P*p*Ts (0.30 eq.), THF:MeOH (3:1), 60 °C, 20 h, 92%; f) CBr_4 (1.50 eq.), PPh_3 (1.50 eq.), CH_2Cl_2, 0 °C→rt, 17 h, 99%; NaN_3 (3.00 eq.), DMF, 60 °C, 16 h, quant.; h) TBAF (1 M in THF, 1.20 eq.), THF, 0 °C→rt, 1.5 h, 90%. DHP = 3,4-Dihydro-2*H*-pyran, DMF = *N*,*N*-Dimethylformamide, EDC = 1-Ethyl-3-(3-dimethylaminopropyl) carbodiimide, eq. = equivalents, NMM = 4-Methylmorpholine, P*p*Ts = pyridinium *p*-toluenesulfonate, quant. = quantitative, TBAF = tetra-*n*-butylammonium fluoride; TBDPSCl = *tert*-butyldiphenylsilyl chloride, THF = tetrahydrofuran. (**B**) Azido-LAI-1 can be attached to various conjugation partners (e.g., dyes) using SPAAC.
