# Supplementary material for: Inter-kingdom signaling by the Legionella autoinducer LAI-1 involves the antimicrobial guanylate binding protein GBP
Source: PLoS Pathog. 2025 Apr 29;21(4):e1013026. doi: 10.1371/journal.ppat.1013026 (PMC12040241; doi:10.1371/journal.ppat.1013026)
Supplement: S11 Fig — (A) Reagents and conditions: a) DHP (1.50 eq.), PpTs (0.10 eq.), CH2Cl2, 0 °C→rt, 18 h, 95%; b) Mg (8.00 eq.), 10 (2.10 eq.), THF, 0 °C→rt, 16 h, 91%; c) PpTs (0.25 eq.), THF:MeOH (3:1), 60 °C, 20 h, 91%; d) CSA (1.00 eq.), 1,2-ethanediol, (24.6 eq.), ethyl orthoformate (8.30 eq.), 50 °C, 16 h, 74%; e) PDC (3.50 eq.), DMF, rt, 16 h, quant.; f) HNMe(OMe)•HCl (1.15 eq.), NMM (1.15 eq.), EDC•HCl (1.15 eq.), CH2Cl2 0 °C→rt, 49 h, 72%; g) DHP (1.50 eq.), PpTs (0.10 eq.), CH2Cl2, 0 °C→rt, 18 h, 87%; h) Mg (8.00 eq.), 16 (2.35 eq.), THF, 0 °C→rt, 17 h, 91%; i) NH3 (7 m in MeOH, 75 eq.), HOSA (1.15 eq), MeOH, 0 °C→rt, 51 h; j) I2 (1.25 eq.), NEt3 (2.00 eq.), MeOH, 0 °C→rt, 16 h, 42% over two steps; k) I2 (0.10 eq.), acetone (58.4 eq.), 60 °C, 10 min; l)) PpTs (0.25 eq.), THF:MeOH (3:1), 60 °C, 55 h, 89% over two steps; m) CBr4 (1.50 eq.), PPh3 (1.50 eq.), CH2Cl2, 0 °C→rt, 17 h, 73%; n) NaN3 (3.00 eq.), DMF, 60 °C, 16 h, 99%; o) TBAF (1 m in THF, 1.20 eq.), THF, 0 °C→rt, 1.5 h, 85%. CSA = camphorsulfonic acid, PDC = pyridinium dichromate, HOSA = hydroxylamine-O-sulfonic acid. (B) The diazirine function of azido-diazirine-LAI-1 is stimulated by UV light and forms a carbene by releasing nitrogen. The highly reactive carbene can interact with various chemical moieties and thus covalently binds to its biological environment. The covalently fixed azido-LAI-1-derivative can then be attached to various conjugation partners (e.g., dyes, biotin) using SPAAC. (PDF) [file ppat.1013026.s011.pdf]

Figure S11

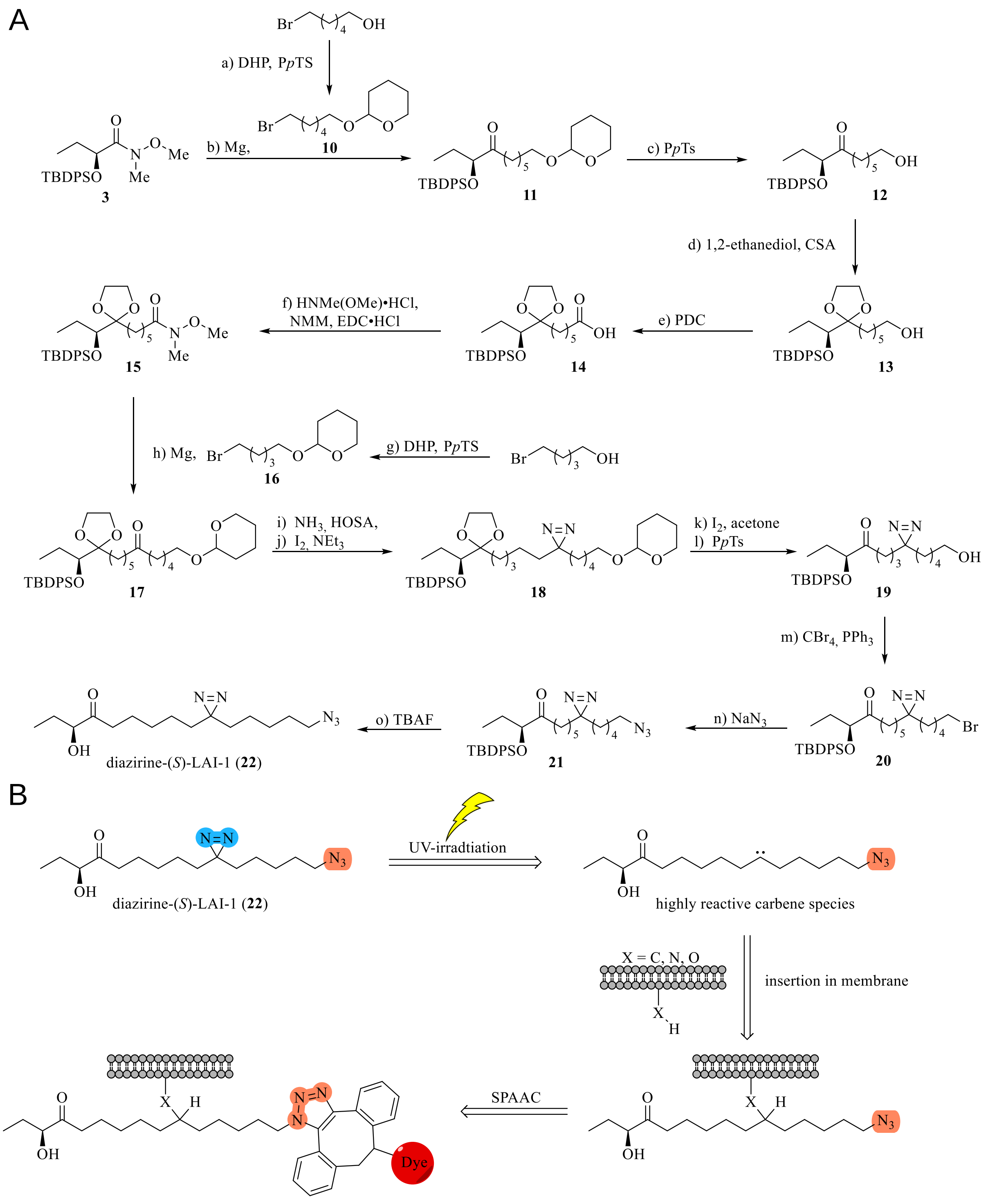

Fig. S11. Synthesis and application of diazirine-azido-(*S*)-LAI-1. (**A**) Reagents and conditions: a) DHP (1.50 eq.), P*p*Ts (0.10 eq.), CH_2Cl_2, 0 °C→rt, 18 h, 95%; b) Mg (8.00 eq.), **10** (2.10 eq.), THF, 0 °C→rt, 16 h, 91%; c) P*p*Ts (0.25 eq.), THF:MeOH (3:1), 60 °C, 20 h, 91%; d) CSA (1.00 eq.), 1,2-ethanediol, (24.6 eq.), ethyl orthoformate (8.30 eq.), 50 °C, 16 h, 74%; e) PDC (3.50 eq.), DMF, rt, 16 h, quant.; f) HNMe(OMe)•HCl (1.15 eq.), NMM

(1.15 eq.), EDC•HCl (1.15 eq.), CH_2Cl_2 0 °C→rt, 49 h, 72%; g) DHP (1.50 eq.), P*p*Ts (0.10 eq.), CH_2Cl_2, 0 °C→rt, 18 h, 87%; h) Mg (8.00 eq.), **16** (2.35 eq.), THF, 0 °C→rt, 17 h, 91%; i) NH_3 (7 M in MeOH, 75 eq.), HOSA (1.15 eq), MeOH, 0 °C→rt, 51 h; j) I_2 (1.25 eq.), NEt_3 (2.00 eq.), MeOH, 0 °C→rt, 16 h, 42% over two steps; k) I_2 (0.10 eq.), acetone (58.4 eq.), 60 °C, 10 min; l)) P*p*Ts (0.25 eq.), THF:MeOH (3:1), 60 °C, 55 h, 89% over two steps; m) CBr_4 (1.50 eq.), PPh_3 (1.50 eq.), CH_2Cl_2, 0 °C→rt, 17 h, 73%; n) NaN_3 (3.00 eq.), DMF, 60 °C, 16 h, 99%; o) TBAF (1 M in THF, 1.20 eq.), THF, 0 °C→rt, 1.5 h, 85%. CSA = camphorsulfonic acid, PDC = pyridinium dichromate, HOSA = hydroxylamine-*O*-sulfonic acid. (**B**) The diazirine function of diazirine-azido-LAI-1 is stimulated by UV light and forms a carbene by splitting off nitrogen. The highly reactive carbene can interact with various chemical bonds and thus form a covalent bond with the biological environment. The fixed molecule can then be attached to various conjugation partners (e.g., dyes, biotin) using SPAAC.
