# Supplementary material for: Inter-kingdom signaling by the Legionella autoinducer LAI-1 involves the antimicrobial guanylate binding protein GBP
Source: PLoS Pathog. 2025 Apr 29;21(4):e1013026. doi: 10.1371/journal.ppat.1013026 (PMC12040241; doi:10.1371/journal.ppat.1013026)
Supplement: S1 Table — (PDF) [file ppat.1013026.s012.pdf]

Table S1. Oligonucleotides used in this study.

Primer	Sequence (5'-3')	Purpose
LR5F	CAAGATCTAAAATGAAATTAAAAAACATTTTTTTG	*gnbp* 5' BglII
LR5R	AGTCTAGATTTTTTAATAGTTGAAAATG	*gnbp* 3' XbaI
LR28F	CAGCATTTTTAGGTCAAGTTG	*gnbp* internal primer
LR28R	GTAGTTTAATTTCAATCAC	*gnbp* internal primer
LR36F	AGCGCGTCTCCAATGCTGCAGCCAAATATAAAGTCACAAAGTC	*gnbp* deletion left arm; CS1, PstI
LR36R	AGCGCGTCTCCGTTGGTACTGATAATCTATCTTCACTC	*gnbp* deletion left arm; CS2
LR37F	AGCGCGTCTCCCTTCGAGGAATCATTGGTTAAATCAATG	*gnbp* deletion right arm; CS3
LR37R	AGCGCGTCTCCTCCCCTGCAGCAATCACCATCAACTGAAC	*gnbp* deletion right arm; CS4, PstI
LR38F	GGTGAATTTGAAAGGTCAAC	*gnbp* gDNA upstream of deletion left arm
LR39R	GTCCAAATGATGAAATCGTTC	*gnbp* gDNA downstream of deletion right arm
LR46F	GATTTGATGGGATTAATTAATTTGTAATC	pKOSG-IBA-dicty1 actin promoter
LR46R	GAAAATCAAAAAGATAAAGCTGAC	pKOSG-IBA-dicty1 actin terminator
Bls-F	AGATCTTGTTGAGAAATGTTAAATTGATC	5' of the Bls cassette
Bls-R	TTGAAGAACTCATTCCACTCAAATATAC	3' of the Bls cassette
