# Supplementary material for: Inter-kingdom signaling by the Legionella autoinducer LAI-1 involves the antimicrobial guanylate binding protein GBP
Source: PLoS Pathog. 2025 Apr 29;21(4):e1013026. doi: 10.1371/journal.ppat.1013026 (PMC12040241; doi:10.1371/journal.ppat.1013026)
Supplement: S1 Text — (PDF) [file ppat.1013026.s013.pdf]

Experimental Procedures

Abbreviations

CSA, camphorsulfonic acid; Cy, cyclohexane; DCM, dichloromethane; DHP, 3,4-Dihydro-2*H*-pyran; DMF, *N*,*N*-Dimethylformamide; EDC, 1-Ethyl-3-(3-dimethylaminopropyl) carbodiimide; EtOAc, ethyl acetate; eq., equivalents; HOSA, hydroxylamine-*O*-sulfonic acid; NMM, 4-Methylmorpholine; PDC, pyridinium dichromate; P*p*Ts, pyridinium *p*-toluenesulfonate; SPAAC, strain-promoted alkyne-azide cycloadditions; TBAF, tetra-*n*-butylammonium fluoride; TBDPSCl, *tert*-butyldiphenylsilyl chloride; THF, tetrahydrofuran.

Chemicals and solvents

The chemicals used in this work were purchased from commercial sources (*Sigma Aldrich*, *VWR*, *BLD Pharm, Apollo Scientific*) or from the university chemical supply. If not otherwise specified, the utilized chemicals were used without further purification. Unless otherwise noted, all reactions were performed in flame-dried glassware under an atmosphere of nitrogen using dried reagents and solvents. Solvents were distilled prior to usage and dry solvents (DCM, DMF, THF) were obtained from the solvent purification system (*PureSolv MD-5* by *Inert*) or bought at *Sigma Aldrich*. The inert gas used was nitrogen with a purity of 99.996 % supplied by *Linde*.

Chromatography

All reactions were controlled using thin layer chromatography (TLC). This was conducted on precoated aluminum plates with silica gel 60 F254 purchased from MERCK (*ALUGRAM® Xtra SIL G/UV254*). The eluent composition is given in volume percent (v/v). To detect the substances, fluorescence quenching with a UV lamp by *Hartenstein* at 254 nm and 365 nm was used and also a staining solution of potassium permanganate (1.50 g $KMnO_4$, 10.0 g K_2CO_3, and 100 mg NaOH in 200 mL dest. H_2O) for visualisation of non-UV active species.

The chromatography columns were packed with silica gel 60 with a particle size between 0.040–0.063 mm (*Macherey-Nagel*). Eluent composition is given in volume percent (v/v). The distinct fractions could be detected by TLC.

Nuclear magnetic resonance (NMR) spectroscopy

NMR spectra were recorded with a BRUKER Avance III HD 400 spectrometer with a resonance frequency of 400 MHz for ^{1}H-NMR and 100 MHz for ^{13}C-NMR spectra. Chemical shifts are given in parts per million (ppm, δ-scale) in reference to the solvent signals of $CDCl_3$ (7.26 ppm in ^{1}H and 77.16 ppm in ^{13}C). Coupling constants (*J*) are given in Hertz (Hz), the coupling patterns are abbreviated as following: singlet (s), doublet (d), triplet (t), quartet (q), multiplet (m) and combination of those such as doublet of doublet (dd), doublet of doublet of doublet (ddd), doublet of quadruplet of doublet (dqd) and doublet of triplet (dt). Throughout this work, the numbering of the hydrogens and carbons of the different molecules does not correspond to the IUPAC numbering. Signal assignment was accomplished with additional information from DEPT135, $^{1}H,^{1}H$-COSY, $^{1}H,^{13}C$-HSQC and $^{1}H,^{13}C$-HMBC measurements.

Mass spectrometry

Mass spectra (for structure confirmation after chemical synthesis) were recorded with a *Bruker Daltonics micrOTOF* and *micrOTOF-Q III* spectrometer by electrospray ionization (ESI). The detected mass-to-charge ratio (m/z) is given and compared to the calculated monoisotopic mass.

(*S*)-2-Hydroxy-*N*-methoxy-*N*-methylbutanamide (2)

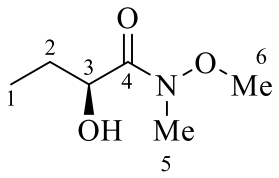

2

A solution of (*S*)-2-hydroxybutyric acid (12.4 g, 119 mmol, 1.00 eq.) in DCM (250 mL) and *N*-methylmorpholine (15.5 mL, 139 mmol, 1.15 eq.) was cooled to –15 °C and *N,O*-dimethylhydroxylamine hydrochloride (13.4 g, 137 mmol, 1.15 eq.) was added. The suspension was stirred for 45 min before EDC•HCl (21.3 g, 137 mmol, 1.15 eq.) was added portionwise. The reaction was stirred for further 3 h at approx. –15 °C and then stirred for 62 h at room temperature. The reaction mixture was poured onto approx. ice (125 g) and 1 M HCl (350 mL) was added. The mixture was extracted with DCM (6 x 150 mL), the combined organic extracts were washed with brine (200 mL) and dried over $MgSO_4$. Concentration in *vacou* afforded Weinreb amide **2** as colorless liquid (17.5 g, 119 mmol, quant.).

^{1}H (400 MHz, $CDCl_3$): δ = 4.36–4.34 (m, 1H, *H*-3), 3.71 (s, 3H, *H*-6), 3.24 (s, 3H, *H*-5), 1.83–1.73 (m, 1H, *H*-2$_a$), 1.62–1.51 (m, 1H, *H*-2$_b$), 0.97 (, ^3J = 7.44 Hz, 3H, *H*-1) ppm;

^{13}C (100 MHz, $CDCl_3$): δ = 175.2 (C-4), 69.8 (C-3), 61.4 (C-6), 53.6 (C-5), 27.8 (C-2), 9.3 (C-1) ppm;

MS (ESI pos.): *m/z* calcd. for $C_6H_{13}NNaO_3^+$ $[M+Na]^+$ 170.0788, found. 170.0793, (|Δ*m/z*| = 3.06 ppm).

(*S*)-2-((*tert*-Butyldiphenylsilyl)oxy)-*N*-methoxy-*N*-methylbutanamide (3)

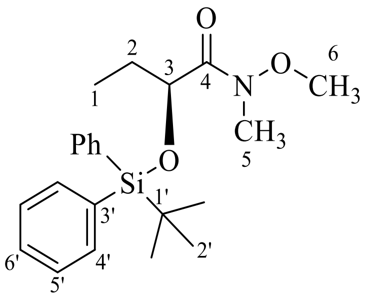

3

A solution of Weinreb amide **2** (17.5 g,119 mmol, 1.00 eq.) and imidazole (37.3 g, 548 mmol, 4.60 eq.) in DMF (125 mL) was cooled to 0 °C and a solution of TBDPSCl (37.8 g, 137 mmol, 1.15 eq.) in DMF (100 mL) was cannulated portionwise over 40 min to the reaction mixture. The reaction was stirred for 3 h at 0 °C, 20 h at room temperature and finally 3 h at 55 °C. The reaction mixture was terminated by adding sat. aq. NH_4Cl (1 L). The aqueous phase was extracted with DCM (7 x 250 mL). The combined organic extracts were concentrated under reduced pressure to approx. 1 L and then washed with water (2 x 200 mL) and brine (400 mL). The organic phase was dried over $MgSO_4$ and concentrated in *vacuo* at 75 °C. The crude product was purified by flash column chromatography (silica gel, Cy:EtOAc, 9:1) afforded silyl ether **3** (38.9 g, 101 mmol, 85%) as a colorless liquid.

^{1}H (400 MHz, $CDCl_3$): δ = 7.71–7.68 (m, 4H, *H*-4'), 7.44–7.33 (m, 6H, *H*-5', *H*-6'), 4.44–4.41 (m, 1H, *H*-3), 3.10 (bs, 3H, *H*-6), 2.99 (s, 3H, *H*-6), 1.82–1.67 (m, 2H, *H*-2), 1.09 (s, 9H, *H*-2'), 0.91 (t, ^3J = 7.46 Hz, 3H, *H*-1) ppm.

^{13}C (100 MHz, $CDCl_3$): δ = 136.3, 136.1 (*C*-4'), 134.0, 133.7 (*C*-3'), 129.7, 129.7 (*C*-6'), 127.6, 127.5 (*C*-5'), 71.1 (*C*-3), 60.7 (*C*-6), 32.3 (*C*-5), 27.9 (*C*-2), 27.0 (*C*-2'), 19.6 (*C*-1') ppm.

MS (ESI pos.): *m/z* calcd. for $C_{22}H_{31}NNaO_3Si^+$ $[M+Na]^+$ 408.1965, found 408.1954, ($|\Delta m/z|$ = 2.76 ppm).

2-((11-Bromoundecyl)oxy)tetrahydro-2*H*-pyran (4)

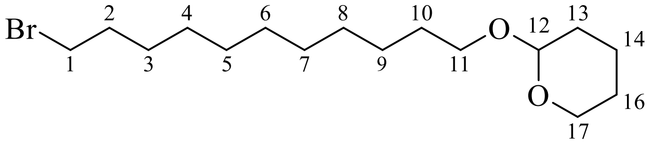

4

A solution of 11-bromundecane-1-ol (10.0 g, 39.8 mmol, 1.00 eq.) and P*p*Ts (1.00 g, 3.98 mmol, 0.10 eq.) in DCM (120 mL) was cooled to 0 °C and dihydropyran (5.68 mL, 59.7 mmol, 1.50 eq.) was added dropwise. The reaction was allowed to warm slowly to room temperature under exclusion of light in an ice bath and stirred for 19 hours. The solution was concentrated in *vacou* and the residue was purified by flash column chromatography (silica gel, Cy:EtOAc, 9:1) to afford acetal **4** (13.3 g, 39.5 mmol, 99%) as a colorless liquid.

^{1}H (400 MHz, $CDCl_3$): δ = 4.58–4.56 (m, 1H, *H*-12), 3.90–3.84 (m, 1H, *H*-16_a), 3.75–3.70 (m, 1-H, *H*-11_a), 3.52–3.47 (m, 1H, *H*-16_b), 3.43–3.36 (m, 3H, *H*-1, *H*-11_b), 1.88–1.80 (m, 3H, *H*-2, *H*-14_a), 1.75–1.68 (m, 1H, *H*-13_a), 1.62–1.50 (m, 1H, *H*-10, *H*-13_b, *H*-14_b, *H*-15), 1.43–1.25(m, 14H, *H*-3–H-9) ppm;

^{13}C (100 MHz, $CDCl_3$): δ = 99.0 (1C, *C*-12), 67.8 (1C, *C*-11), 62.5 (1C, *C*-16), 34.2 (1C, *C*-1), 33.0 (1C, *C*-2), 30.9 (1C, *C*-13), 29.9 (1C, *C*-14), 29.7, 29.6, 29.6, 28.9, 28.3 (*C*-3–*C*-8) 26.4 (1C, *C*-9), 25.6 (1C, *C*-15), 19.9 (1C, *C*-14) ppm;

MS (ESI pos.): *m/z* calcd. for $C_{16}H_{31}BrNaO_2^+$ $[M+Na]^+$ 357.1400, found 357.1388, ($|\Delta m/z|$ = 3.25 ppm).

(3*S*)-3-((*tert*-Butyldiphenylsilyl)oxy)-15-((tetrahydro-2*H*-pyran-2-yl)oxy)pentadecan-4-one (5)

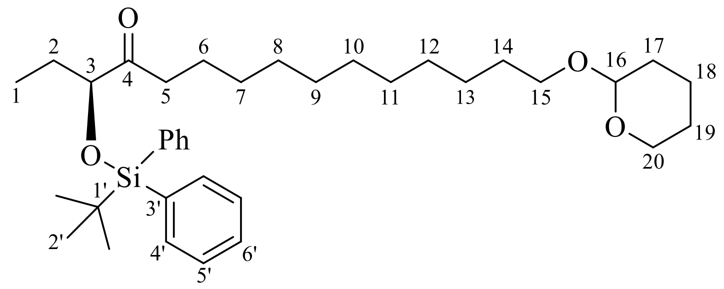

5

Magnesium turnings (595 mg, 25.5 mmol, 8.00 eq.) were activated with a bead of iodine over night. The magnesium turnings were overlaid with THF (3 mL) and then a solution of bromide **6** (2.16 g, 6.43 mmol, 2.10 eq.) in THF (8 mL) was added at room temperature dropwise over 30 min. The reaction mixture was then stirred for another 45 min at room temperature and 4 h at 55 °C. After the Grignard solution had cooled to room temperature, it was diluted with another 10 mL THF. The freshly prepared Grignard solution was added dropwise over 1.5 h to a solution of Weinreb amide **3** (1.18 g, 55.5 mmol, 1.00 eq) in THF (20 mL) at 0 °C. The reaction mixture was thawed in an ice bath and stirred for 16 hours. Under ice bath cooling, the reaction was stopped and acidified by adding 1 M $NaHSO_4$ (15 mL) solution. The reaction mixture was stirred for another 30 min until the resulting solid had dissolved. Another portion of ice (10 g) and cold Et_20 (50 mL) were added to the mixture. The organic phase was separated, and the aqueous phase was extracted with Et_20 (4 x 40 mL). The combined organic extracts were washed with 1 M $NaHSO_4$ solution (2 x 50 mL) and brine (50 mL), dried over $MgSO_4$ and concentrated in *vacuo*. The oily crude product was purified by flash column chromatography (silica gel, Cy:EtOAc, 99:1 →98:2) to afford ketone **5** (1.00 g, 1.72 mmol, 56%) as a colorless liquid.

^{1}H (400 MHz, $CDCl_3$): δ = 7.64–7.60 (m, 4H, *H*-4'), 7.44–7.33 (m, 6H, *H*-5', *H*-6'), 4.58–4.57 (m, 1H, *H*-16), 4.12–4.09 (m, 1H, *H*-3), 3.90–3.84 (m, 1H, *H*-20), 3.76–3.70 (td,

^3J = 6.93 Hz, ^2J = 9.60 Hz, 1H, *H*-15a), 3.52–3.47 (m, 1H, *H*-20), 3.41–3.35 (td, ^3J = 6.71 Hz, ^2J = 9.60 Hz 1H, *H*-15b), 2.48–2.30 (m, 2H, *H*-5), 1.87–1.79 (m, 1H, *H*-18a), 1.74–1.49 (m, 9H, *H*-2, *H*-14, *H*-17, *H*-18, *H*-19), 1.44–1.14 (m, 18H, *H*-3, *H*-6–H-12), 1.11 (s, 9H, *H*-2), 0.81 (t, ^3J = 7.46 Hz, 3H, *H*-1) ppm;

^{13}C (100 MHz, $CDCl_3$): δ = 213.1 (*C*-4'), 136.0, 135.9 (*C*-4'), 133.8, 133.3 (*C*-3'), 130.0, 130.0 (*C*-6'), 127.8, 127.8 (*C*-5'), 99.0 (*C*-16), 80.1 (*C*-3), 67.9 (*C*-15), 62.5 (*C*-20), 38.2 (*C*-5), 30.9 (*C*-17), 29.9 (*C*-14), 29.7, 29.7, 29.6, 29.6, 29.4 (*C*-7–*C*-12), 28.0 (*C*-2), 27.1 (*C*-2'), 26.4 (*C*-13), 25.7 (*C*-19), 23.1 (*C*-6), 19.9 (*C*-18), 19.5 (*C*-1'), 9.0 (*C*-1) ppm;
MS (ESI pos.): *m/z* calcd. for $C_{36}H_{56}NaO_4Si^+$ $[M+Na]^+$ 603.3840, found 603.3842, (|Δ*m/z*| = 0.32 ppm).

(*S*)-3-((*tert*-Butyldiphenylsilyl)oxy)-15-hydroxypentadecan-4-one (6)

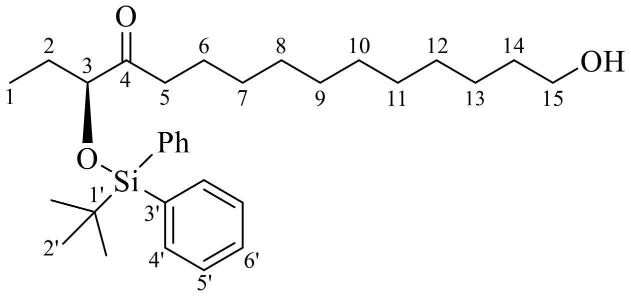

6

To a solution of acetal **5** (982 mg, 1.69 mmol, 1.00 eq.) in THF (12 mL) was added a solution of P*p*Ts (127 mg. 507 µmol, 0.30 eq.) in MeOH (4 mL). The reaction mixture was stirred for 20 h at 60 °C. The solution was concentrated in *vacuo* and a colorless solid was precipitated by addition of cyclohexane (30 mL). The solid was filtered off and the filtrate was concentrated in *vacuo*. The crude product was purified by flash column chromatography (silica gel, Cy:EtOAc, 7:3) to afford alcohol **6** (772 mg,1.55 mmol, 92%) as a colorless liquid.

^{1}H (400 MHz, CDCl$_3$): δ = 7.64–7.60 (m, 4H, *H*-4'), 7.45–7.33 (m, 6H, *H*-5', *H*-6'), 4.12–4.09 (m, 1H, *H*-3), 3.63 (t, ^{3}J = 5.78 Hz, 2H, *H*-15), 2.49–2.31 (m, 2H, *H*-5), 1.69–1.52 (m, 4H, *H*-2, *H*-14), 1.49–1.16 (m, 16H, *H*-6–H-13)), 1.11 (s, 9H, *H*-2'), 0.81 (t, ^{3}J = 5.78 Hz) ppm;

^{13}C (100 MHz, CDCl$_3$): δ = 213.1 (*C*-4), 136.0, 135.9 (*C*-4'), 133.8, 133.3 (*C*-3'), 130.0, 130.0 (*C*-6'), 127.8, 127.8 (*C*-5'), 80.1 (*C*-3), 63.2 (*C*-15), 38.1 (*C*-5), 33.0 (*C*-14), 29.7, 29.6, 3 x 29.6, 29.4 (*C*-7–*C*-12), 27.9 (*C*-2), 27.1 (*C*-2'), 25.9 (*C*-13), 23.1 (*C*-6), 19.5 (*C*-1'), 8.9 (*C*-1) ppm;

MS (ESI pos.): *m/z* calcd. for $C_{31}H_{48}NaO_3Si^+$ $[M+Na]^+$ 519.3265, found 519.3269, (|Δ*m/z*| = 0.79 ppm).

(*S*)-15-Bromo-3-((*tert*-butyldiphenylsilyl)oxy)pentadecan-4-one (7)

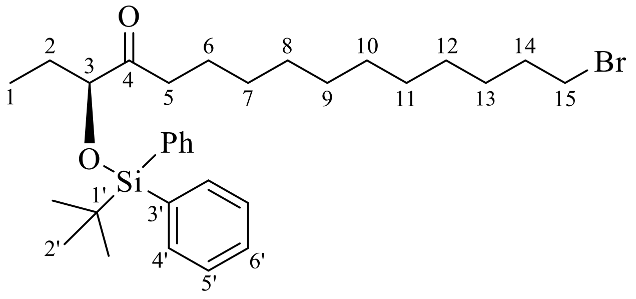

7

To a solution of alcohol **6** (772 mg, 1.55 mmol, 1.00 eq.) and CBr$_4$ (773 mg, 2.33 mmol, 1.50 eq.) in DCM (10 mL) was added PPh$_3$ (611 mg, 2.33 mmol, 1.50 eq.) in portions over 30 min. The reaction mixture was allowed to warm to room temperature over 17 h. The resulting triphenylphophane oxide was precipitated by addition of cold cyclohexane (50 mL) and filtered off. The crude product was purified by flash column chromatography (silica gel, Cy:EtOAc, 4:1) to afford bromide **7** (862 mg, 1.54 mmol, 99%) as a colorless liquid.

^{1}H (400 MHz, $CDCl_3$): δ = 7.64–7.60 (m, 4H, *H*-4'), 7.45–7.33 (m, 6H, *H*-5', *H*-6'), 4.10 (dd, ^{3}J = 6.20 Hz, ^{3}J = 5.36 Hz, 1H, *H*-3), 3.41 (t, ^{3}J = 6.88 Hz, 2H, *H*-15), 2.49–2.31 (m, 2H, *H*-5) 1.89–1.81 (m, 2H, *H*-14), 1.66–1.53 (m, 2H, *H*-2), 1.46–1.35 (m, 4H, *H*-6, *H*-13), 1.33–1.16 (m, 12H, *H*-7–H-12), 1.11 (s, 9H, *H*-2'), 0.80 (t, ^{3}J = 7.46 Hz, 3H, *H*-1) ppm;

^{13}C (100 MHz, $CDCl_3$): δ = 213.1 (*C*-4), 136.0, 135.9 (*C*-4'), 133.8, 133.3 (*C*-3'), 130.0, 130,0 (*C*-6'), 127.8, 127.8 (*C*-5'), 80.1 (*C*-3), 38.1 (*C*-5), 34.3 (*C*-15), 33.0 (*C*-14), 29.9, 29.6, 2 x 29.5, 29.4, 28.9 (*C*-7–*C*-12), 28.3 (*C*-13), 28.0 (*C*-2), 27.1 (*C*-2'), 23.1 (*C*-6), 19.5 (*C*-1'), 9.0 (*C*-1) ppm;

MS (ESI pos.): *m/z* calcd. for $C_{31}H_{47}BrNaO_2Si^+$ $[M+Na]^+$ 581.2421, found 581.2414, (|Δ*m/z*| = 1.19 ppm).

(*S*)-15-Azido-3-((*tert*-butyldiphenylsilyl)oxy)pentadecan-4-one (8)

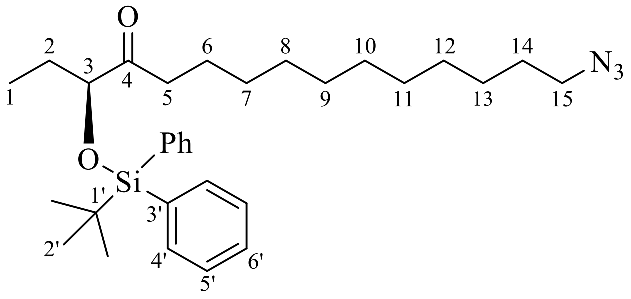

8

To a solution of bromide **7** (796 mg, 1.42 mmol, 1.00 eq.) in DMF (10 mL) was added NaN_3 (277 mg, 4.27 mmol, 3.00 eq.). The reaction mixture was heated for 16 h at 60 °C. The cooled reaction solution was mixed with water (10 mL) and EtOAc (15 mL). The aqueous phase was extracted with EtOAc (5 x 15 mL) and the combined organic extracts were washed with brine (3 x 15 mL). The organic phase was dried over $MgSO_4$ and concentrated in *vacou*. The crude product was purified by flash column chromatography (silica gel, Cy:EtOAc, 95:5) to afford azide **8** (742 mg, 1.42 mmol, quant.) as a colorless liquid.

^{1}H (400 MHz, $CDCl_3$): δ = 7.65–7.60 (m, 4H, *H*-4’), 7.45–7.39 (m, 2H, *H*-6’), 7.39–7.33 (m, 4H, *H*-5’), 4.12–4.09 (m, 1H, *H*-3), 3.25 (^{3}J = 6.98 Hz, 2H, *H*-15), 2.49–2.31 (m, 2H, *H*-5), 1.69–1.51 (m, 4H, *H*-2’, *H*-14’), 1.45–1.16 (m, 16H, *H*-6–H-13), 1.11 (s, 9H, *H*-2’), 0.80 (t, ^{3}J = 7.46 Hz, 3H, *H*-1) ppm;

^{13}C (100 MHz, $CDCl_3$): δ = 213.1 (*C*-4), 136.0, 135.9 (*C*-4’), 133.8, 133.3 (*C*-3’), 130.0, 130.0 (*C*-6’), 127.8, 127.8 (*C*-5’), 80.1 (*C*-3), 51.6 (*C*-15), 38.1 (*C*-5), 2 x 29.9, 29.6, 29.6, 29.4 (*C*-7–*C*-12), 29.0 (*C*-14), 28.0 (*C*-2), 27.1 (*C*-2’), 26.9 (*C*-13), 23.1 (*C*-6), 19.5 (*C*-1’), 8.9 (*C*-1) ppm;

MS (ESI pos.): *m/z* calcd. for $C_{31}H_{47}N_3NaO_2Si^+$ $[M+Na]^+$ 544.3330, found 544.3319, (|Δ*m/z*| = 2.00 ppm).

(*S*)-15-Azido-3-hydroxypentadecan-4-one (9, azido-(S)-LAI-1)

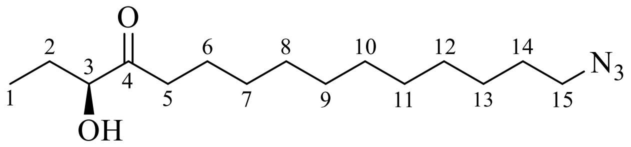

9

To a solution of silyl ether **8** (739 mg, 1.42 mmol, 1.00 eq.) in 10 mL THF at 0 °C was dropwise added TBAF (1.70 mL, 1 M in THF, 1.70 mmol, 1.20 eq.). The reaction mixture was slowly warmed to room temperature and stirred for 1.5 h. To the yellow solution was added water (10 mL) and extracted with EtOAc (4 x 15 mL). The combined organic extracts were washed with brine (15 mL), dried over $MgSO_4$ and concentrated in *vacou*. The crude product was purified by flash column chromatography (silica gel, Cy:EtOAc, 7:3) to afford alcohol **9** (363 mg, 1.28 mmol, 90%) as a colorless liquid.

^{1}H (400 MHz, CDCl$_3$): δ = 4.17–4.14 (m, 1H, *H*-3), 3.49 (d, ^{3}J = 4.90 Hz, 1H, O*H*), 3.25 (t, ^{3}J = 6.95 Hz, 2H, *H*-15), 2.51–2.38 (m, 2H, *H*-5), 1.96–1.85 (m, 1H, *H*-2$_a$), 1.65–1.54 (m, 5H, *H*-2$_b$, *H*-6, *H*-14), 1.39–1.27 (m, 14H, *H*-7–H-13), 0.94 (t, ^{3}J = 7.38 Hz, 3H, *H*-1) ppm;

^{13}C (100 MHz, CDCl$_3$): δ = 212.6 (*C*-4), 77.3 (*C*-3) 51.6 (*C*-15), 38.0 (*C*-5), 2 x 29.6, 29.5, 29.5, 29.4, 29.3 (*C*-7–*C*-12), 29.0 (*C*-14), 26.9 (*C*-2), 26.8 (*C*-13), 23.7 (*C*-6), 9.00 (*C*-1) ppm;

MS (ESI pos.): *m/z* calcd. for $C_{15}H_{29}N_3NaO_2^+$ [M+Na]$^+$ 306.2152, found 306.2168, (|Δ*m/z*| = 5.28 ppm).

(3*S*)-3-((*tert*-Butyldiphenylsilyl)oxy)-10-((tetrahydro-2*H*-pyran-2-yl)oxy)decan-4-on (11)

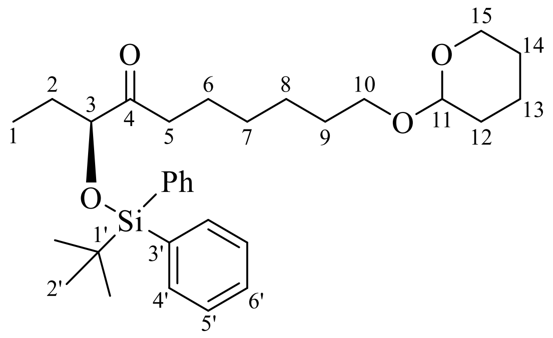

11

Magnesium turnings (10.8 g, 444 mmol, 8.00 eq.) were activated with a few beads of iodine overnight. The magnesium turnings were overlaid with THF (50 mL) and then a solution of bromide **10** (32.8 g, 124 mmol, 2.20 eq.) in THF (80 mL) was added dropwise over 30 min. The reaction mixture was then stirred for another 45 min at room temperature and finally 4 h at 55 °C. After the Grignard solution had cooled down, it was diluted with THF (100 mL) and added dropwise over 1.5 h at 0 °C to a solution of Weinreb amide **3** (21.4 g, 55.5 mmol, 1.00 eq) in THF (200 mL). The reaction mixture was thawed in an ice bath and stirred for 16 h. Under ice bath cooling, the reaction was stopped and acidified by adding of 1 M $NaHSO_4$ (150 mL) solution. The reaction mixture was stirred for another 30 min until the resulting solid

had dissolved. Ice (100 g) and cold Et_20 (500 mL) were added to the mixture. The organic phase was separated, and the aqueous phase was extracted with Et_20 (4 x 400 mL). The combined organic extracts were concentrated to approx. 1.5 L and washed with 1 M $NaHSO_4$ solution (2 x 500 mL) and brine (500 mL), dried over $MgSO_4$ and concentrated in *vacou*. The oily crude product was purified by flash column chromatography (silica gel, Cy:EtOAc, 99:1→98:2) to afford ketone **11** (25.8 g, 50.5 mmol, 91%) as a colorless liquid.

^{1}H (400 MHz, $CDCl_3$): δ = 7.64–7.60 (m, 4H, *H*-4'), 7.45–7.33 (m, 6H, *H*-5', *H*-6'), 4.58–4.56 (m, 1H, *H*-11), 4.12–4.09 (m, 1H, *H*-3), 3.89–3.84 (m, 1H, *H*-15_a), 3.71 (tt, ^3J = 6.86 Hz, ^2J = 9.60 Hz, 1H, *H*-10_a), 3.52–3.47 (m, 1H, *H*-15_b), 3.36 (tt, ^3J = 6.72 Hz, ^2J = 9.60 Hz, 1H, *H*-10_b), 2.49–2.31 (m, 2H, *H*-5), 1.86–1.78 (m, 1H, *H*-13_a), 1.75–1.47 (m, 8H, *H*-2, *H*-9, *H*-12, *H*-13_b, *H*-14), 1.11 (s, 9H, *H*-2'), 0.81 (t, ^3J = 7.48 Hz, 3H, *H*-1) ppm;

^{13}C (100 MHz, $CDCl_3$): δ = 213.0 (*C*-4), 136.0, 135.9 (*C*-4'), 133.8, 133.3 (*C*-3'), 130.0, 130.0 (*C*-6'), 127.8, 127.8 (*C*-5'), 99.0 (*C*-11), 80.1 (*C*-3), 67.7 (*C*-10), 62.5 (*C*-15), 38.1 (*C*-5), 30.9 (*C*-12), 29.7 (*C*-9), 29.2 (*C*-7), 28.0 (*C*-2), 27.1 (*C*-2'), 26.2 (*C*-8), 25.6 (*C*-14), 23.0 (*C*-6) 19.8 (*C*-13), 19.5 (*C*-1'), 9.00 (*C*-1) ppm;

MS (ESI pos.): *m/z* calcd. for $C_{31}H_{46}NaO_4Si^+$ $[M+Na]^+$ 533.3057, found 533.3047, (|Δ*m/z*| = 2.04 ppm).

(*S*)-3-((*tert*-Butyldiphenylsilyl)oxy)-10-hydroxydecan-4-on (12)

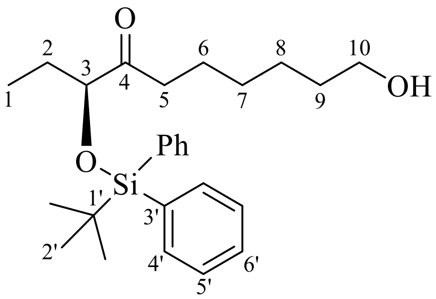

12

To a solution of alcohol **13** (19.0 g, 37.2 mmol, 1.00 eq.) in THF (360 mL) was added at room temperature a solution of P*p*Ts (2.43 g, 9.67 mmol, 0.25 eq.) in methanol (120 mL). The reaction mixture was stirred for 20 h at 60 °C. The solution was concentrated in *vacou* and a colorless solid was precipitated by addition of cold cyclohexane (100 mL). The solid was filtered off and the filtrate was concentrated in *vacou*. The crude product was purified by flash column chromatography (silica gel, Cy:EtOAc, 7:1→1:1) to afford alcohol **12** (14.5 g, 33.9 mmol, 91 %) as a colorless liquid.

^{1}H (400 MHz, CDCl$_3$): δ = 7.64–7.60 (m, 4H, *H*-4'), 7.45–7.40 (m, 2H, *H*-6'), 7.39–7.34 (m, 4H, *H*-5'), 4.13–4.10 (m, 1H, *H*-3), 3.64–3.59 (m, 2H, *H*-10), 2.50–2.32 (m, 2H, *H*-5), 1.70–1.50 (m, 4H, *H*-2, *H*-9), 1.46–1.37 (m, 2H, *H*-6), 1.36–1.15 (m, OH, *H*-7, *H*-8), 1.11 (s, 9H, *H*-2'), 0.81 (t, ^3J = 7.46 Hz, 3H, *H*-1) ppm;

^{13}C (100 MHz, CDCl$_3$): δ = 213.0 (*C*-4), 136.0, 135.0 (*C*-4'), 133.7, 133.3 (*C*-3'), 130.0, 130.0 (*C*-6'), 127.8, 127.8 (*C*-5'), 80.1 (*C*-3), 63.1 (*C*-10), 38.0 (*C*-5), 32.7 (*C*-2), 29.1 (*C*-9), 28.0 (*C*-7), 27.1 (*C*-2'), 25.7 (*C*-8), 23.0 (*C*-6), 19.5 (*C*-1'), 9.00 (*C*-1) ppm;

MS (ESI pos.): *m/z* calcd. for $C_{26}H_{38}NaO_3Si^+$ $[M+Na]^+$ 449.2482, found 449.2475, (|Δ*m/z*| = 1.67 ppm).

(*S*)-6-(2-(1-((*tert*-Butyldiphenylsilyl)oxy)propyl)-1,3-dioxolan-2-yl)hexan-1-ol (13)

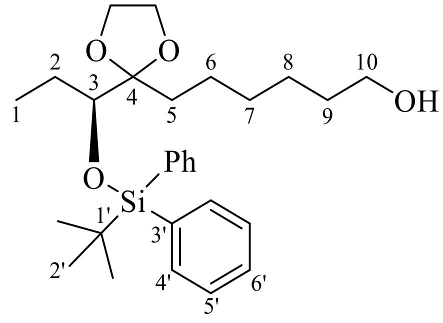

13

Ketone **12** (3.45 g, 8.09 mmol, 1.00 eq.), CSA (1.88 g, 8.09 mmol, 1.00 eq.) and 10.1 mL ethyl orthoformate (67.1 mmol, 8.30 eq.) were dissolved in dry ethylene glycol (11.0 mL, 198 mmol, 24.6 eq.) and heated for 16 h at 50 °C. The complete conversion was determined by mass spectrometry. Sat. aq. $NaHCO_3$ (50 mL) was added to the reaction mixture under ice bath cooling and then extracted with Et_20 (3 x 70 mL). The combined organic extracts were washed with brine (50 mL), dried over $MgSO_4$ and concentrated in *vacou*. The crude product was purified by flash column chromatography (silica gel, Cy:EtOAc, 5:1) to afford acetal **13** (2.98 g, 6.34 mmol, 74%) as a colorless liquid.

^{1}H (400 MHz, $CDCl_3$): δ = 7.75–7.66 (m, 4H, *H*-4’), 7.43–7.33 (m, 6H, *H*-5’, *H*-6’), 3.78–3.70 (m, 2H, *H*-1_a’’, *H*-2_a’’), 3.68–3.63 (m, 3H, *H*-1’’, *H*-10), 3.37–3.31 (m, 2H, *H*-2_b’’, *H*-3), 2.05–1.96 (m, 1H, *H*-5_a), 1.66–1.52 (m, 4H, *H*-2_a, *H*-5_b, *H*-9), 1.48–1.18 (m, 8H, OH, *H*-2_b, *H*-6, *H*-7, *H*-8) 1.05 (s, 9H, *H*-2’), 0.71 (t, ^3J = 7.48 Hz, 3H, *H*-1) ppm;

^{13}C (100 MHz, $CDCl_3$): δ = 136.6, 136.2 (*C*-4’), 135.3, 133.7 (*C*-3’), 129.5, 129.3 (*C*-6’), 127.4, 127.3 (*C*-5’), 112.5 (*C*-4), 77.6 (*C*-3), 65.6 (*C*-1’’), 64.7 (*C*-2’’), 63.2 (*C*-10), 32.9 (*C*-9), 32.1 (*C*-5), 29.9 (*C*-7), 27.3 (*C*-2’), 26.1 (*C*-2), 25.9 (*C*-8), 22.3 (*C*-6), 19.9 (*C*-1’), 11.6 (*C*-1) ppm;

MS (ESI pos.): *m/z* calcd. for $C_{28}H_{42}NaO_4Si^+$ $[M+Na]^+$ 493.2745, found 493.2740, (|Δ*m/z*| = 1.01 ppm).

(*S*)-6-(2-(1-((*tert*-Butyldiphenylsilyl)oxy)propyl)-1,3-dioxolan-2-yl)hexanoic acid (14)

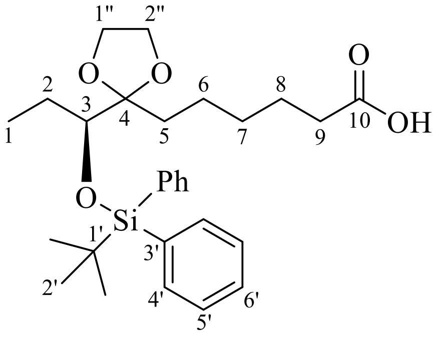

14

A solution of 10.0 g alcohol **13** (21.4 mmol, 1.00 eq.) in DMF (70 mL) was slowly added at room temperature to a solution of PDC (28.1 g, 74.7 mmol, 3.50 eq.) in DMF (200 mL). The reaction mixture was stirred for 16 h at room temperature. A brown solid was precipitated by addition of Et_20 (500 mL) under ice bath cooling and filtered off over silica gel. The filtrate was concentrated under reduced pressure. Solids were again precipitated by adding cold Et_20 (100 mL) and filtered off over silica gel. The filtrate was concentrated in vacuo for 14 h. The carboxylic acid **14** (10.4 g, 21.4 mmol, quant.) was afforded as colorless liquid and reacted without further purification.

^{1}H (400 MHz, $CDCl_3$): δ = 7.74–7.66 (m, 4H, *H*-4'), 7.43–7.33 (m, 6H, *H*-5', *H*-6'), 3.78–3.70 (m, 2H, *H*-1_a'', *H*-2_a''), 3.68–3.62 (m, 1H, *H*-1_b''), 3.37–3.31 (m, 2H, *H*-2_b'', *H*-3), 2.36 (t, ^3J = 7.54 Hz, 2H, *H*-9), 2.05–1.96 (m, 1H, *H*-5_a), 1.69–1.52 (m, 4H, *H*-2_a, *H*-5_b, *H*-8), 1.48–1.27 (m, 5H, *H*-2_b, *H*-6, *H*-7), 1.05 (s, 9H, *H*-2'), 0.71 (t, ^3J = 7.48 Hz, 3H, *H*-1)ppm;

^{13}C (100 MHz, $CDCl_3$): δ = 178.9 (*C*-10), 136.6, 136.2 (*C*-4'), 135.3, 133.7 (*C*-3'), 129.6, 129.3 (*C*-6'), 127.4, 127.3 (*C*-5'), 112.4 (*C*-4), 77.6 (*C*-3), 65.6 (*C*-1''), 64.8 (*C*-2''), 33.9

(*C*-9), 31.9 (*C*-5), 29.6 (*C*-7), 27.3 (*C*-2'), 26.1 (*C*-2), 24.9 (*C*-8), 22.0 (*C*-6), 19.8 (*C*-1'), 11.6 (*C*-1) ppm;

MS (ESI neg.): *m/z* calcd. for $C_{28}H_{39}O_5Si^-$ $[M]^-$ 483.2572, found 483.2582, ($|\Delta m/z|$ = 2.00 ppm).

(*S*)-6-(2-(1-((*tert*-Butyldiphenylsilyl)oxy)propyl)-1,3-dioxolan-2-yl)-*N*-methoxy-*N*-methylhexanamide (15)

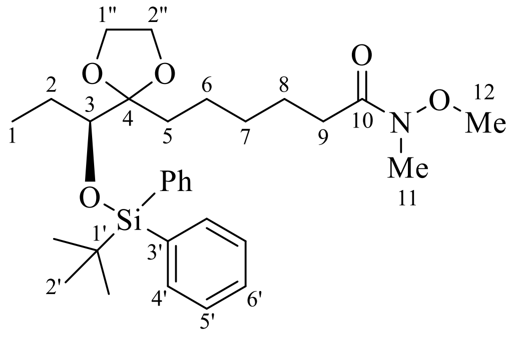

15

To a suspension of carboxylic acid **14** (10.3 g, 21.1 mmol, 1.00 eq.), *N*-methylmorpholine (2.70 mL, 24.3 mmol, 1.15 eq.) and *N,O*-dimethylhydroxylamine hydrochloride (2.37 g, 24.3 mmol, 1.15 eq.) in DCM (150 mL) was added portionwise at 0 °C EDC•HCl (4.65 g, 24.3 mmol, 1.15 eq. The reaction mixture was stirred for 1 h at 0 °C and 48 h at room temperature. The reaction was stopped by adding ice (250 g) and 1 M HCl (250 mL). The aqueous phase was extracted with DCM (4 x 350 mL), the combined organic extracts were washed with brine (300 mL), dried over $MgSO_4$ and concentrated in *vacou*. The crude product was purified by flash column chromatography (silica gel, Cy:EtOAc, 5:1) to Weinreb amide **15** (8.04 g, 15.2 mmol, 72%) as a colorless liquid.

^{1}H (400 MHz, $CDCl_3$): δ = 7.75–7.66 (m, 4H, *H*-4'), 7.42–7.33 (m, 6H, *H*-5', *H*-6'), 3.78–3.70 (m, 2H, *H*-1_a'', *H*-2_a''), 3.68 (s, 3H, *H*-12), 3.66–3.62 (m, 1H, *H*-1_b''), 3.38–3.32 (m, 2H,

H-2b'', *H*-3), 3.18 (s, 3H, *H*-11), 2.42 (t, ^{3}J = 7.52 Hz, 2H, *H*-9), 2.08–1.96 (m, 1H, *H*-5a), 1.71–1.52 (m, 4H, *H*-2a, *H*-5b, *H*-8), 1.49–1.28 (m, 5H, *H*-2b, *H*-6, *H*-7), 1.05 (s, 9H, *H*-2'), 0.71 (t, ^{3}J = 7.50 Hz, 3H, *H*-1)ppm;

^{13}C (100 MHz, CDCl$_3$): δ = 174.9 (*C*-10), 136.6, 136.2 (*C*-4'), 135.3, 133.7 (*C*-3'), 129.5, 129.3 (*C*-6'), 127.4, 127.3 (*C*-5'), 112.4 (*C*-4), 77.6 (*C*-3), 65.6 (*C*-1''), 64.8 (*C*-2''), 61.3 (*C*-12), 3 x 32.1 (*C*-5, *C*-9, *C*-12), 30.0 (*C*-7), 27.3 (*C*-2'), 26.1 (*C*-2), 24.9 (*C*-8), 22.2 (*C*-6), 19.8 (*C*-1'), 11.6 (*C*-1) ppm;

MS (ESI pos.): *m/z* calcd. for $C_{30}H_{45}NNaO_5Si^+$ $[M+Na]^+$ 550.2959, found 550.2971, (|Δ*m/z*| = 2.12 ppm).

2-((5-Bromopentyl)oxy)tetrahydro-2*H*-pyran (16)

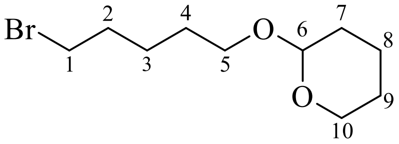

16

A solution of 5-bromopentan-1-ol (10.0 g, 55.2 mmol, 1.00 eq.) and P*p*Ts (1.39 g, 5.52 mmol, 0.10 eq.) in DCM (120 mL) was cooled to 0 °C and dihydropyran (7.56 mL, 82.2 mmol, 1.50 eq.) was added dropwise. The reaction was thawed in an ice bath in the absence of light and stirred for 18 hours. The solution was concentrated in *vacuo*. The crude product was purified by flash column chromatography (silica gel, Cy:EtOAc, 98:2) to afford acetal **16** (12.8 g, 48.3 mmol, 87 %) as a colorless liquid.

^{1}H (400 MHz, CDCl$_3$): δ = 4.58–4.56 (m, 1H, *H*-6), 3.89–3.83 (m, 1H, *H*-10a), 3.78–3.72 (m, 1-H, *H*-5a), 3.53–3.47 (m, 1H, *H*-10b), 3.44–3.36 (m, 3H, *H*-1, *H*-5b), 1.93–1.86 (m, 2H, *H*-2),

1.85–1.77 (m, 1H, *H*-8a), 1.76–1.68 (m, 1H, *H*-7a), 1.66–1.48(m, 8H, *H*-3, *H*-4, *H*-7b, *H*-8b, *H*-9) ppm;

^{13}C (100 MHz, CDCl$_3$): δ = 99.0 (1C, *C*-6), 67.4 (1C, *C*-5), 62.5 (1C, *C*-10), 33.9 (1C, *C*-1), 32.8 (1C, *C*-), 30.9 (1C, *C*-7), 29.0 (1C, *C*-4), 25.6 (1C, *C*-9), 25.6 (1C, *C*-9), 25.1 (1C, *C*-3), 19.8 (1C, *C*-8) ppm;

MS (ESI pos.): *m/z* calcd. for $C_{10}H_{19}BrNaO_2^+$ $[M+Na]^+$ 273.0461, found 273.0456, (|Δ*m/z*| = 1.89 ppm).

1-(2-((*S*)-1-((*tert*-Butyldiphenylsilyl)oxy)propyl)-1,3-dioxolan-2-yl)-11-((tetrahydro-2*H*-pyran-2-yl)oxy)undecan-6-one (17)

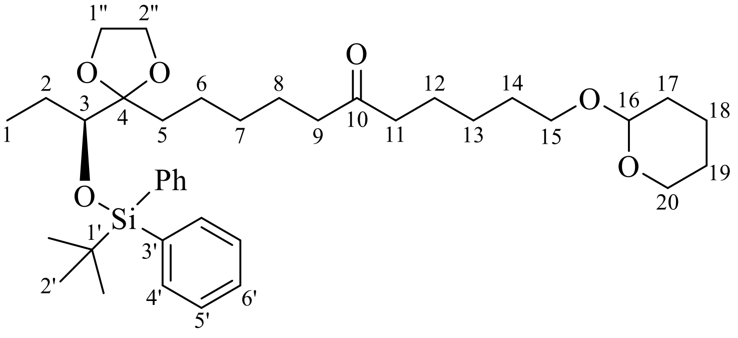

17

Magnesium turnings (807 mg, 33.2 mmol, 8.00 eq.) were activated with a bead of iodine overnight. The magnesium turnings were overlaid with THF (5 mL). A solution of bromide **16** (2.45 g, 9.75 mmol, 2.35 eq.) in THF (10 mL) was added dropwise and stirred for 45 min. The reaction was then heated at 55 °C for 4 h. The Grignard solution was cooled to room temperature and diluted with another 10 mL THF. The freshly prepared Grignard solution was added dropwise at 0 °C to a solution of Weinreb amide **17** (2.19 g, 4.15 mmol, 1.00 eq.) in THF (20 mL). The reaction was allowed to warm to room temperature for 17 h in an ice bath. Under ice bath cooling, the reaction was acidified with of 1 M HCl (20 mL) and the aqueous phase was extracted with Et_20 (5 x 50 mL), the combined organic extracts were washed with

sat. aq. $NaHCO_3$ (50 mL), brine (50 mL), dried over $MgSO_4$ and concentrated in *vacou*. The crude product was purified by flash column chromatography (silica gel, Cy:EtOAc, 9:1) to afford ketone **17** (2.43 g, 3.80 mmol, 92 %) as a colorless liquid.

^{1}H (400 MHz, $CDCl_3$): δ = 7.74–7.65 (m, 4H, *H*-4'), 7.43–7.33 (m, 6H, *H*-5', *H*-6'), 4.57–4.55 (m, 1H, *H*-16), 3.89–3.83 (m, 1H, H-20_a), 3.78–3.62 (m, 4H, *H*-1'', H-2_a'', H-15_a), 3.52–3.47 (m, 1H, H-20_b), 3.41–3.30 (m, 3H, H-2_b'', *H*-3, H-15_b), 2.42–2.37 (m, 4H, *H*-9, *H*-11), 2.02–1.94 (m, 1H, H-5_a), 1.87–1.78 (m, 1H, H-18_a), 1.74–1.68 (m, 1H, H-17_a), 1.64–1.49 (m, 12H, H-2_a, *H*-5b, *H*-8, *H*-12, *H*-14, H-17_b, H-18_b, *H*-19), 1.47–1.25 (m, 7H, H-2_b, *H*-6, *H*-7, *H*-13), 1.05 (s, 9H, *H*-2'), 0.71 (t, ^3J = 7.48 Hz, 3H, *H*-1) ppm;

^{13}C (100 MHz, $CDCl_3$): δ = 211.6 (*C*-10), 136.6, 136.2 (*C*-4'), 135.3, 133.7 (*C*-3'), 129.6, 129.3 (*C*-6'), 127.4, 127.3 (*C*-5'), 112.4 (*C*-4), 99.1 (*C*-16), 77.6 (*C*-3), 67.5 (*C*-15), 65.6 (*C*-1''), 64.8 (*C*-2''), 62.6 (*C*-20), 43.0 (*C*-9), 42.8 (*C*-11), 32.0 (*C*-5), 30.9 (*C*-17), 29.8 (*C*-7), 29.7 (*C*-14), 27.3 (*C*-2'), 26.1 (*C*-2), 26.1 (*C*-13), 25.6 (*C*-19), 24.1 (*C*-8), 23.8 (*C*-12), 22.2 (*C*-6), 19.9 (*C*-18), 19.9 (*C*-1'), 11.6 (*C*-1) ppm;

MS (ESI pos.): *m/z* calcd. for $C_{38}H_{58}NaO_6Si^+$ $[M+Na]^+$ 661.3895, found 661.3911, (|Δ*m/z*| = 2.45 ppm).

3-(5-(2-((*S*)-1-((*tert*-Butyldiphenylsilyl)oxy)propyl)-1,3-dioxolan-2-yl)pentyl)-3-(5-((tetrahydro-2*H*-pyran-2-yl)oxy)pentyl)-3*H*-diazirine (18)

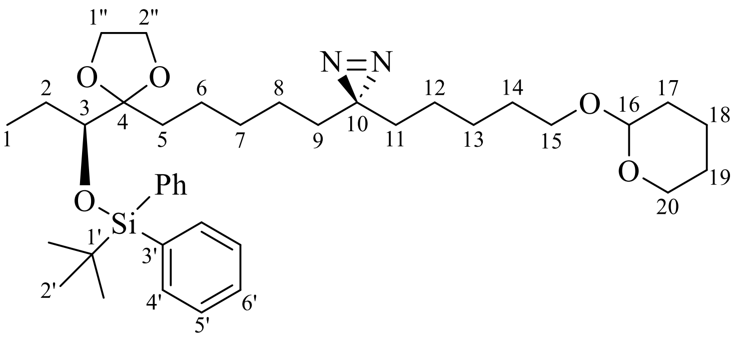

18

To a solution of ketone **17** (2.07 g ,3.24 mmol, 1.00 eq.) in methanol (3 mL) was added at 0 °C ammonia (35.0 mL, 7 M in methanol, 245 mmol, 75.0 eq.). Stirring was then continued for 3 h at 0 °C and a solution of HOSA (406 mg, 3.60 mmol, 1.15 eq.) in MeOH (30 mL) was added over 20 min. The reaction mixture was stirred for 2 d at room temperature and filtered off the resulting solid. Excess ammonia was removed in *vacuo* at 25 °C. The resulting clear colorless syrup was dissolved in methanol (30 mL) and triethylamine (879 µL, 6.25 mmol, 2.00 Äq.) was added. At 0 °C was added iodine (1.03 g 4.05 mmol, 1.25 eq.) was added in portions and the orange-brown solution was thawed over 16 h in an ice bath. Sat. aq. $Na_2S_2O_3$ (50 mL) was added, and the aqueous phase was extracted with EtOAc (3 x 50 mL). The combined organic extracts were washed with brine (50 mL), dried over $MgSO_4$ and concentrated in *vacuo*. The crude product was purified by flash column chromatography (silica gel, Cy:EtOAc, 98:2→96:2→94:2→92:8) to afford diazirine **18** (846 mg, 1.30 mmol, 42%) as a colorless liquid.

^{1}H (400 MHz, CDCl$_3$): δ = 7.74–7.65 (m, 4H, *H*-4’), 7.43–7.33 (m, 6H, *H*-5’, *H*-6’), 4.57–4.55 (m, 1H, *H*-16), 3.89–3.82 (m, 1H, *H*-20$_a$), 3.77–3.67 (m, 3H, *H*-1$_a$’’, *H*-2$_a$’’, *H*-15a), 3.66–3.60 (m, 1H, *H*-1$_b$’’), 3.52–3.47 (m, 1H, *H*-20$_b$), 3.38–3.30 (m, 3H, *H*-2$_b$’’, *H*-3, *H*-15$_b$), 1.99–1.90 (m, 1H, *H*-5$_a$), 1.88–1.78 (m, 1H, *H*-18$_a$), 1.74–1.65 (m, 1H, *H*-17$_a$), 1.61–1.26 (H-5$_b$, *H*-6, *H*-7, *H*-9, *H*-11, *H*-13, *H*-14, *H*-17$_b$, *H*-18$_b$, *H*-19), 1.15 (m, 13H, *H*-2’, *H*-8, *H*-12), 0.71 (t, ^3J = 7.48 Hz, 3H, *H*-1) ppm;

^{13}**C (100 MHz, CDCl$_3$):** δ = 136.6, 136.2 (*C*-4'), 135.3, 133.7 (*C*-3'), 129.6, 129.3 (*C*-6'), 127.4, 127.3 (*C*-5'), 112.4 (*C*-4), 99.0 (*C*-16), 77.6 (*C*-3), 67.5 (*C*-15), 65.6 (*C*-1''), 64.8 (*C*-2''), 62.5 (*C*-20), 33.1, 33.0 (*C*-9, *C*-11), 32.0 (*C*-5), 30.9 (*C*-17), 29.9 (*C*-7), 29.7 (*C*-14), 29.0 (*C*-10), 27.3 (*C*-2'), 26.1 (*C*-13), 26.1 (*C*-2), 25.6 (*C*-19), 24.1, 23.9 (*C*-8, *C*-12), 22.2 (*C*-6), 19.9 (*C*-1'), 19.8 (*C*-18), 11.6 (*C*-1) ppm;

MS (ESI pos.): *m/z* calcd. for $C_{38}H_{58}N_2NaO_5Si^+$ $[M+Na]^+$ 673.4007, found 673.3999, (|Δ*m/z*| = 1.22 ppm).

(*S*)-3-((*tert*-Butyldiphenylsilyl)oxy)-9-(3-(5-hydroxypentyl)-3*H*-diazirin-3-yl)nonan-4-one (19)

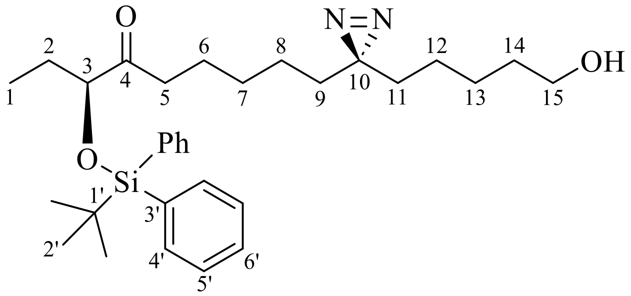

19

Reference: Sun J, Dong Y, Cao L, Wang X, Wang S, Hu Y (2004) Highly efficient chemoselective deprotection of O,O-acetals and O,O-ketals catalyzed by molecular iodine in acetone. *J Org Chem* 69: 8932-8934.

Diacetal **20** (752 mg, 1.16 mmol, 1.00 eq.) was dissolved in acetone (5.00 mL, 67.5 mmol, 58.4 eq.) and iodine (29.3 mg, 116 µmol, 0.10 eq.) was added. The reaction solution was heated at 60 °C for 10 min. EtOAc (20 mL) and brine (20 mL) were added to the cooled reaction solution. The phases were separated, and the organic phase was washed with sat. aq. $Na_2S_2O_3$ (2 x 20 mL) and brine (20 mL). The organic phase was dried over $MgSO_4$ and the concentrated in *vacou*. The intermediate was dissolved in THF (15 mL) and methanol (5 mL). To the

solution was added P*p*Ts (73.6 mg, 293 µmol, 0.25 eq.) and then heated to 55 °C for 66 h. The reaction mixture was concentrated in *vacou*. The crude product was purified by flash column chromatography (silica gel, Cy:EtOAc, 1:1) to afford alcohol **19** (540 mg, 1.16 mmol, 89%) as a colorless liquid.

^{1}H (400 MHz, $CDCl_3$): δ = 7.64–7.58 (m, 4H, H-4'), 7.46–7.33 (m, 6H, H-5'), 4.12–4.08 (m, 1H, H-3), 3.65–3.59 (m, 2H, H-15), 2.46–2.26 (m, 2H, H-5), 1.70–1.47 (m, 4H, H-2, H-14), 1.39–1.24 (m, 9H, O*H*, H-6, H-9, H-12, H-13), 1.15–0.91 (m, 15H, H-2', H-7, H-8, H-12), 0.81 (t, ^3J = 7.46 Hz) ppm;

^{13}C (100 MHz, $CDCl_3$): δ = 212.9 (C-4), 136.0 (C-4'), 135.9 (C-4'), 133.7 (C-3'), 133.3 (C-3'), 130.0 (C-6'), 130.0 (C-6'), 127.8 (C-5'), 127.8 (C-5'), 80.1 (C-3), 62.8 (C-15), 37.9 (C-5), 32.9, 32.8 (C-9, C-11), 32.6 (C-14), 28.9 (C-7), 28.7 (C-10), 27.9 (C-2), 27.1 (C-2'), 25.5 (C-13), 23.8, 23.8 (C-8, C-12), 22.8 (C-6), 19.5 (C-1'), 8.9 (C-1) ppm;

MS (ESI pos.): *m/z* calcd. for $C_{31}H_{46}N_2NaO_3Si^+$ $[M+Na]^+$ 545.3167, found 545.3171, (|Δ*m/z*| = 0.21 ppm).

(*S*)-9-(3-(5-Bromopentyl)-3*H*-diazirin-3-yl)-3-((*tert*-butyldiphenylsilyl)oxy)nonan-4-one (20)

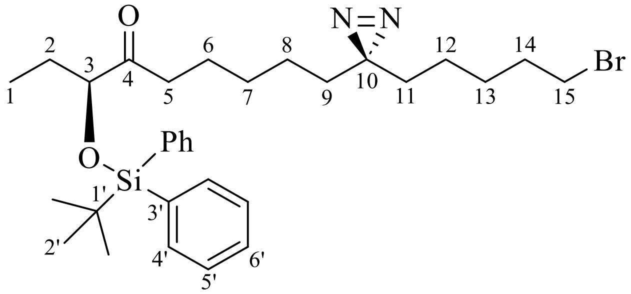

20

To a solution of alcohol **19** (540 mg, 1.03 mmol, 1.00 eq.) and CBr_4 (514 mg, 1.55 mmol, 1.50 eq.) in DCM (7 mL) and was added portionwise at 0 °C PPh_3 (406 mg, 1.55 mmol, 1.50 eq.) over 30 min and then thawed over 17 h in an ice bath. The resulting triphenylphophane oxide was precipitated by adding cold cyclohexane (50 mL) and filtered off. The crude product was purified by flash column chromatography (silica gel, Cy:EtOAc, 97:3) to afford bromide **20** (440 mg, 751 µmol, 73%) as a colorless liquid.

^{1}H (400 MHz, $CDCl_3$): δ = 7.64–7.59 (m, 4H, *H*-4'), 7.45–7.36 (m, 6H, *H*-5', *H*-6'), 4.12–4.09 (m, 1H, *H*-3), 3.38 (t, ^3J = 6.76 Hz, 2H, *H*-15), 2.46–2.28 (m, 2H, *H*-5), 1.84–1.77 (m, 2H, *H*-14), 1.69–1.50 (m, 2H, *H*-2), 1.42–1.24 (m, 8H, *H*-6, *H*-9, *H*-11, *H*-13), 1.14–0.94 (m, 15H, *H*-2', *H*-7, *H*-8, *H*-12), 0.81 (t, ^3J = 7.48 Hz, 3H, *H*-1) ppm;

^{13}C (100 MHz, $CDCl_3$): δ = 212.8 (*C*-4), 136.0, 135.9 (*C*-4'), 133.7, 133.3 (*C*-3'), 130.0, 130.0 (*C*-6'), 127.9, 127.8 (*C*-5'), 80.1 (*C*-3), 37.9 (*C*-5), 33.7 (*C*-15), 32.8, 32.8 (*C*-9, *C*-11), 32.6 (*C*-14), 28.9 (*C*-7), 28.8 (*C*-10), 28.0 (*C*-2), 27.9 (*C*-13), 27.1 (*C*-2), 23.8 (*C*-8), 32.2 (*C*-6), 19.5 (*C*-1'), 9.0 (*C*-1) ppm;

MS (ESI pos.): *m/z* calcd. for $C_{31}H_{45}BrN_2NaO_2Si^+$ $[M+Na]^+$ 607.2326, found 607.2375, (|Δ*m/z*| = 8.16 ppm).

(*S*)-9-(3-(5-Bromopentyl)-3*H*-diazirine-3-yl)-3-((*tert*-butyldiphenylsilyl)oxy)nonan-4-one (21)

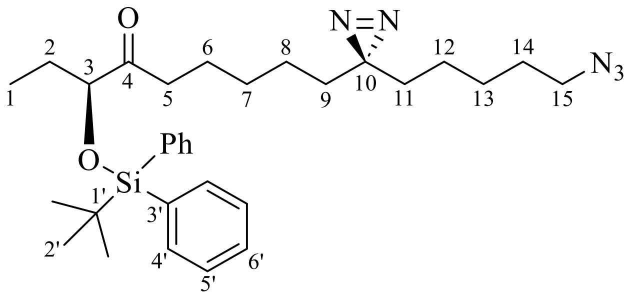

21

To a solution of bromide **20** (431 mg, 736 µmol, 1.00 eq.) in DMF (7 mL) was added NaN_3 (144 mg, 2.21 mmol, 3.00 eq.). The reaction mixture was heated for 16 h at 60 °C. The complete reaction conversion was determined by mass spectrometry. The cooled reaction solution was mixed with water (10 mL) and EtOAc (15 mL). The aqueous phase was extracted with EtOAc (5 x 15 mL) and the combined organic extracts were washed with brine (3 x 15 mL). The organic phase was dried over $MgSO_4$ and concentrated in *vacuo*. The crude product was purified by flash column chromatography (silica gel, Cy:EtOAc, 9:1) to afford azide **21** (398 mg, 727 µmol, 99 %) as a colorless liquid.

^{1}H (400 MHz, CDCl$_3$): δ =7.63–7.59 (m, 4H, *H*-4'), 7.45–7.34 (m, 6H, *H*-5', *H*-6'), 4.12–4.09 (m, 1H, *H*-3), 3.25 (t, ^3J = 6.88 Hz, 2H, *H*-15), 2.46–2.28 (m, 2H, *H*-5), 1.67–1.51 (m, 4H, *H*-2, *H*-14), 1.38–1.28 (m, 8H, *H*-6, *H*-9, *H*-11, *H*-13), 1.14–0.94 (m, 15H, *C*-2', *C*-7, *C*-8, *C*-12), 0.81 (t, ^3J = 7.46 Hz, 3H, *H*-1) ppm;

^{13}C (100 MHz, CDCl$_3$): δ = 212.8 (C-4), 136.0 (C-4'), 135.9 (C-4'), 133.7 (C-3'), 133.29 (C-3'), 130.0 (C-6'), 130.0 (C-6'), 127.8 (C-5'), 127.8 (C-5'), 80.1 (C-3), 51.4 (C-15), 37.9 (C-5), 32.9, 32.8 (C-9, C-11), 28.9 (C-7), 28.8 (C-14), 28.8 (C-10), 28.0 (C-2), 27.1 (C-2'), 26.5 (C-13), 23.8 (C-8), 23.6 (C-12), 22.8 (C-6), 19.5 (C-1'), 8.9 (C-1) ppm;

MS (ESI pos.): *m/z* calcd. for $C_{31}H_{45}N_5NaO_2Si^+$ $[M+Na]^+$ 570.3235, found 570.3224, (|Δ*m/z*| = 1.95 ppm).

(*S*)-9-(3-(5-Bromopentyl)-3*H*-diazirine-3-yl)-3-((*tert*-butyldiphenylsilyl)oxy)nonan-4-one (22)

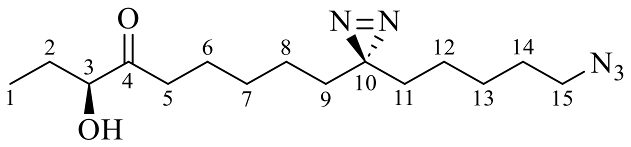

22

A solution of silyl ether **23** (398 mg, 727 µmol, 1.00 eq.) in THF (5 mL) was cooled to 0 °C and TBAF (872 µL, 1 M in THF, 872 µmol, 1.20 eq.) was added dropwise, the reaction was slowly warmed to room temperature and stirred for 1.5 h. The yellow reaction solution was mixed with aq. sat. NH_4Cl (10 mL) under ice bath cooling and extracted with EtOAc (4 x 15 mL). The combined organic phases were washed with brine (15 mL), dried over $MgSO_4$ and concentrated in *vacuo*. The crude product was purified by flash column chromatography (silica gel, Cy:EtOAc, 9:1) to afford alcohol **22** (190 mg, 615 µmol, 85%) as a colorless liquid.

^{1}H (600 MHz, $CDCl_3$): δ = 4.15–4.13 (m, 1H, *H*-3), 3.45 (bs, 1H, OH), 3.24 (t, ^3J = 6.88 Hz, 2H, *H*-15), 2.50–2.36 (m, 2H, *H*-5), 1.94–1.84 (m, 1H, *H*-2_a), 1.64–1.51 (m, 5H, *H*-2_b, *H*-6, *H*-14), 1.41–1.35 (m, 4H, *H*-9, *H*-11), 1.33–1.20 (m, 4H, *H*-7, *H*-13), 1.13–1.03 (m, 4H, *H*-8, *H*-12), 0.93 (t, ^3J = 7.40 Hz, 3H, *H*-1) ppm;

^{13}C (150 MHz, $CDCl_3$): δ = 212.3 (*C*-4), 77.3 (*C*-3), 51.4 (*C*-15), 37.7 (*C*-5), 32.9 (*C*-11), 32.7 (*C*-9), 28.9 (*C*-7), 28.8 (*C*-14), 28.7 (*C*-10), 26.9 (*C*-2), 26.4 (*C*-13), 23.8 (*C*-8), 23.6 (*C*-12), 23.4 (*C*-6), 9.00 (*C*-1) ppm;

MS (ESI pos.): *m/z* calcd. for $C_{15}H_{27}N_5NaO_2^+$ $[M+Na]^+$ 332.2057, found 332.2052, (|Δ*m/z*| = 1.55 ppm).

NMR-Spectra

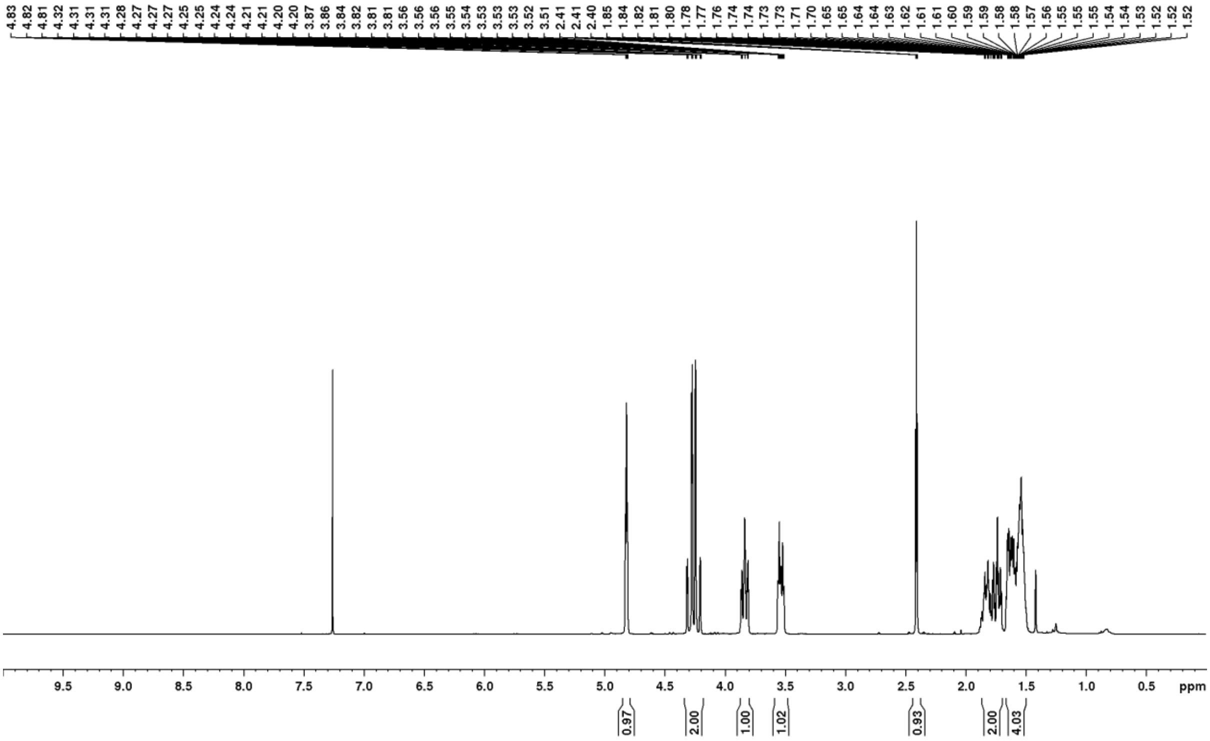

Figure 1: ^{1}H-NMR spectrum (400 MHz, $CDCl_3$) of compound **2**.

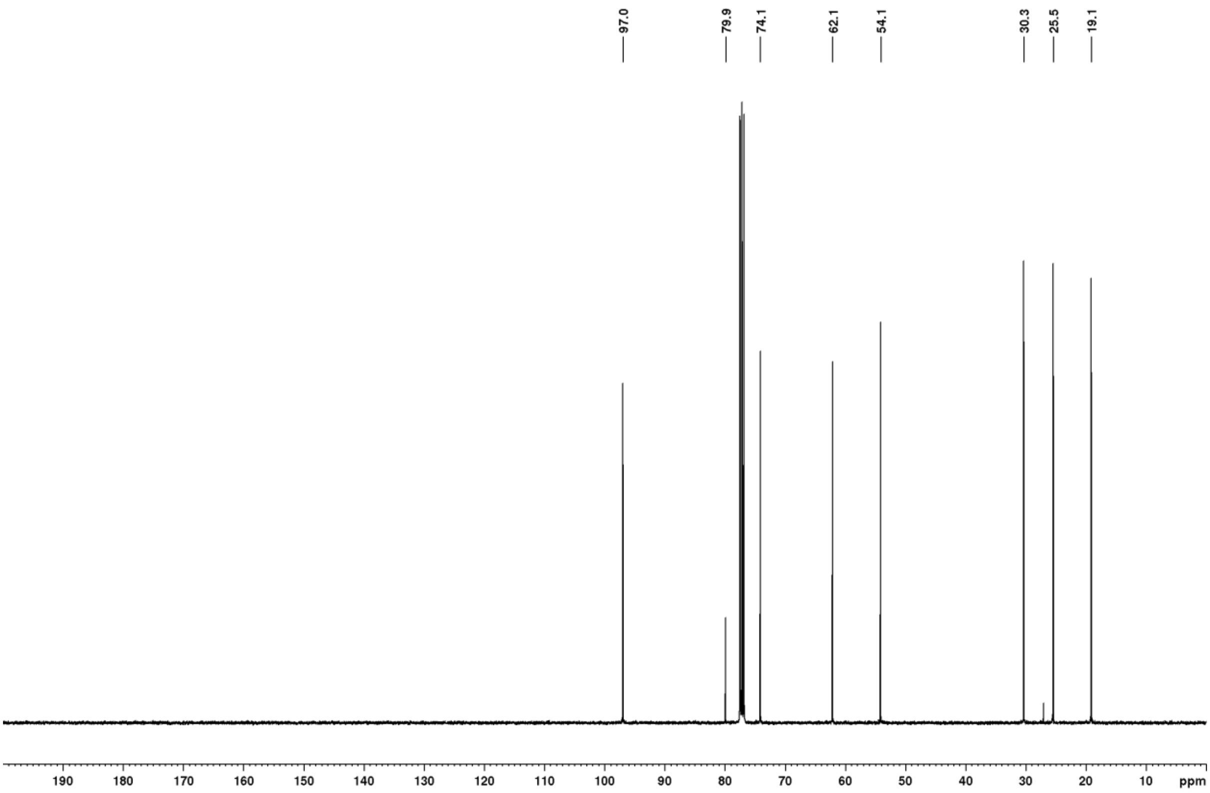

Figure 2: ^{13}C-NMR spectrum (100 MHz, $CDCl_3$) of compound **2**.

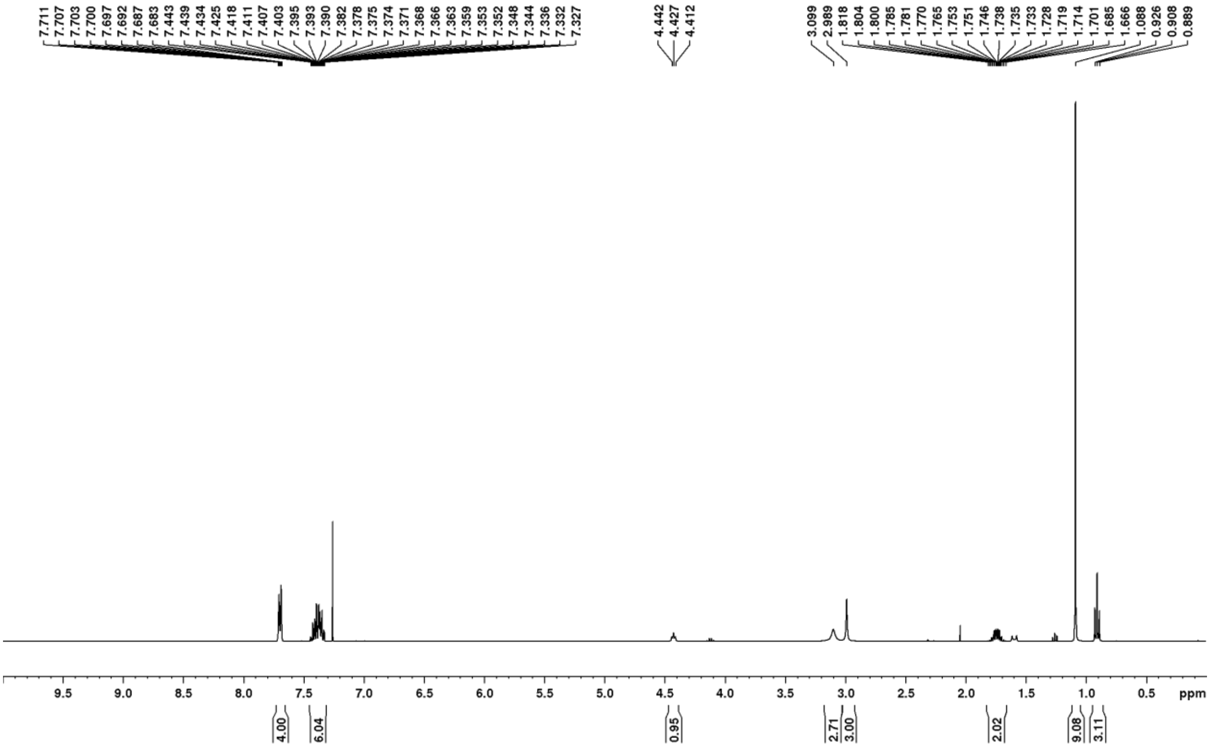

Figure 3: ^{1}H-NMR spectrum (400 MHz, $CDCl_3$) of compound **3**.

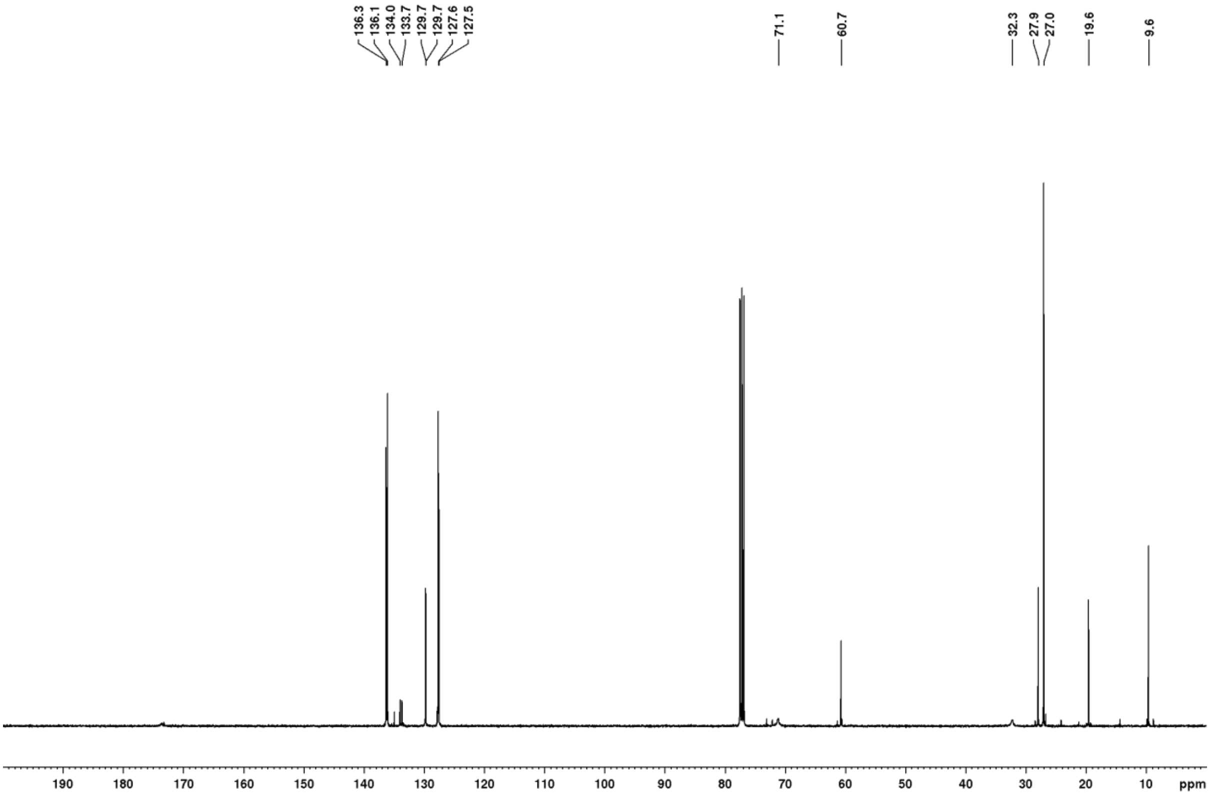

Figure 4: ^{13}C-NMR spectrum (100 MHz, $CDCl_3$) of compound **3**.

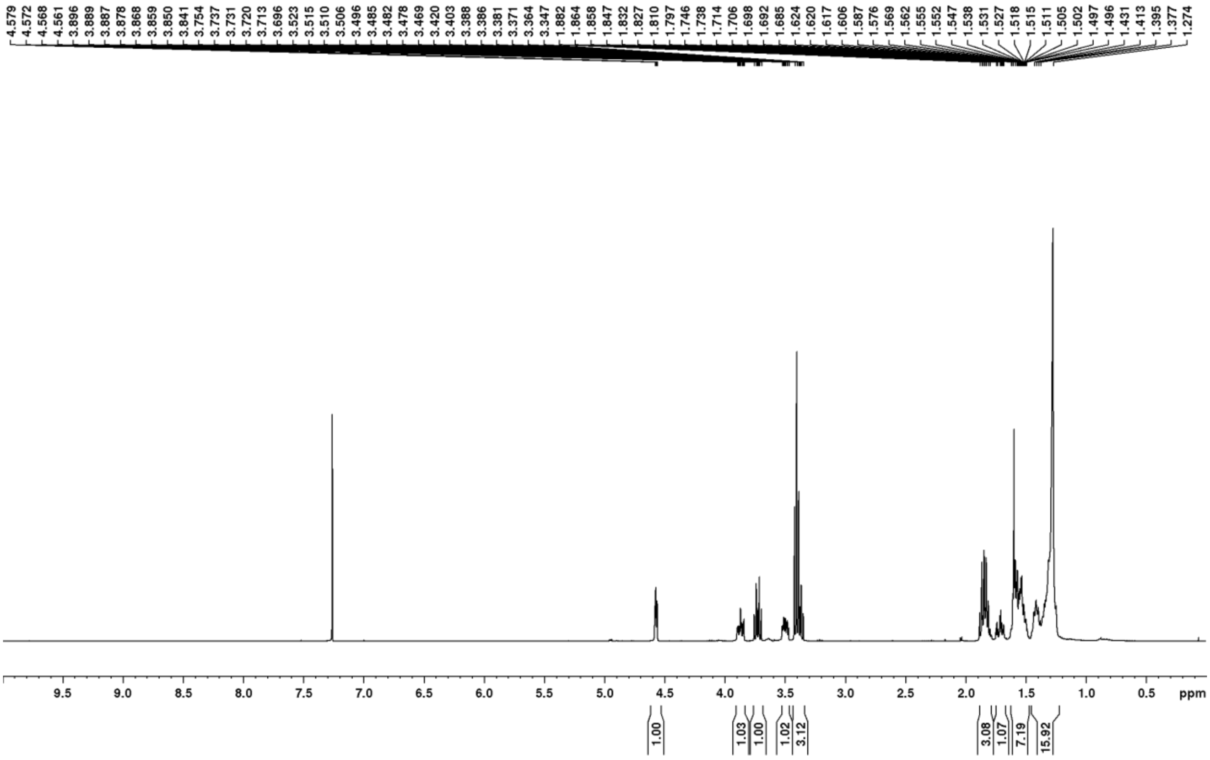

Figure 5: ^{1}H-NMR spectrum (400 MHz, $CDCl_3$) of compound **4**.

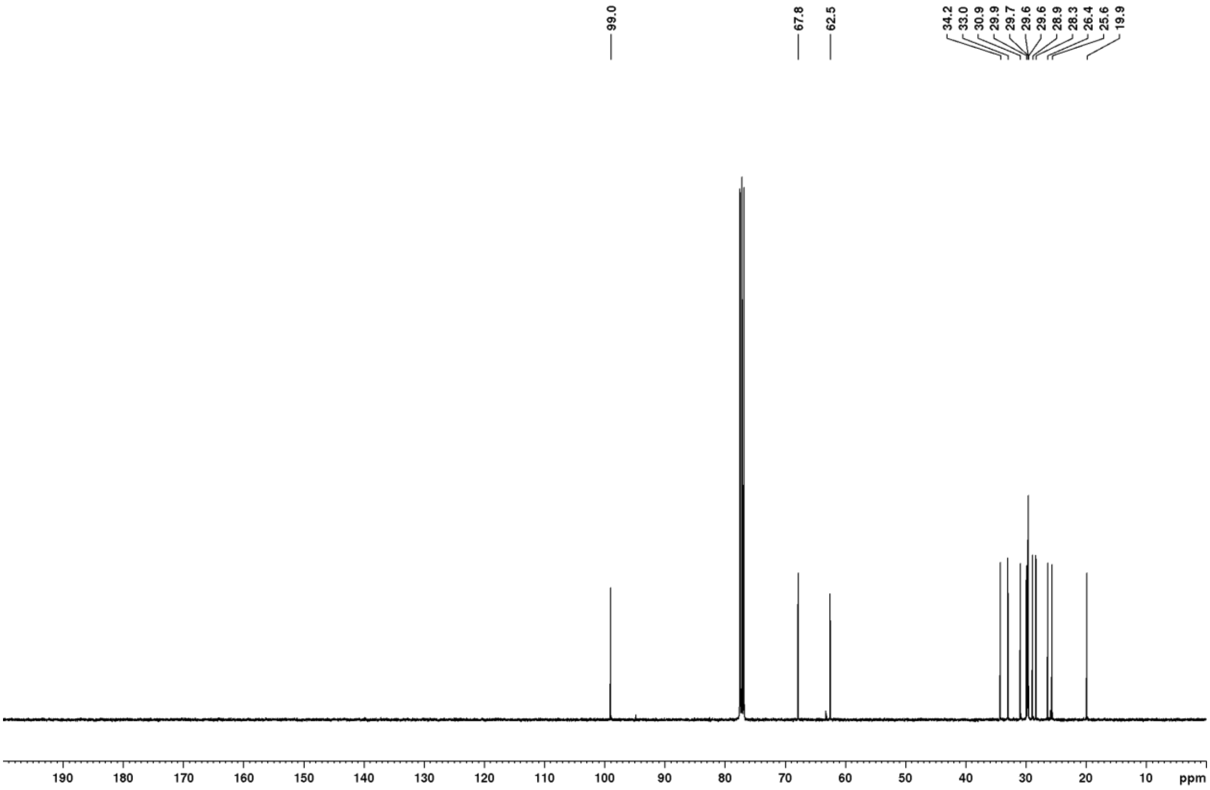

Figure 6: ^{13}C-NMR spectrum (100 MHz, $CDCl_3$) of compound **4**.

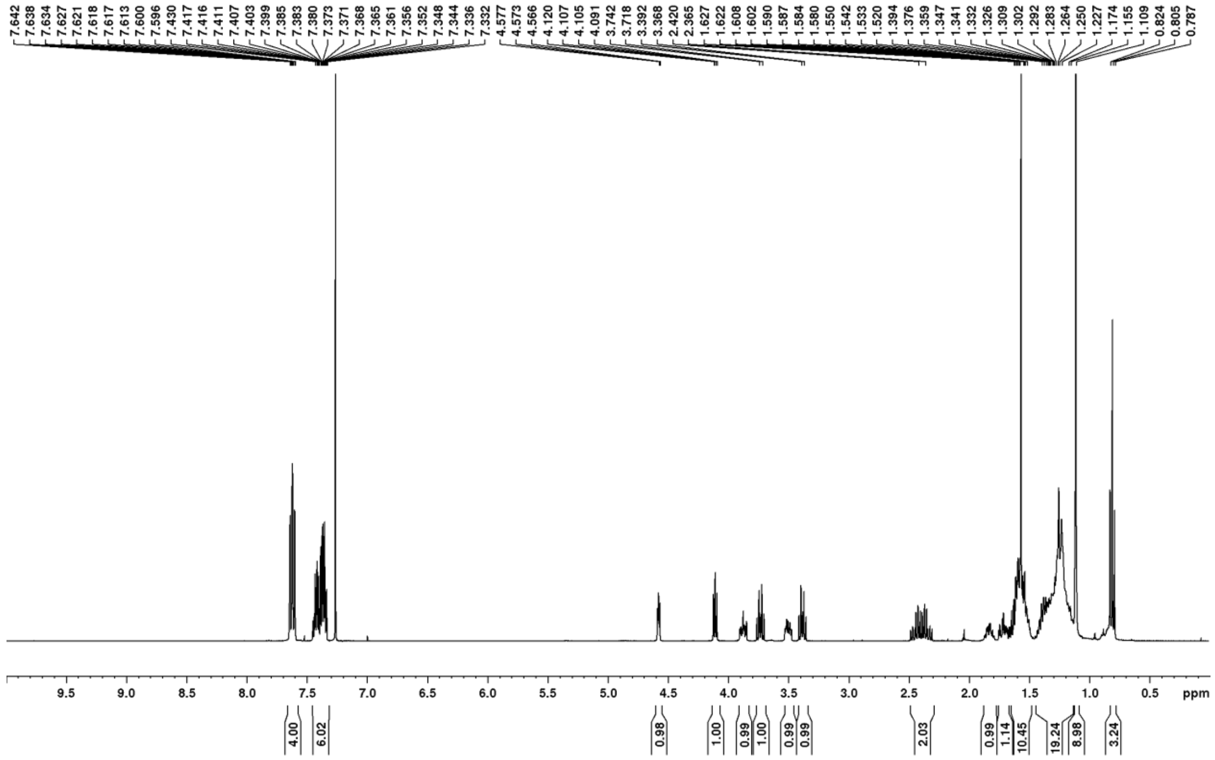

Figure 7: ^{1}H-NMR spectrum (400 MHz, $CDCl_3$) of compound **5**.

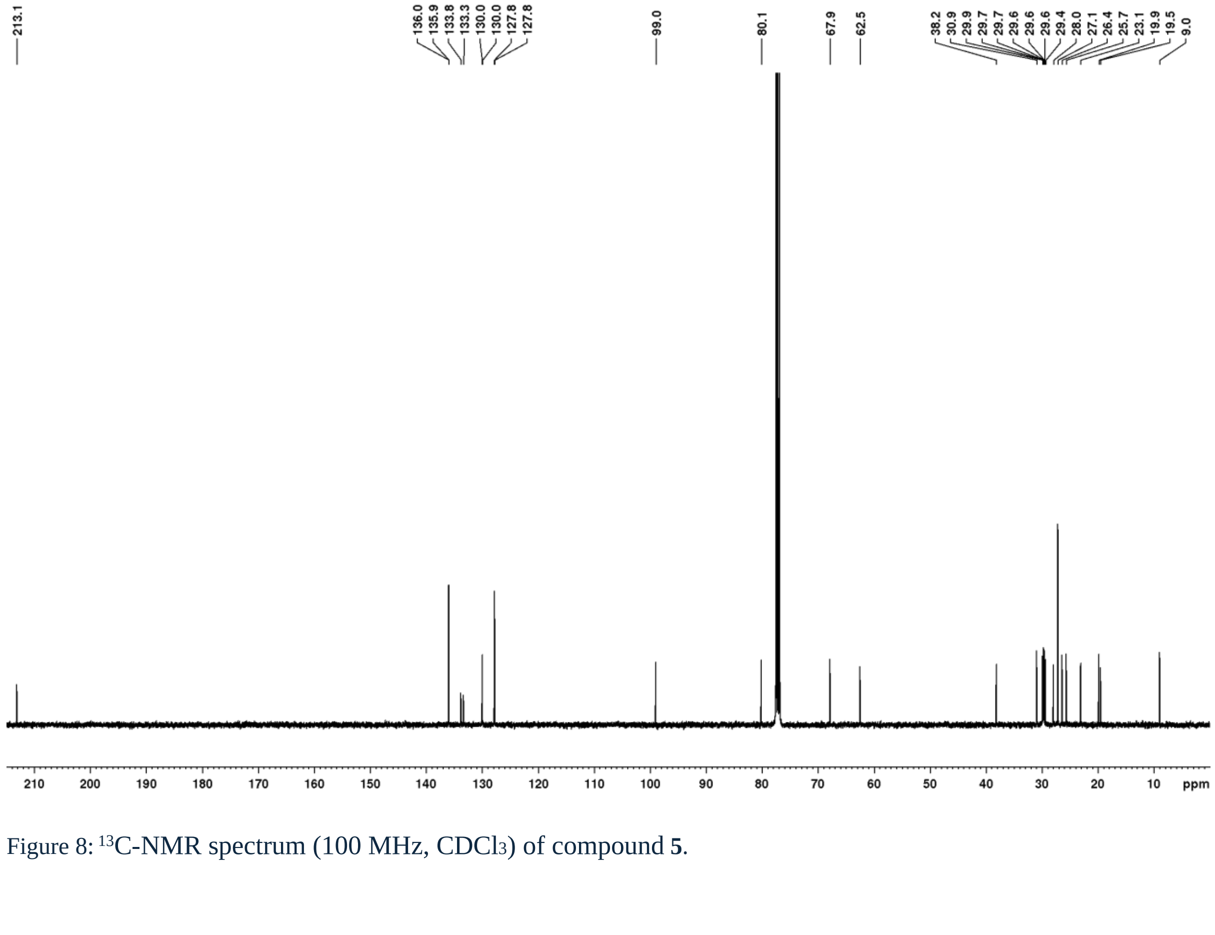

Figure 8: ^{13}C-NMR spectrum (100 MHz, $CDCl_3$) of compound **5**.

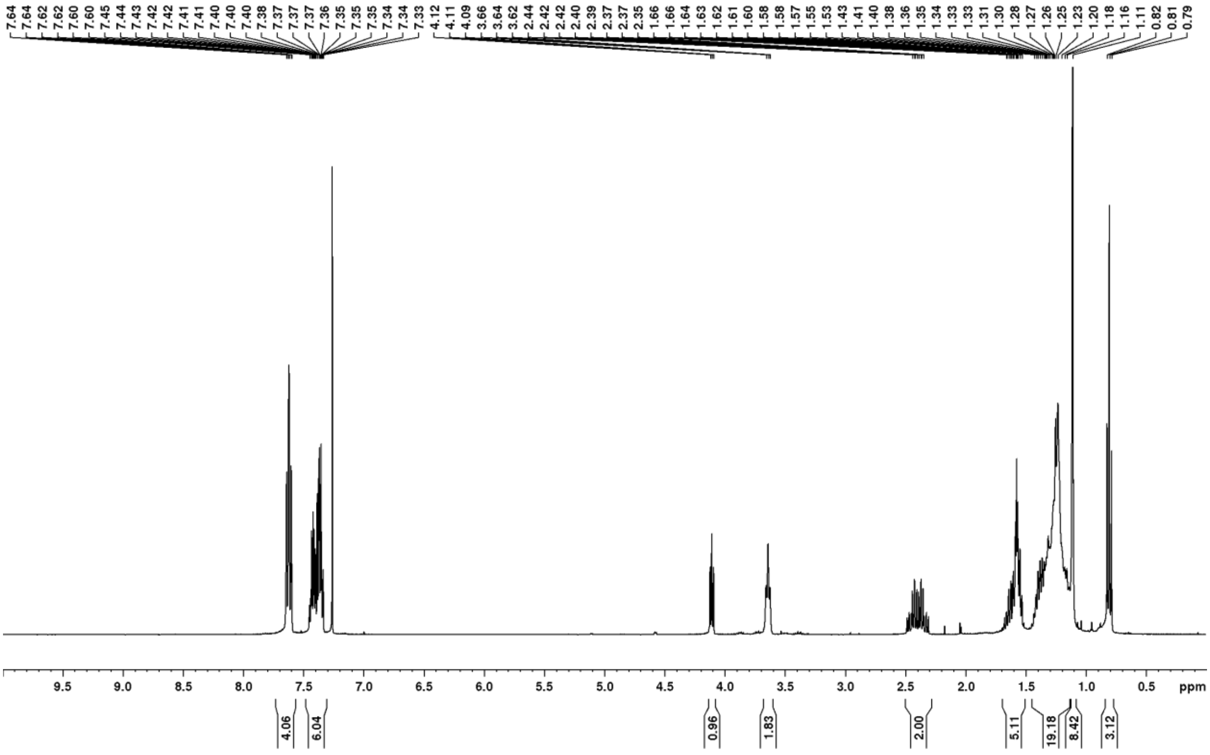

Figure 9: ^{1}H-NMR spectrum (400 MHz, $CDCl_3$) of compound **6**.

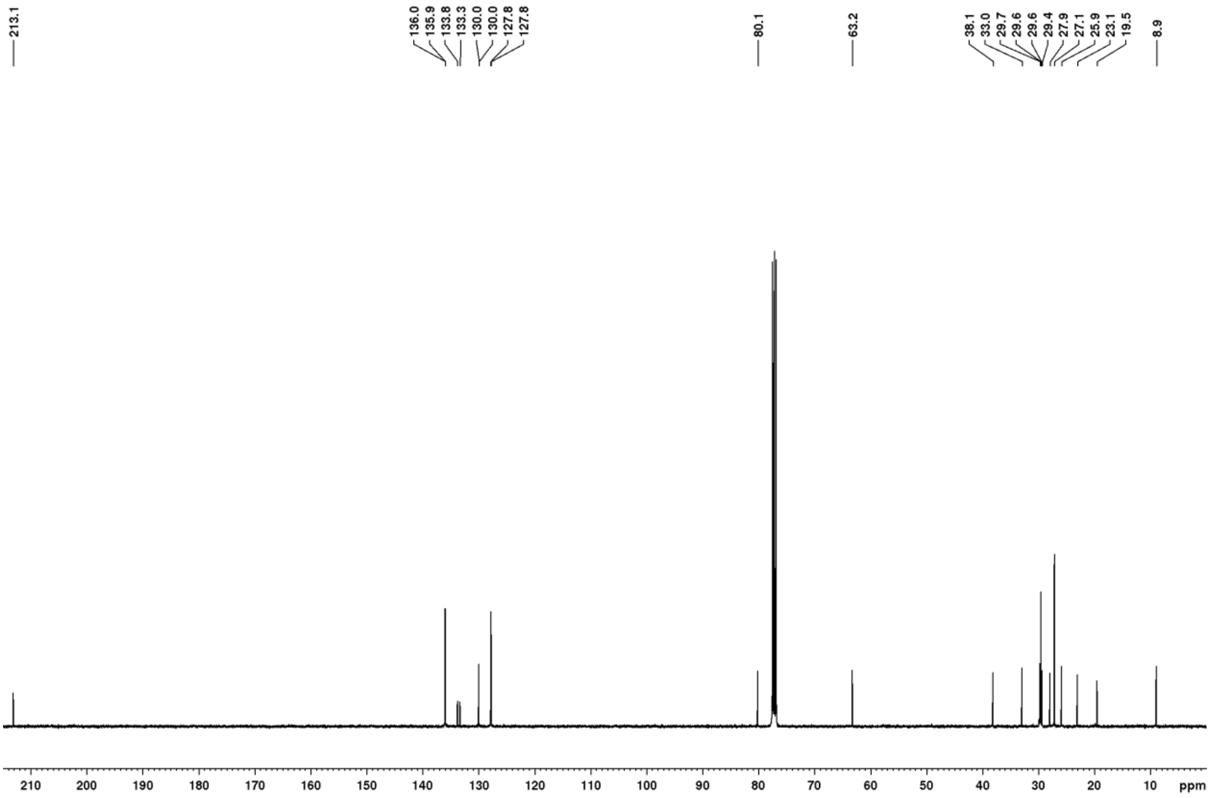

Figure 10: ^{13}C-NMR spectrum (100 MHz, $CDCl_3$) of compound **6**.

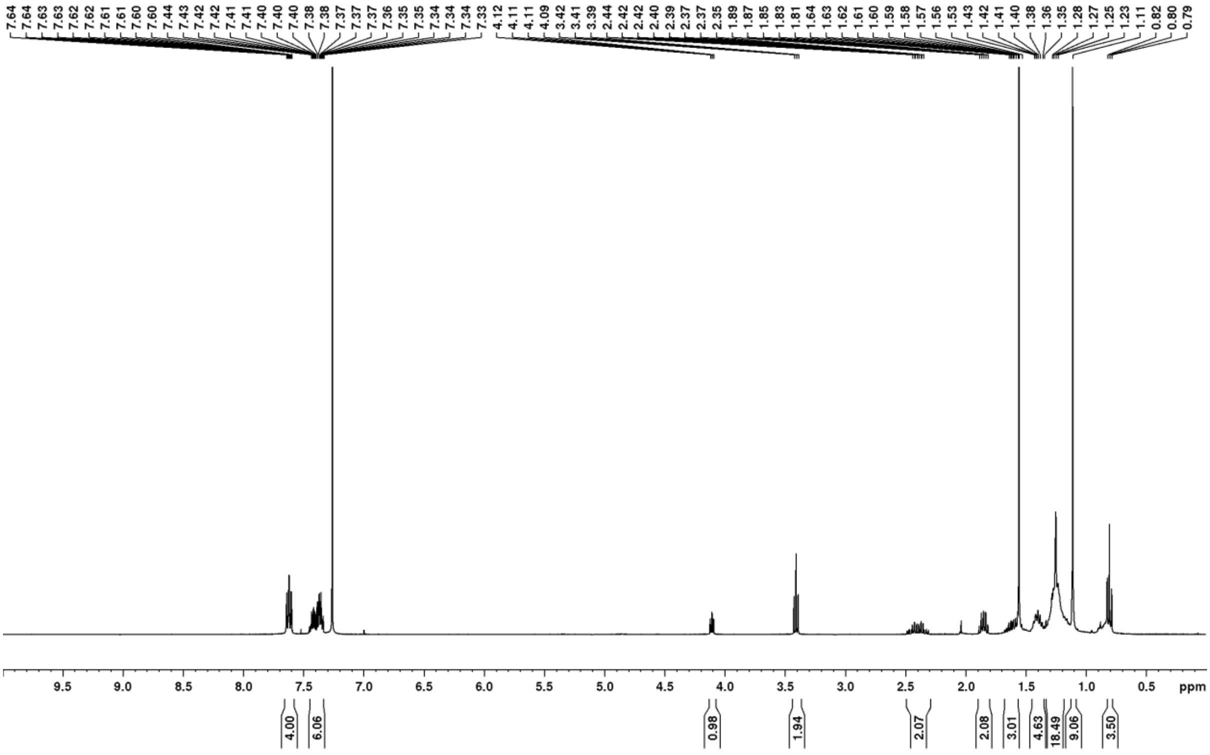

Figure 11: ^{1}H-NMR spectrum (400 MHz, $CDCl_3$) of compound **7**.

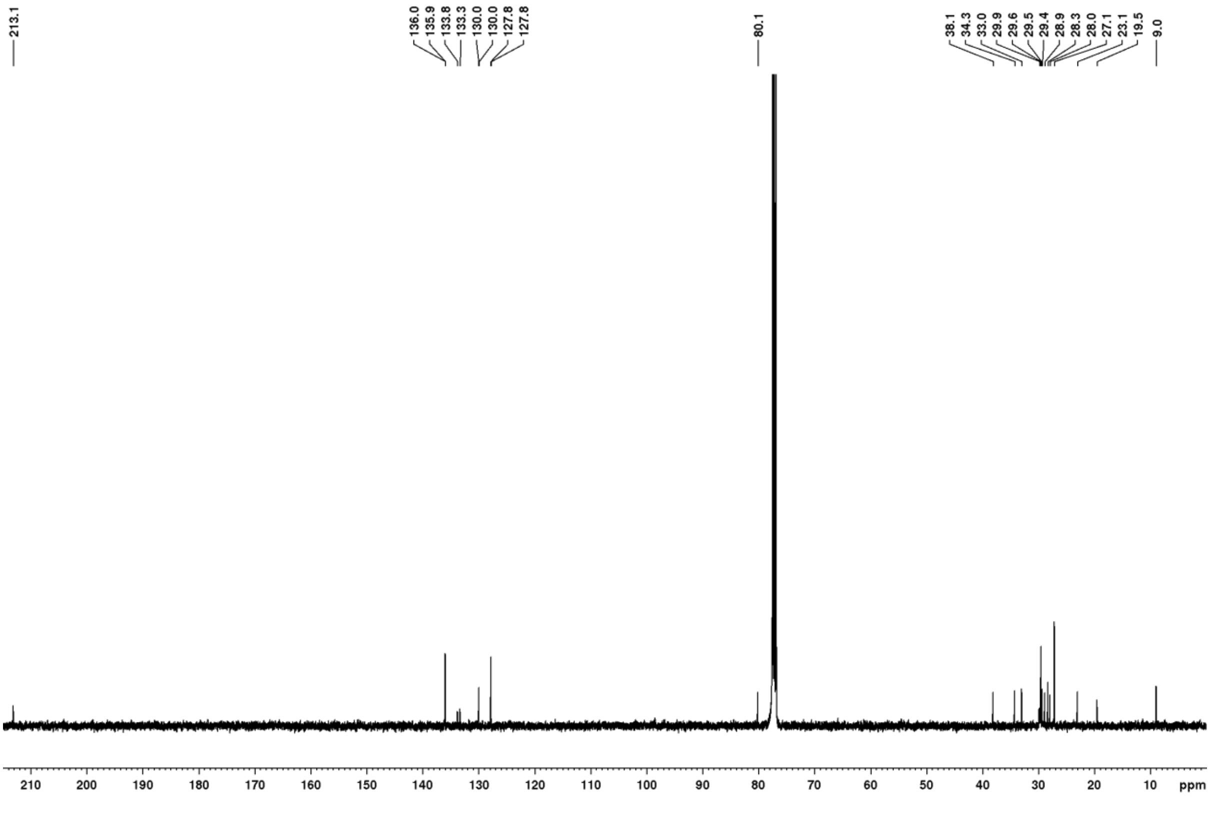

Figure 12: ^{13}C-NMR spectrum (100 MHz, $CDCl_3$) of compound **7**.

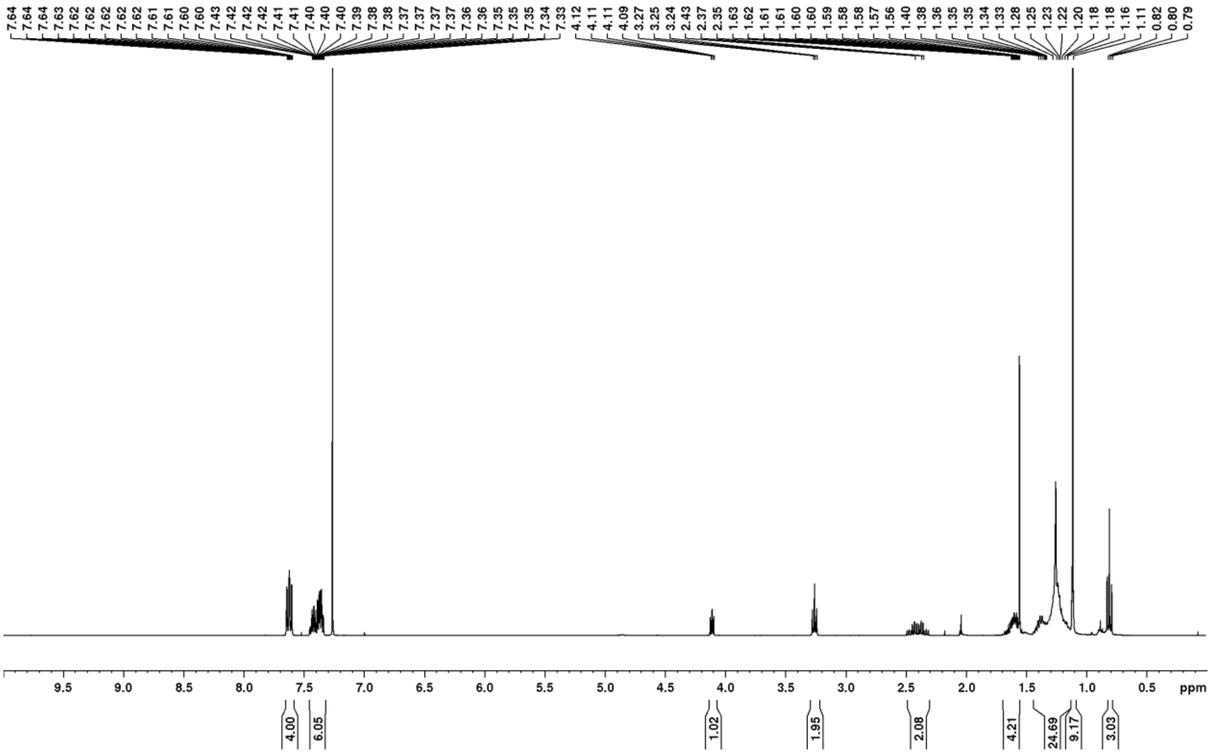

Figure 13: ^{1}H-NMR spectrum (400 MHz, $CDCl_3$) of compound **8**.

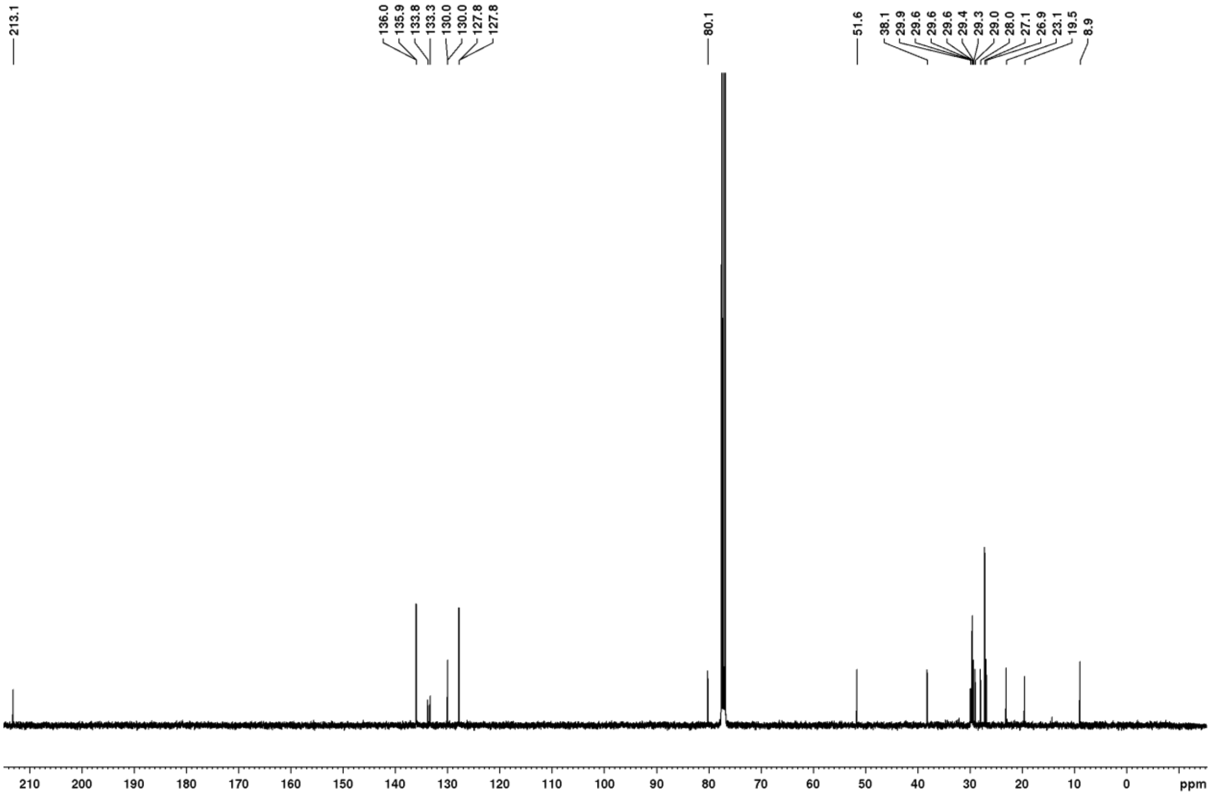

Figure 14: ^{13}C-NMR spectrum (100 MHz, $CDCl_3$) of compound **8**.

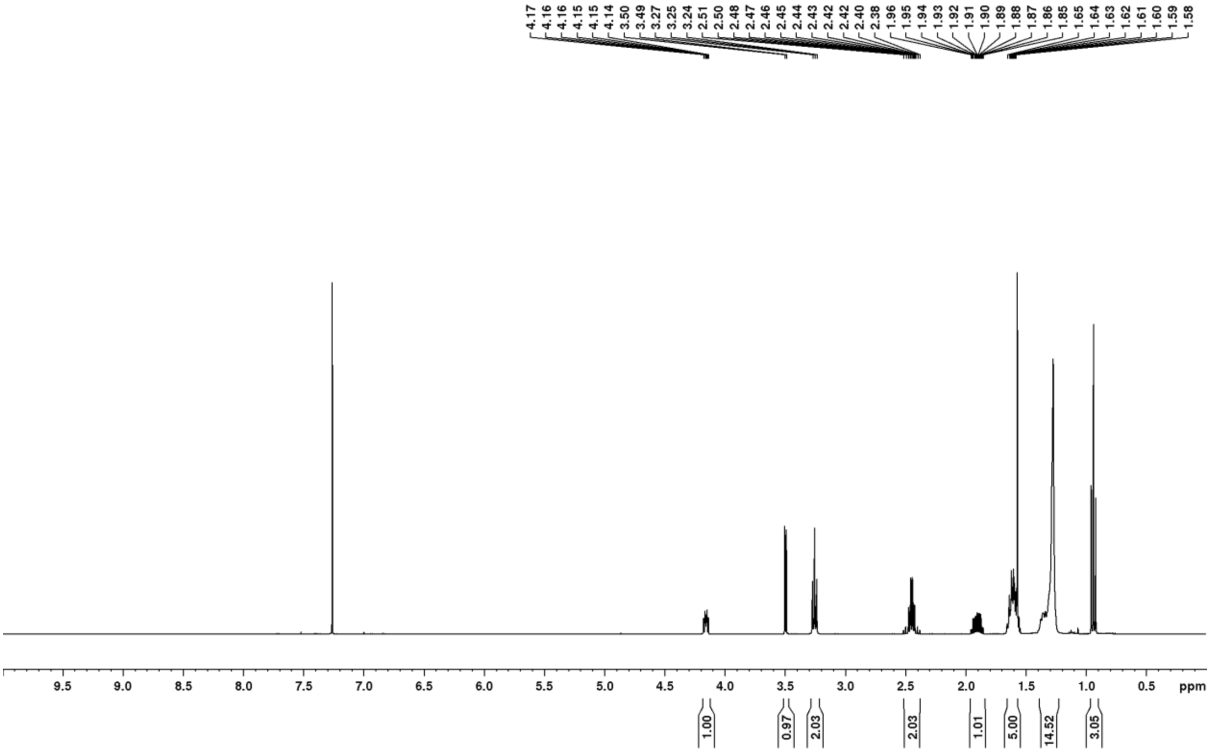

Figure 15: ^{1}H-NMR spectrum (400 MHz, $CDCl_3$) of compound **9 (**azido-(*S*)-LAI-1).

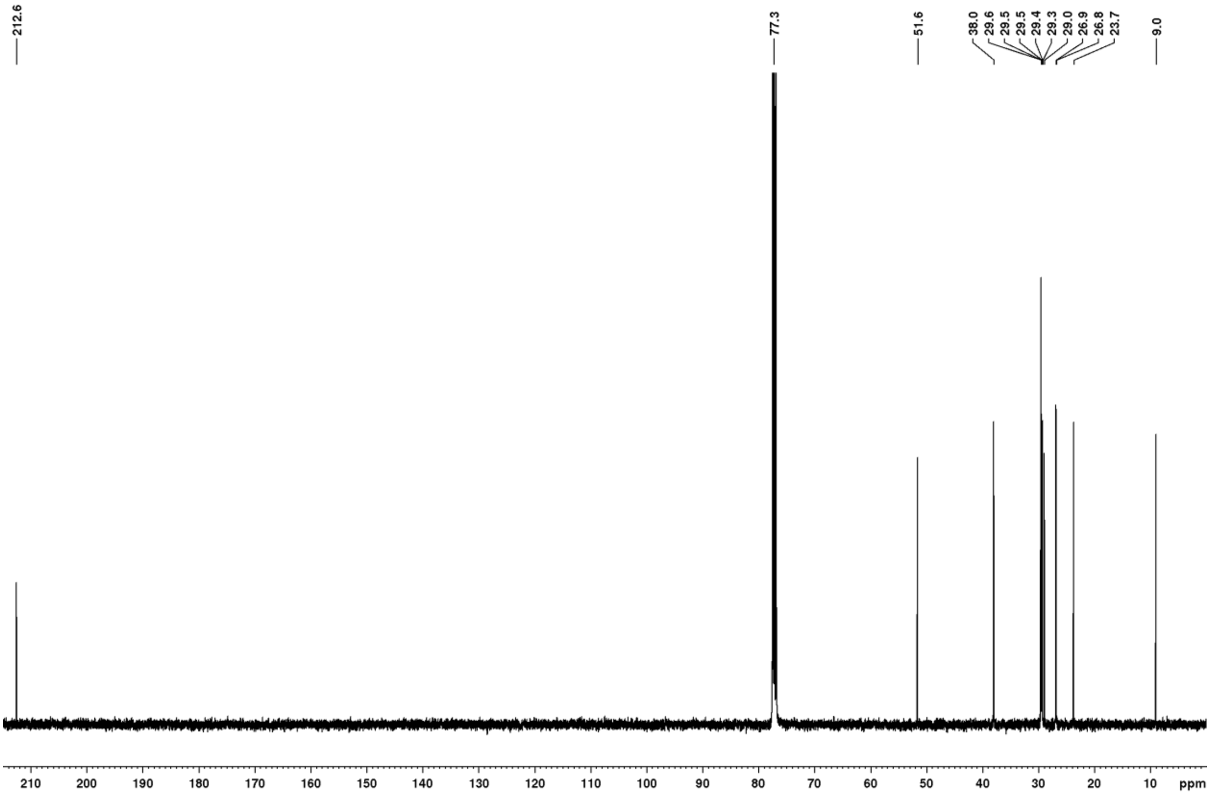

Figure 16: ^{13}C-NMR spectrum (100 MHz, $CDCl_3$) of compound **9** (azido-(*S*)-LAI-1).

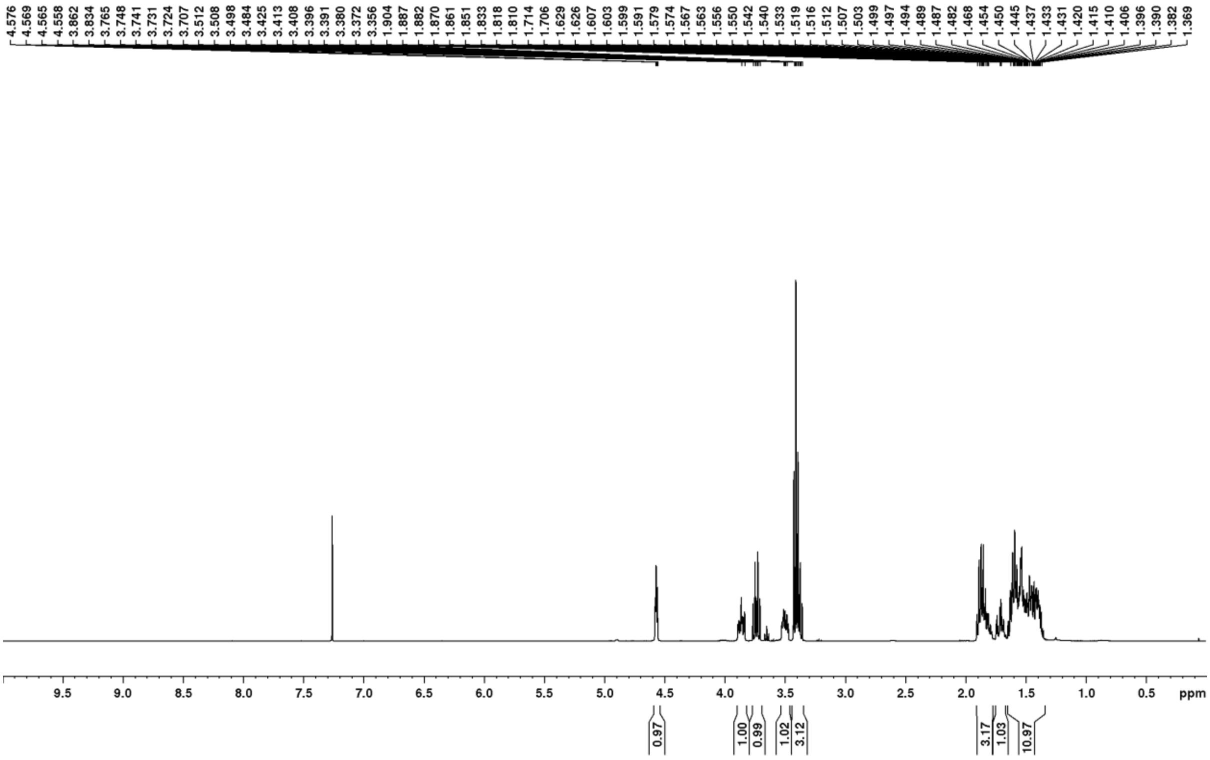

Figure 17: ^{1}H-NMR spectrum (400 MHz, $CDCl_3$) of compound **10**.

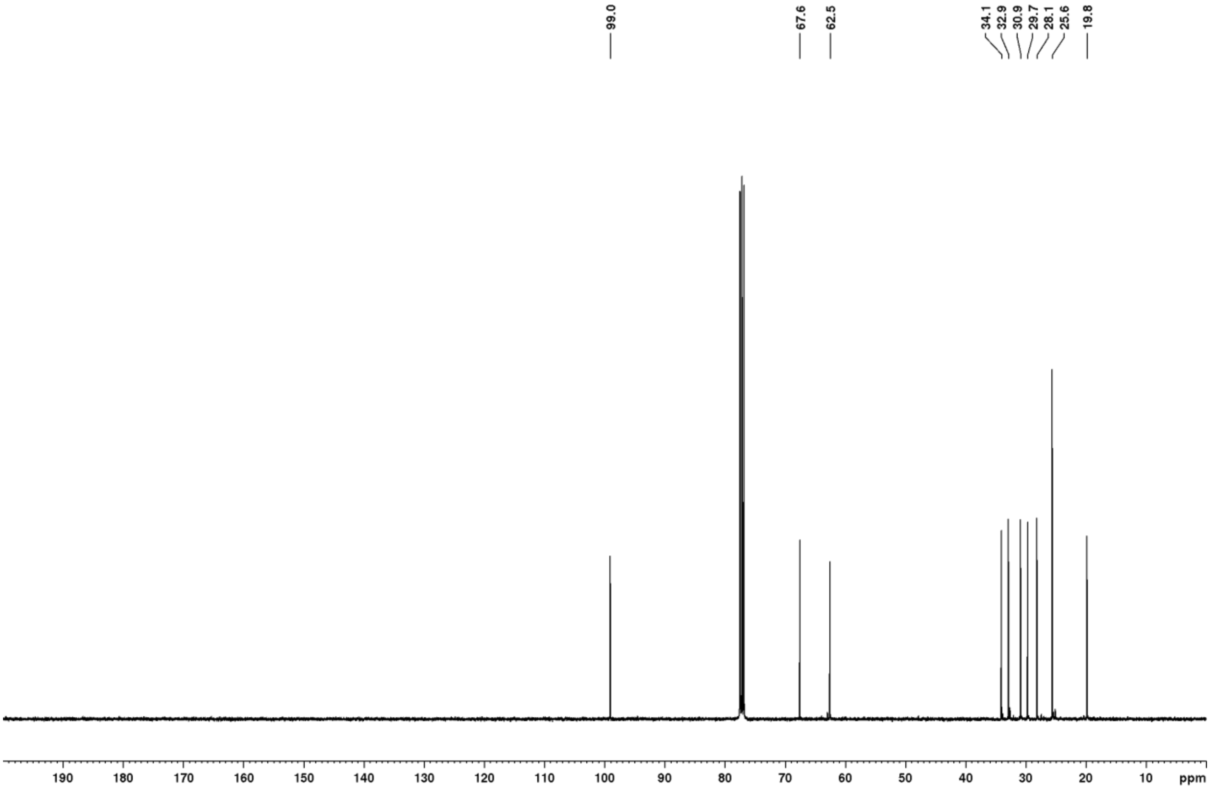

Figure 18: ^{13}C-NMR spectrum (100 MHz, $CDCl_3$) of compound **10**.

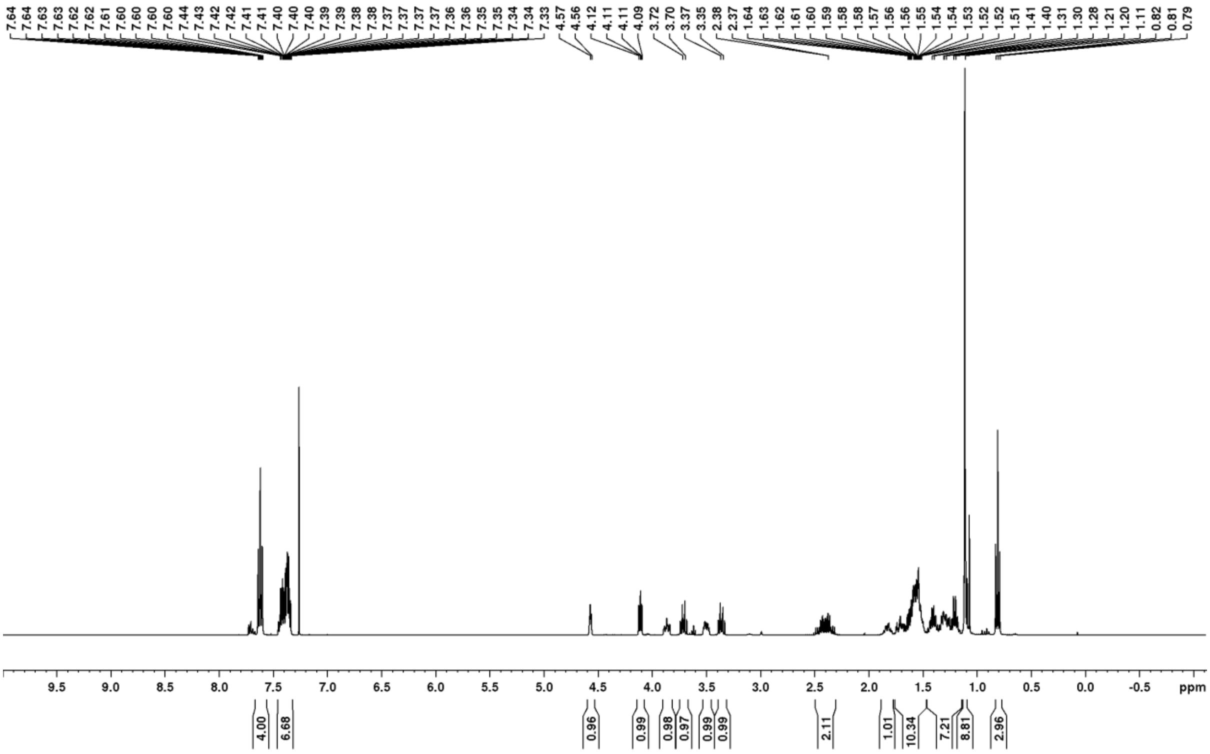

Figure 19: ^{1}H-NMR spectrum (400 MHz, $CDCl_3$) of compound **11**.

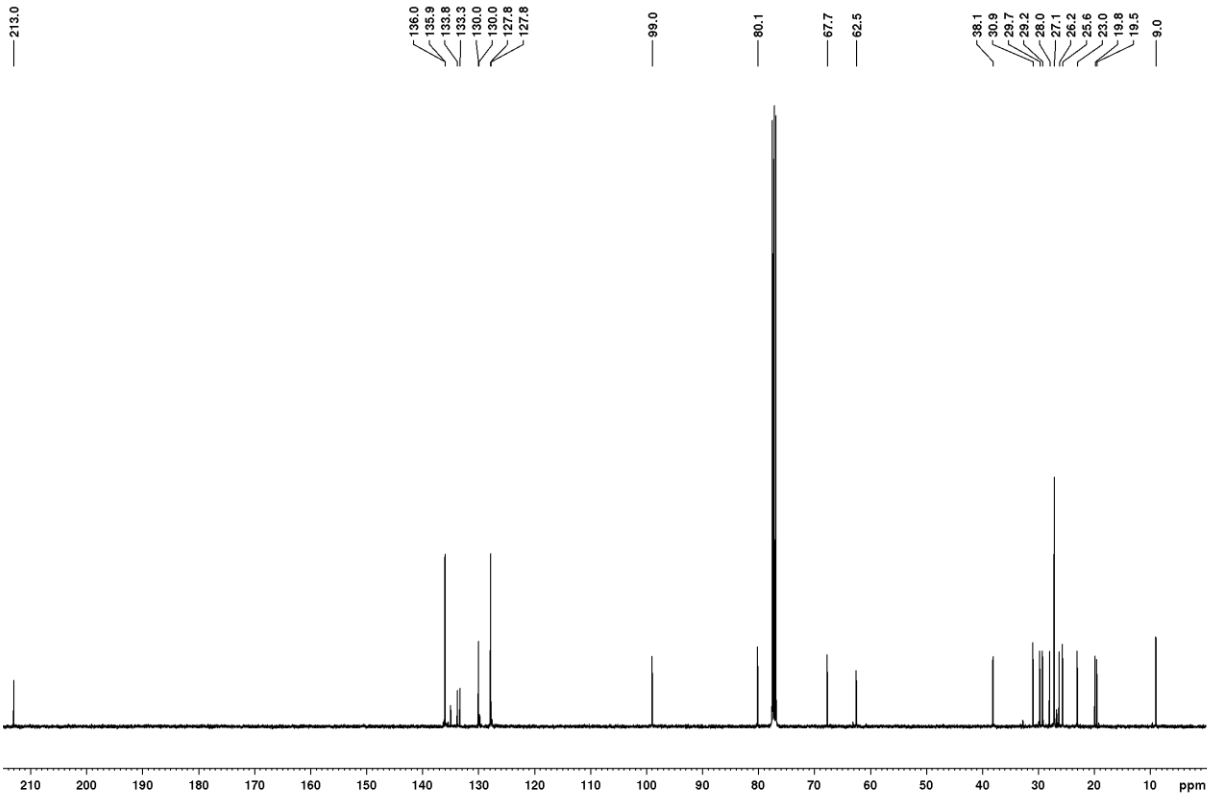

Figure 20: ^{13}C-NMR spectrum (100 MHz, $CDCl_3$) of compound **11**.

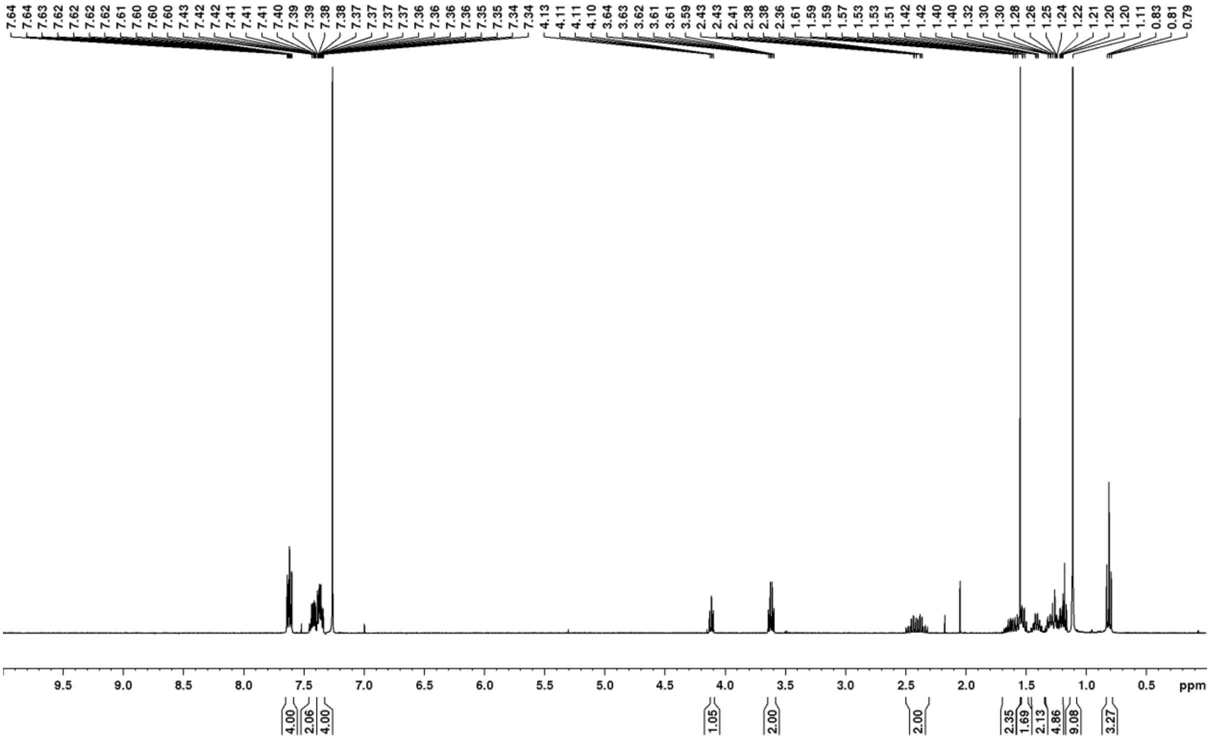

Figure 21: ^{1}H-NMR spectrum (400 MHz, $CDCl_3$) of compound **12**.

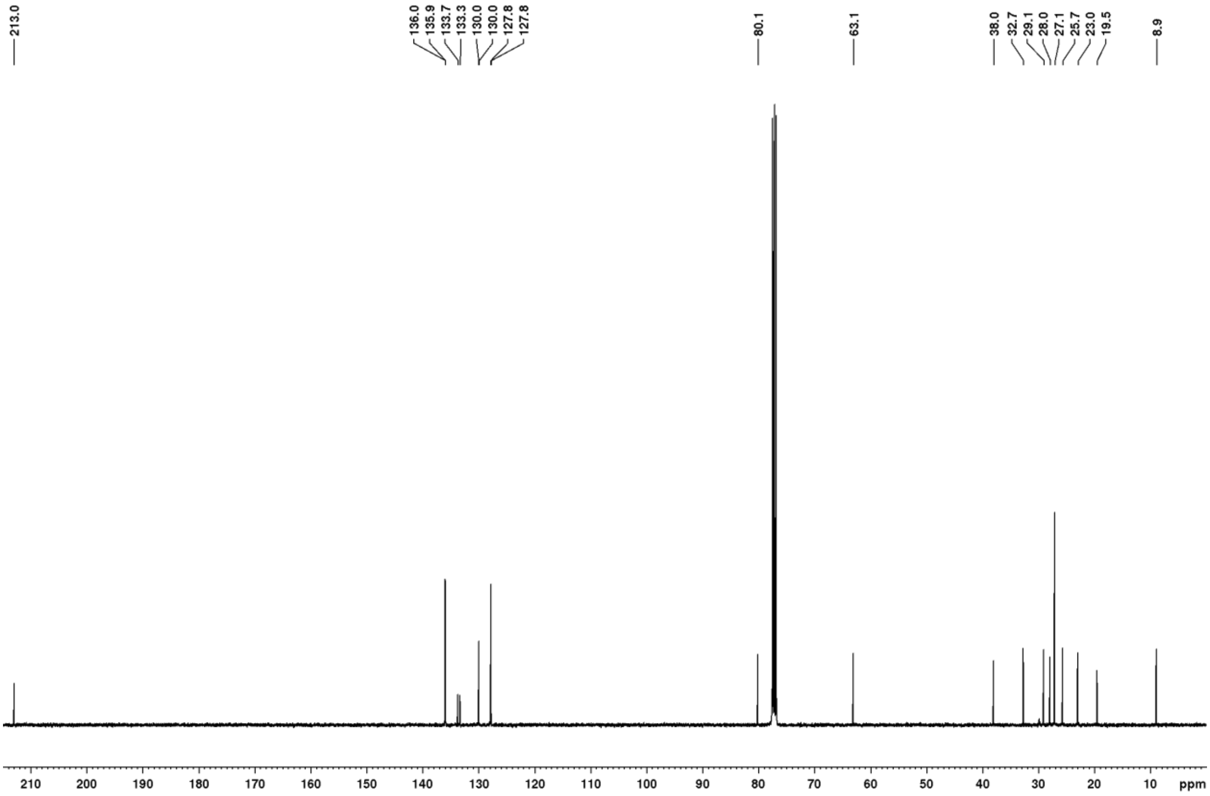

Figure 22: ^{13}C-NMR spectrum (100 MHz, $CDCl_3$) of compound **12**.

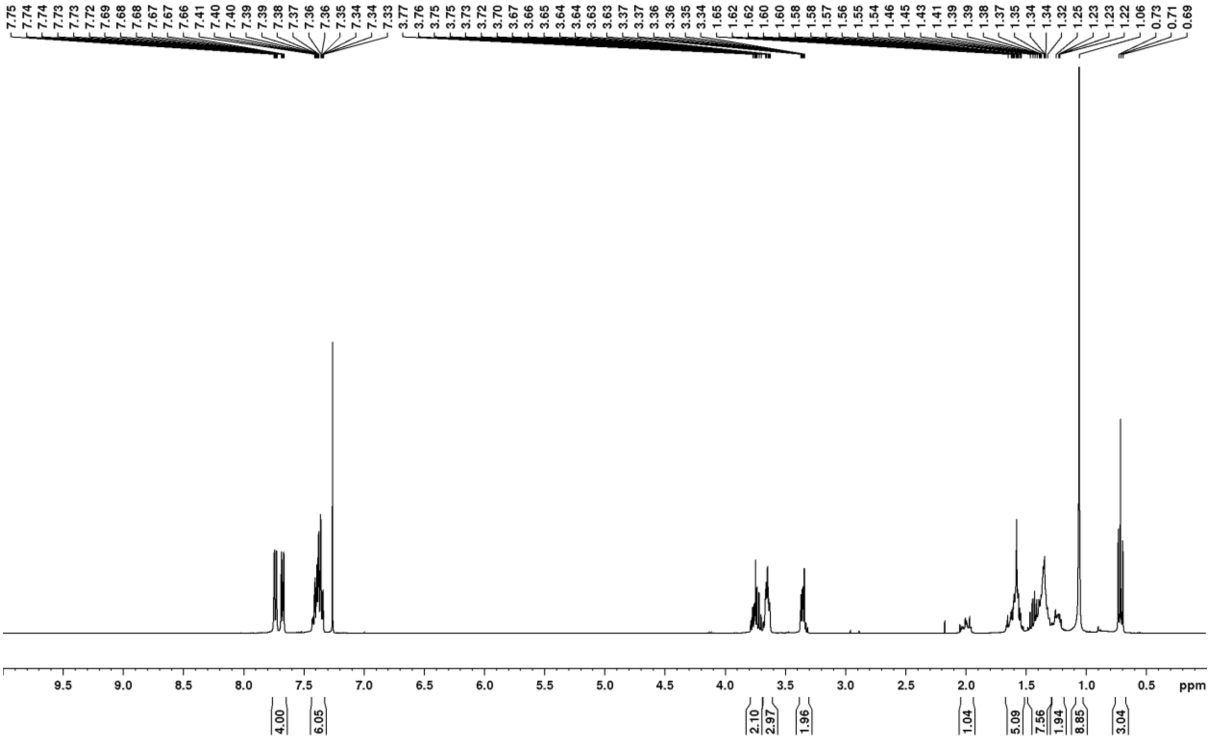

Figure 23: ^{1}H-NMR spectrum (400 MHz, $CDCl_3$) of compound **13**.

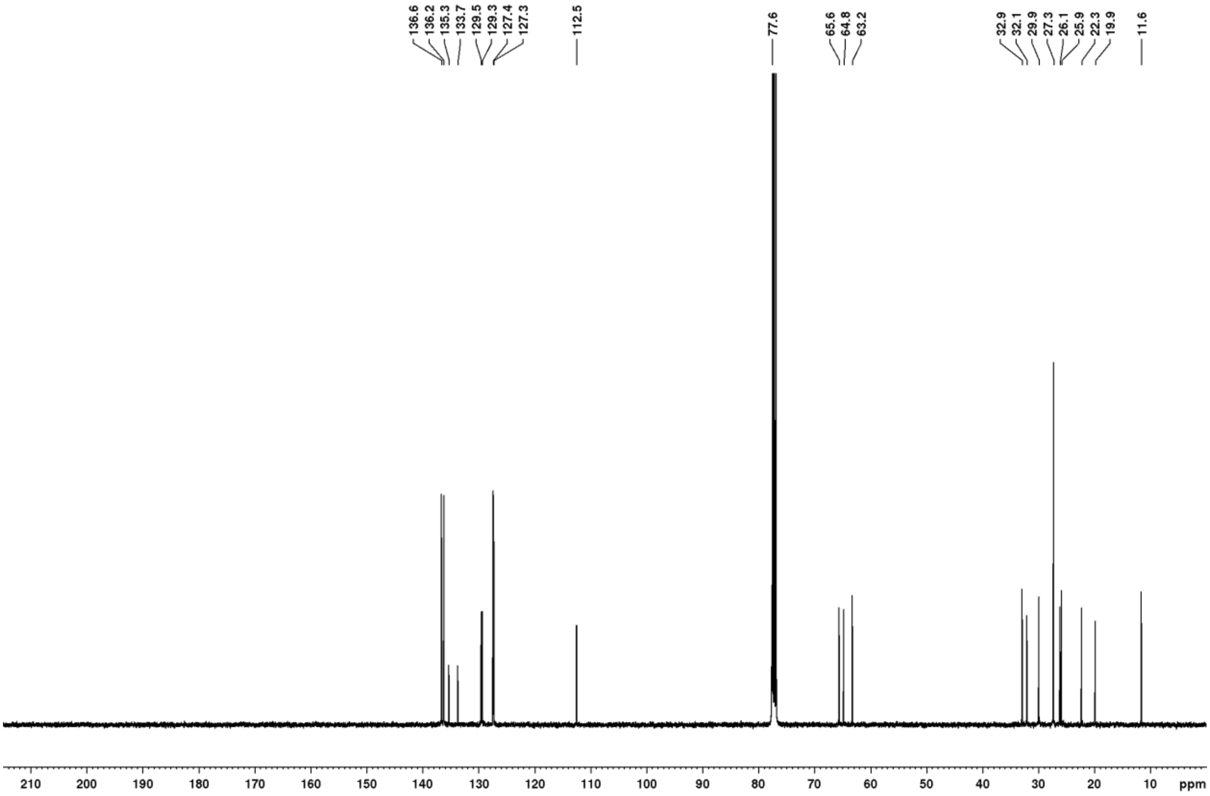

Figure 24: ^{13}C-NMR spectrum (100 MHz, $CDCl_3$) of compound **13**.

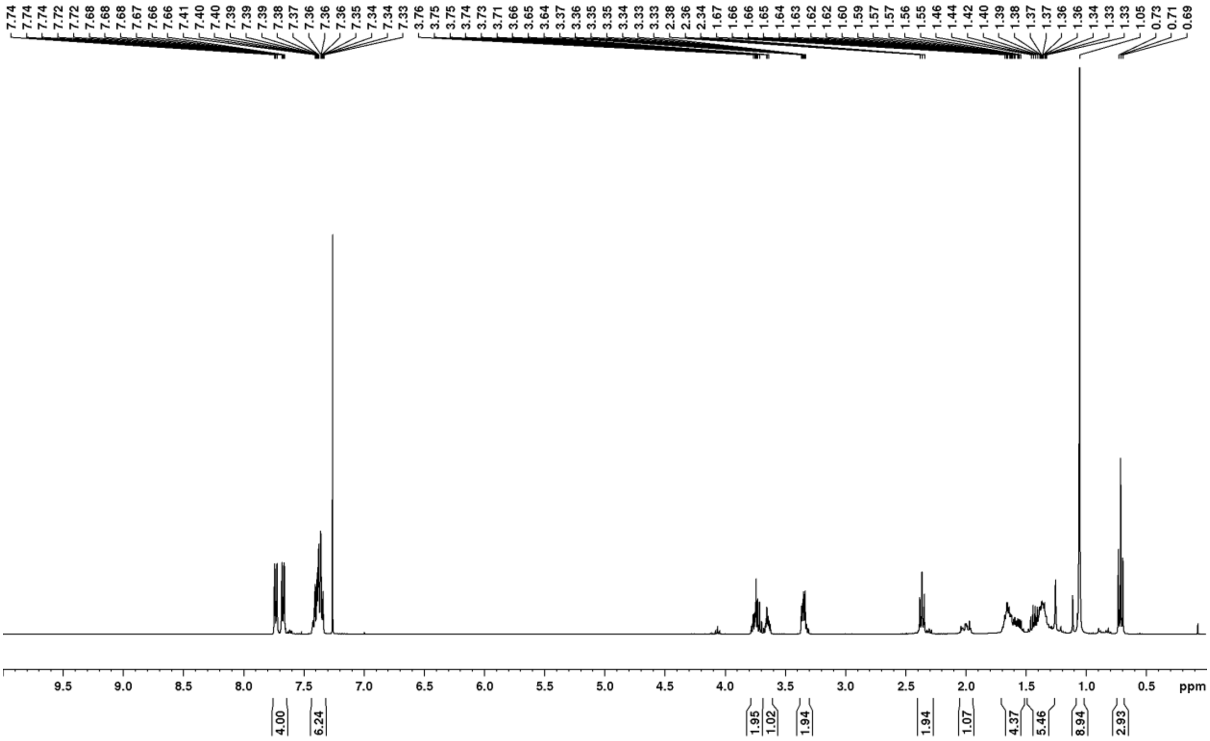

Figure 25: ^{1}H-NMR spectrum (400 MHz, $CDCl_3$) of compound **14**.

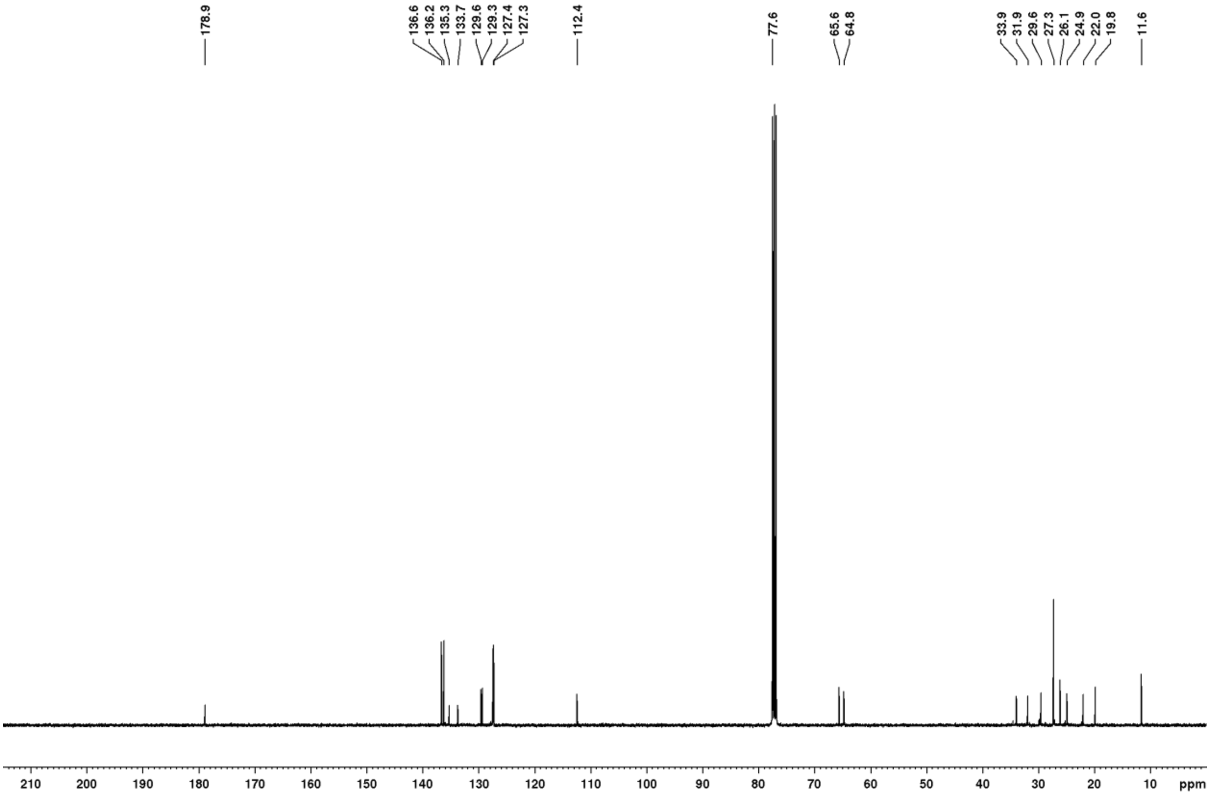

Figure 26: ^{13}C-NMR spectrum (100 MHz, $CDCl_3$) of compound **14**.

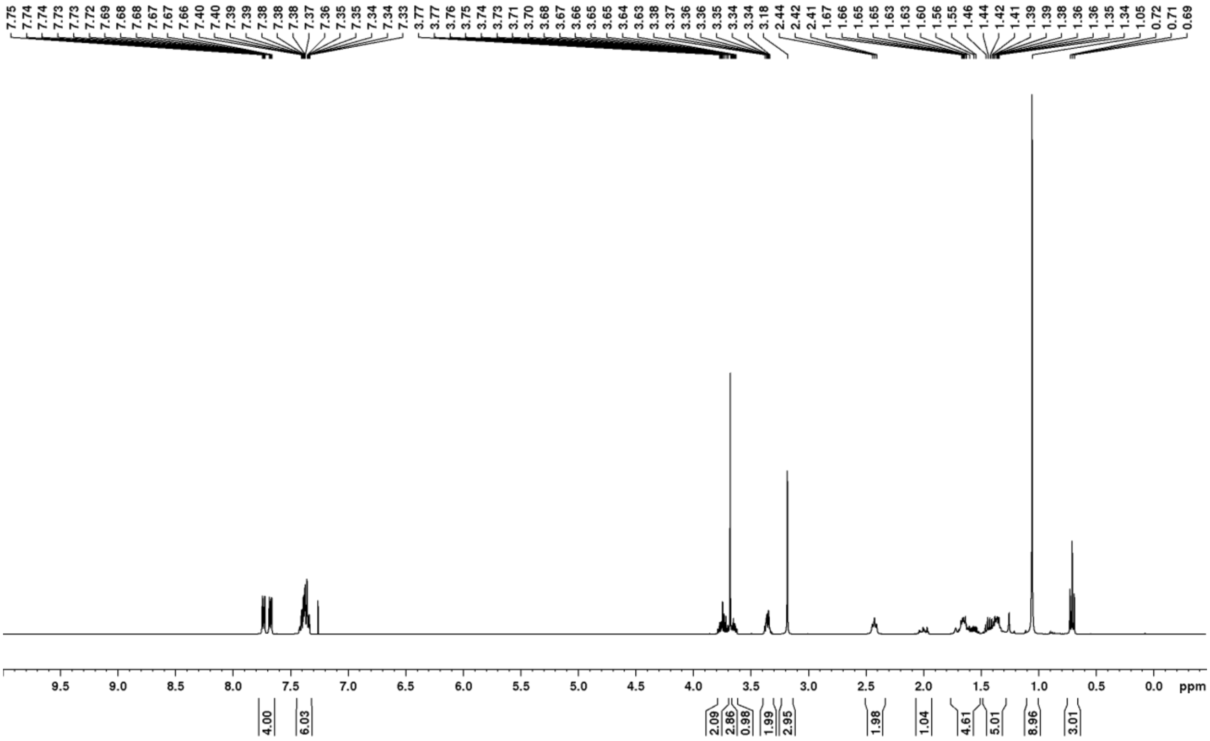

Figure 27: ^{1}H-NMR spectrum (400 MHz, $CDCl_3$) of compound **15**.

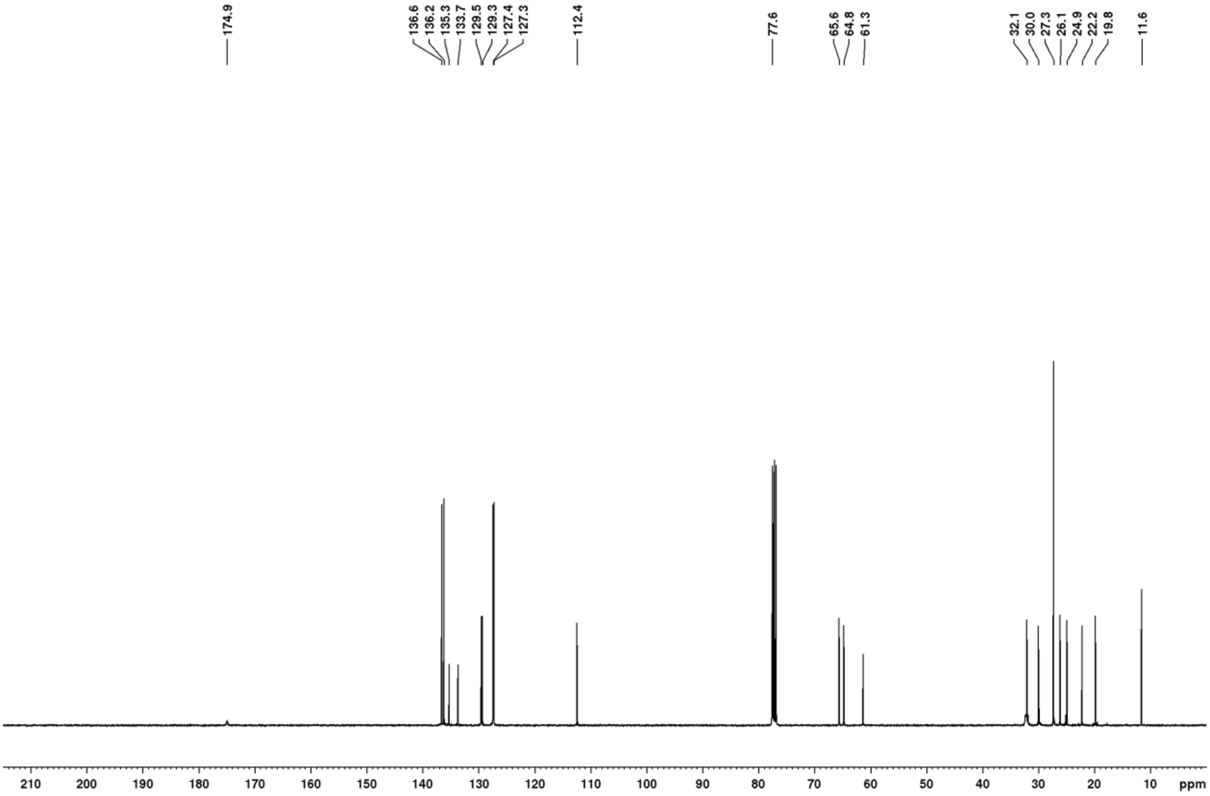

Figure 28: ^{13}C-NMR spectrum (100 MHz, $CDCl_3$) of compound **15**.

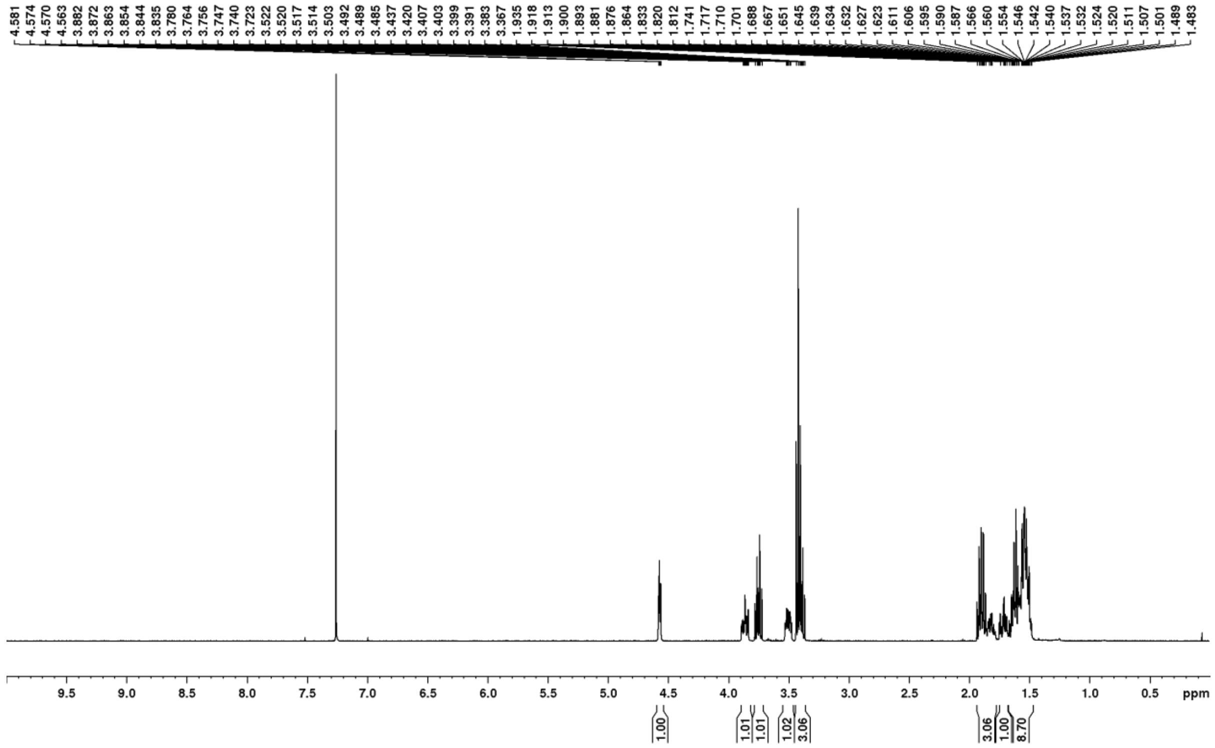

Figure 29: ^{1}H-NMR spectrum (400 MHz, $CDCl_3$) of compound **16**.

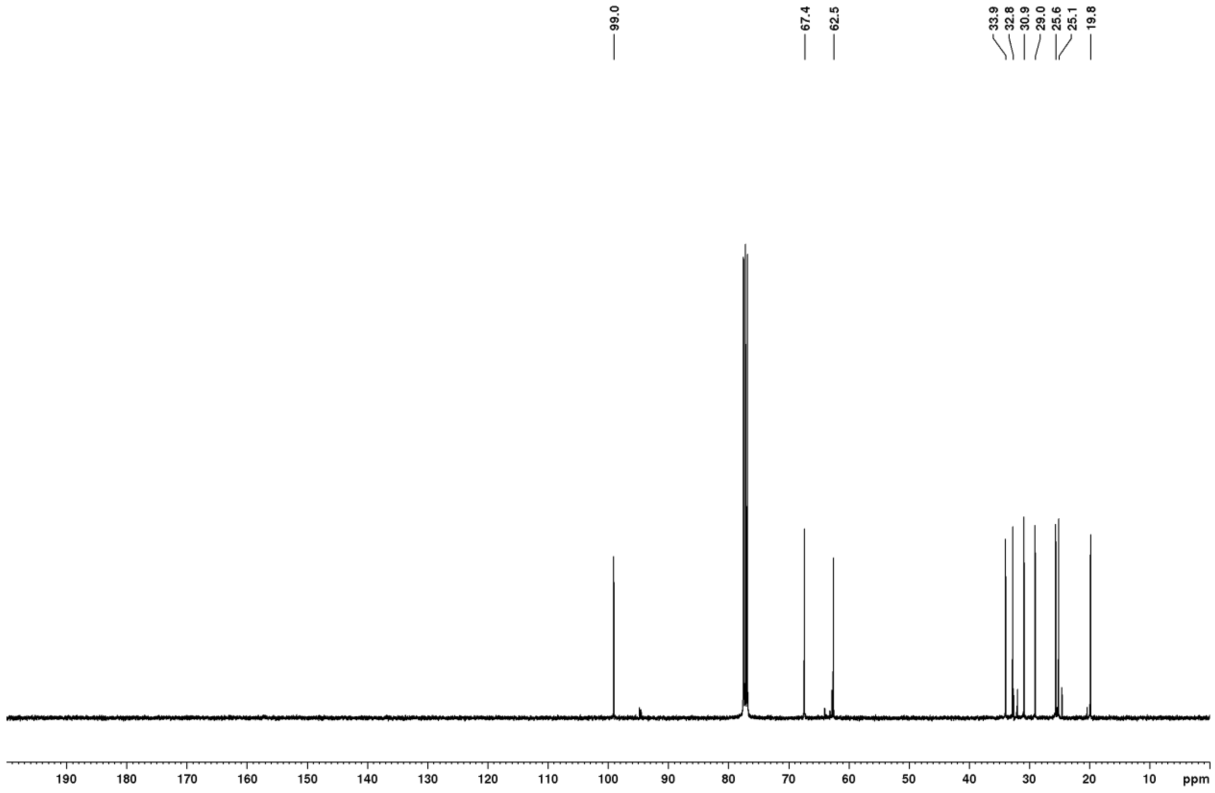

Figure 30: ^{13}C-NMR spectrum (100 MHz, $CDCl_3$) of compound **16**.

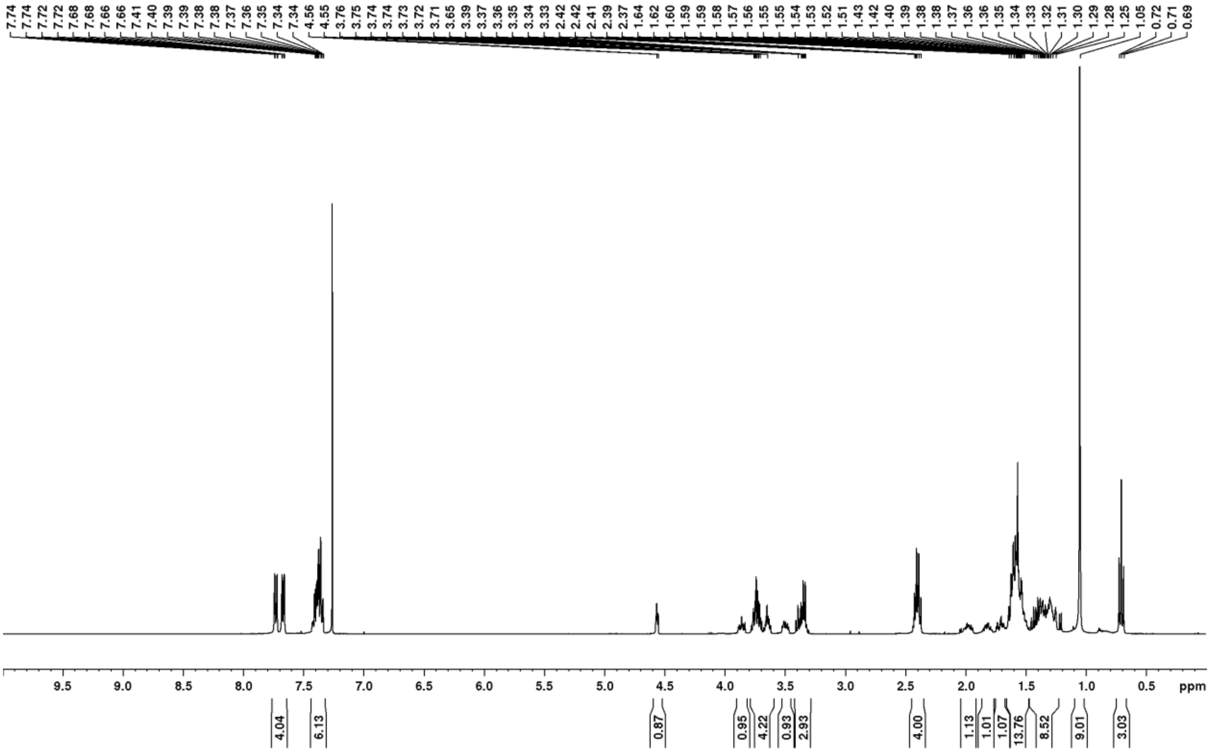

Figure 31: ^{1}H-NMR spectrum (400 MHz, $CDCl_3$) of compound **17**.

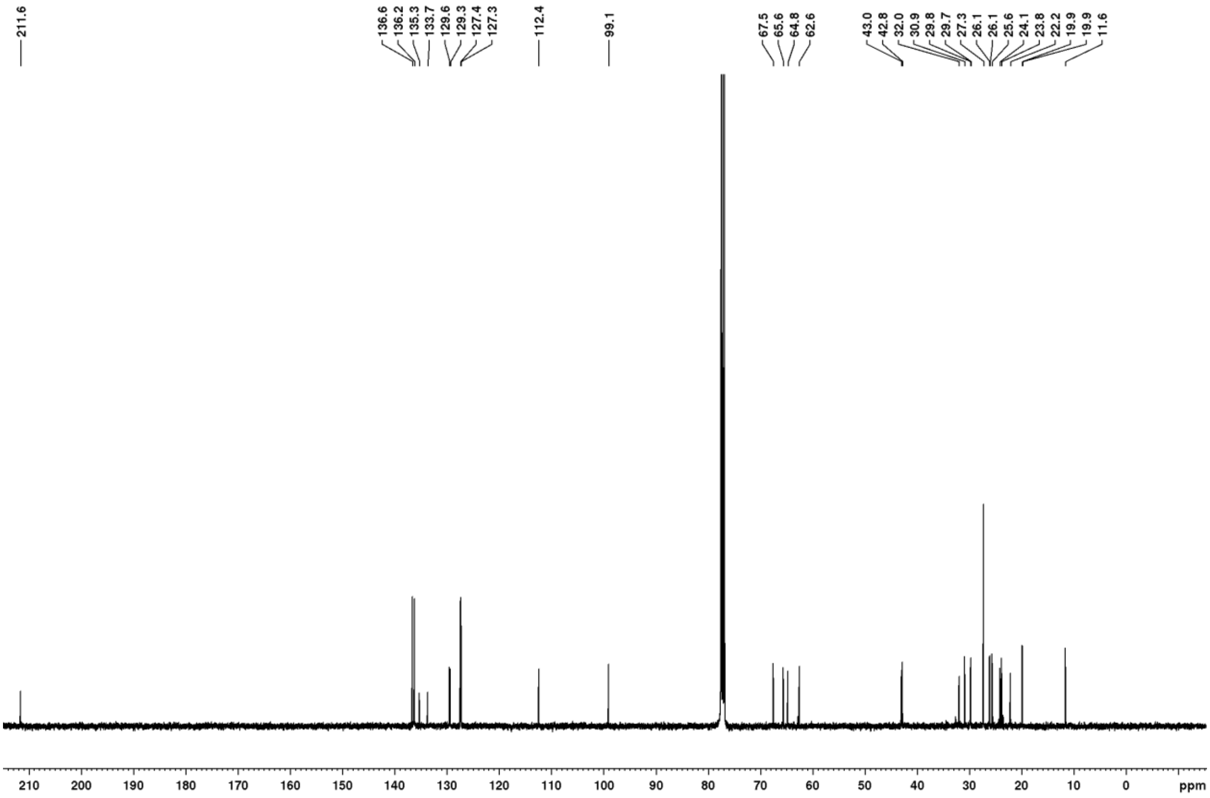

Figure 32: ^{13}C-NMR spectrum (100 MHz, $CDCl_3$) of compound **17**.

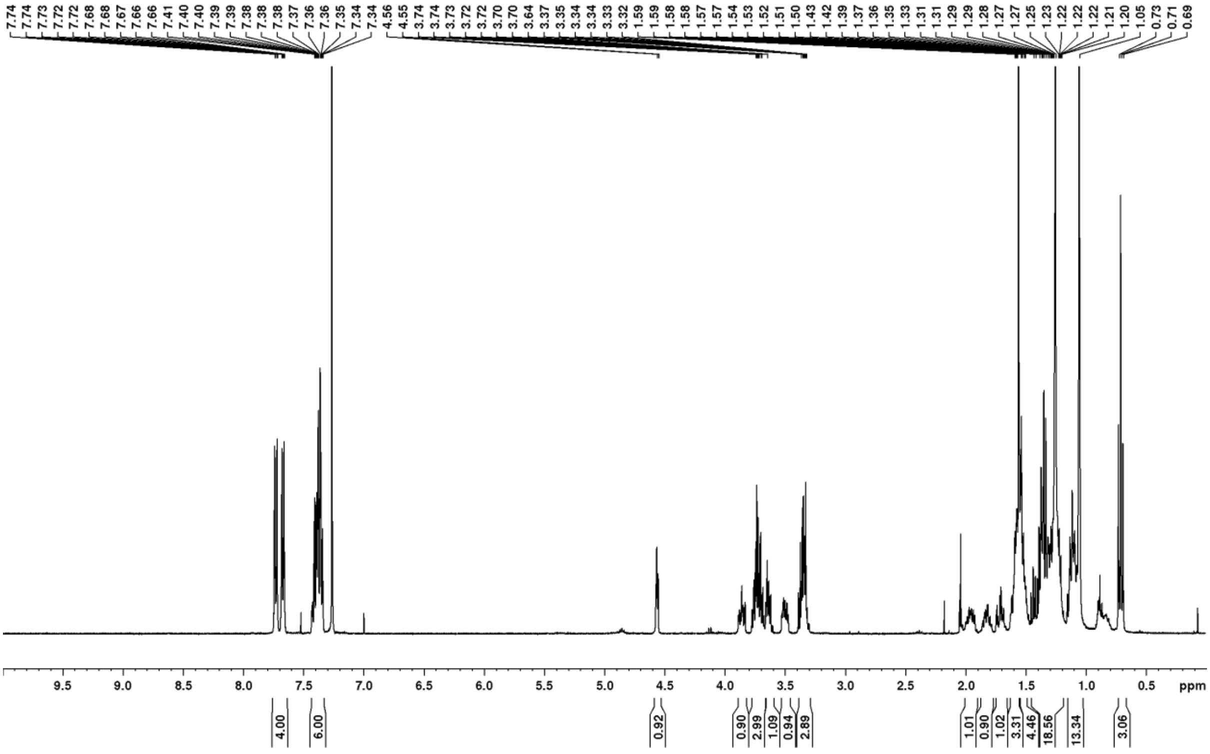

Figure 33: ^{1}H-NMR spectrum (400 MHz, $CDCl_3$) of compound **18**.

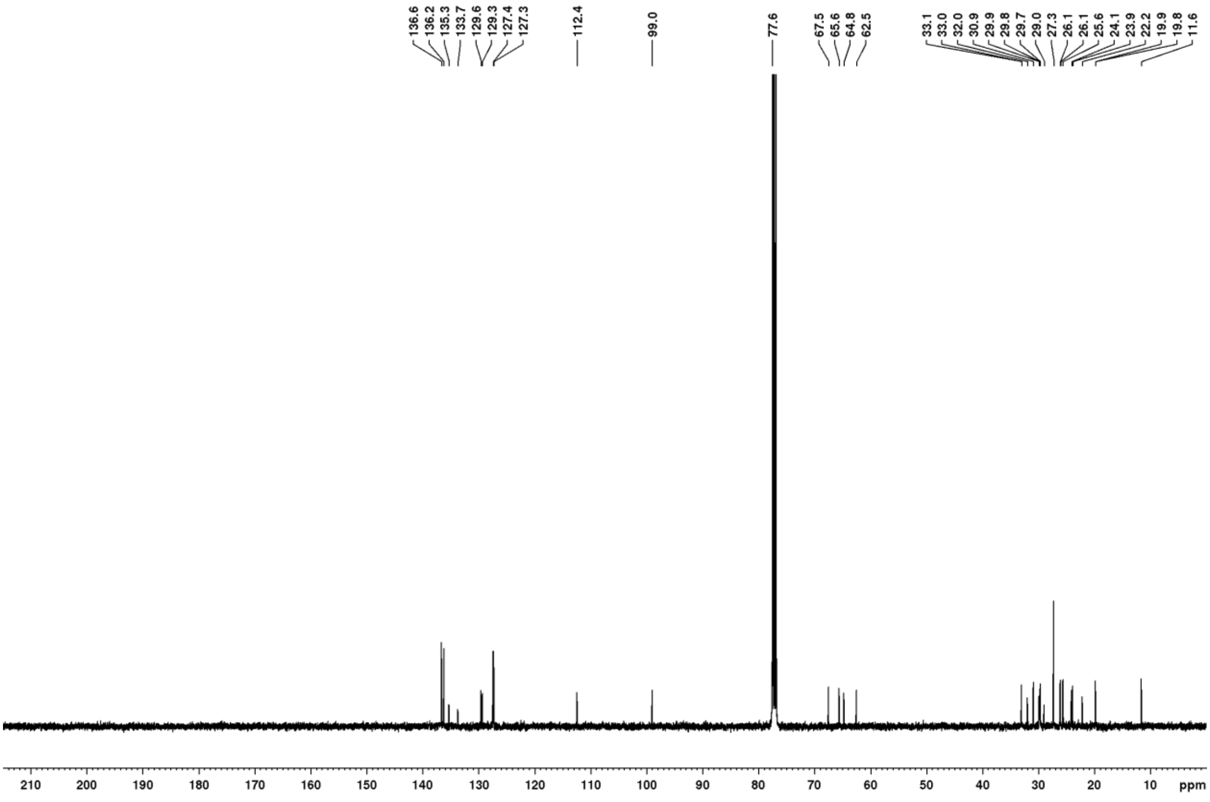

Figure 34: ^{13}C-NMR spectrum (100 MHz, $CDCl_3$) of compound **18**.

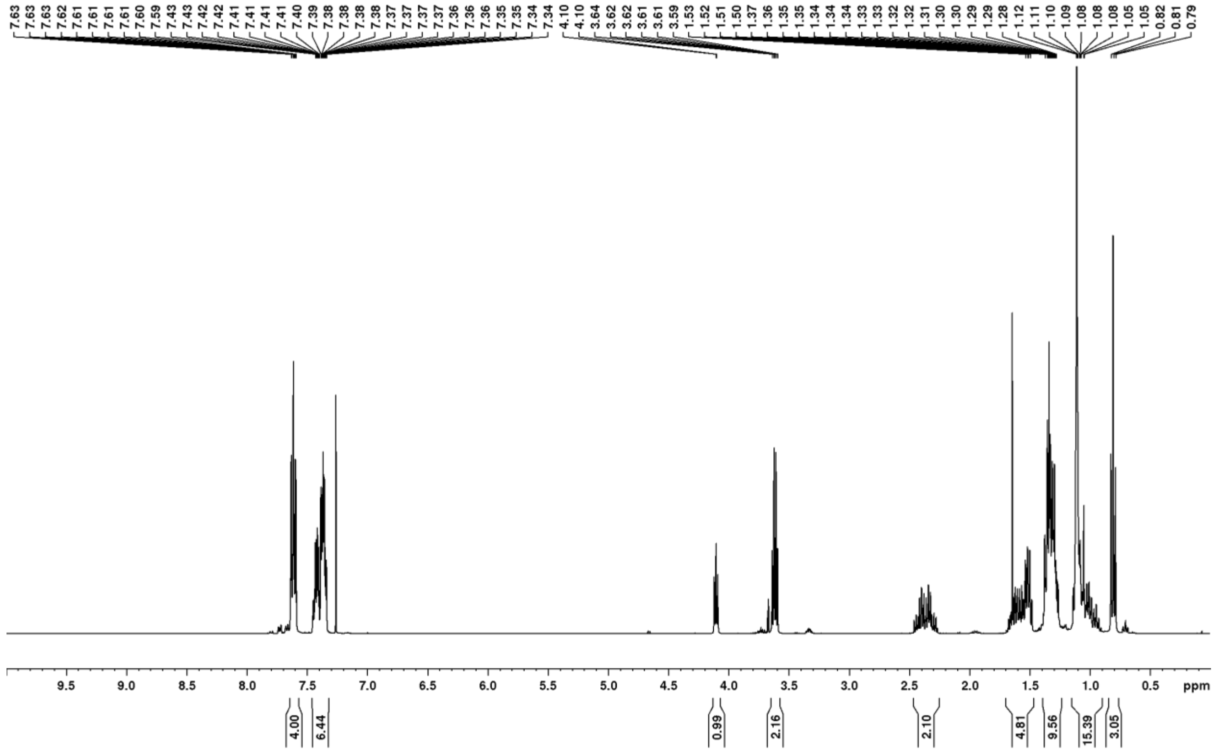

Figure 35: ^{1}H-NMR spectrum (400 MHz, $CDCl_3$) of compound **19**.

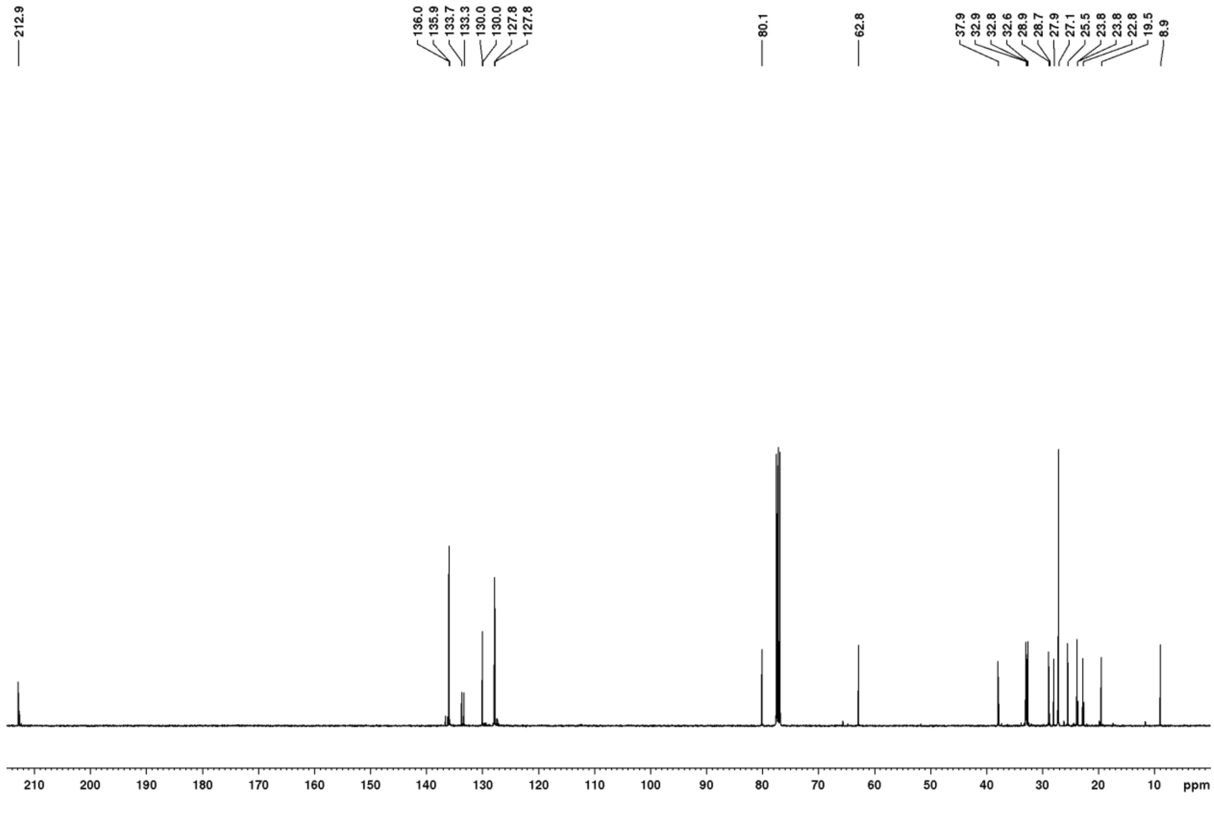

Figure 36: ^{13}C-NMR spectrum (100 MHz, $CDCl_3$) of compound **19**.

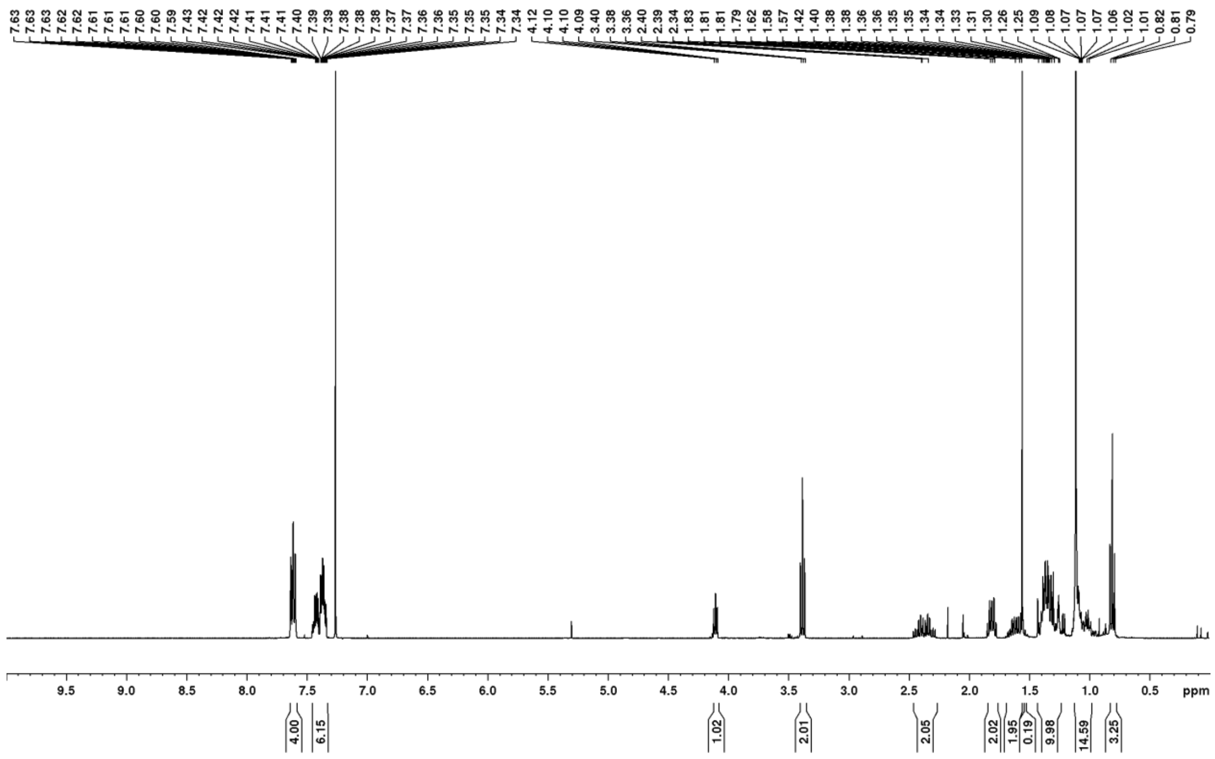

Figure 37: ^{1}H-NMR spectrum (400 MHz, $CDCl_3$) of compound **20**.

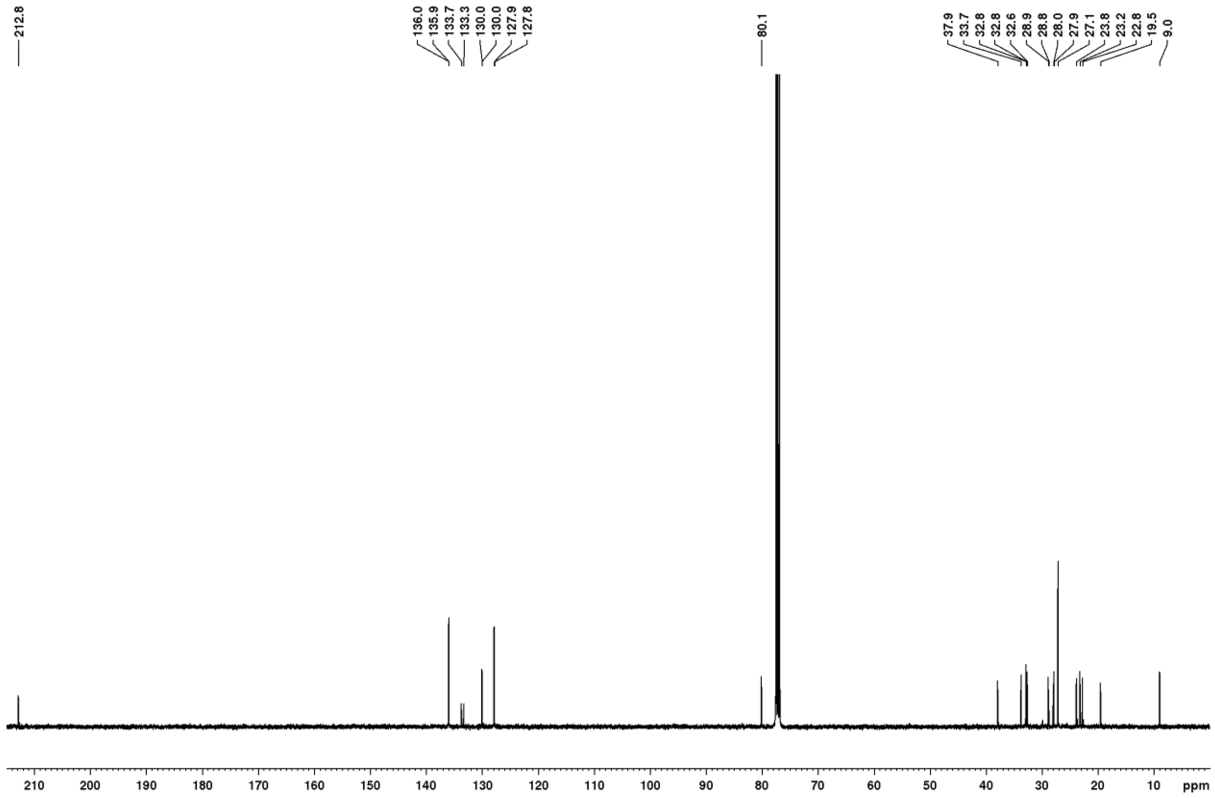

Figure 38: ^{13}C-NMR spectrum (100 MHz, $CDCl_3$) of compound **20**.

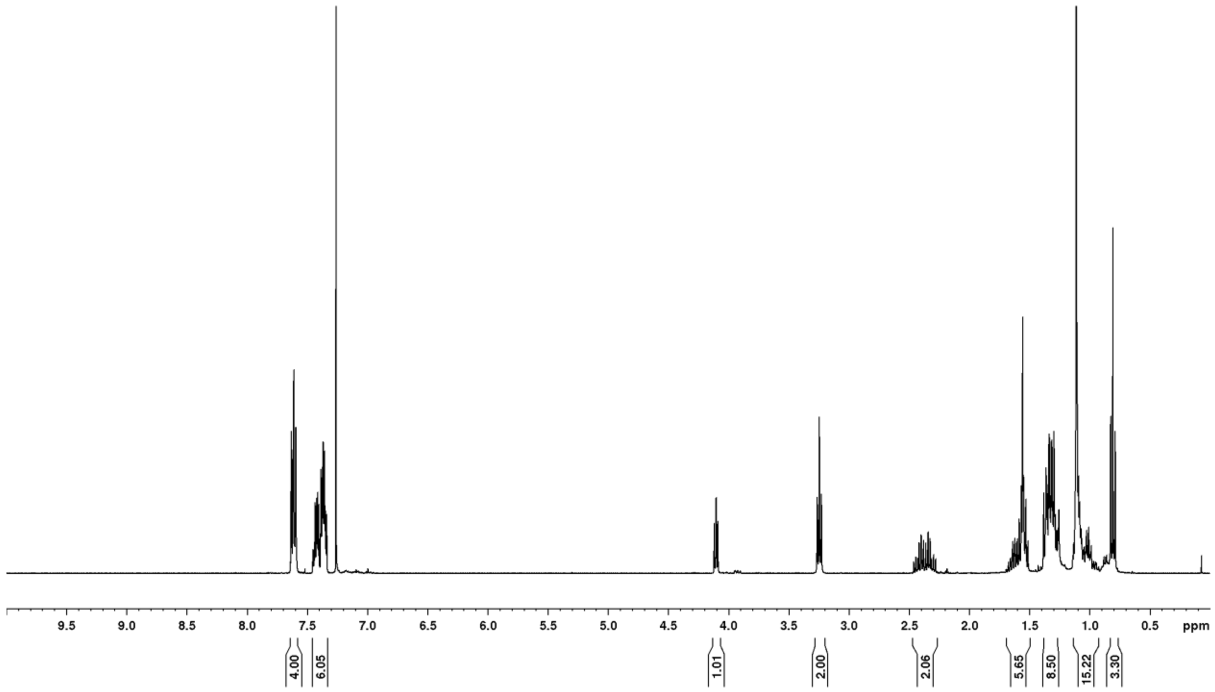

Figure 39: ^{1}H-NMR spectrum (400 MHz, $CDCl_3$) of compound **21**.

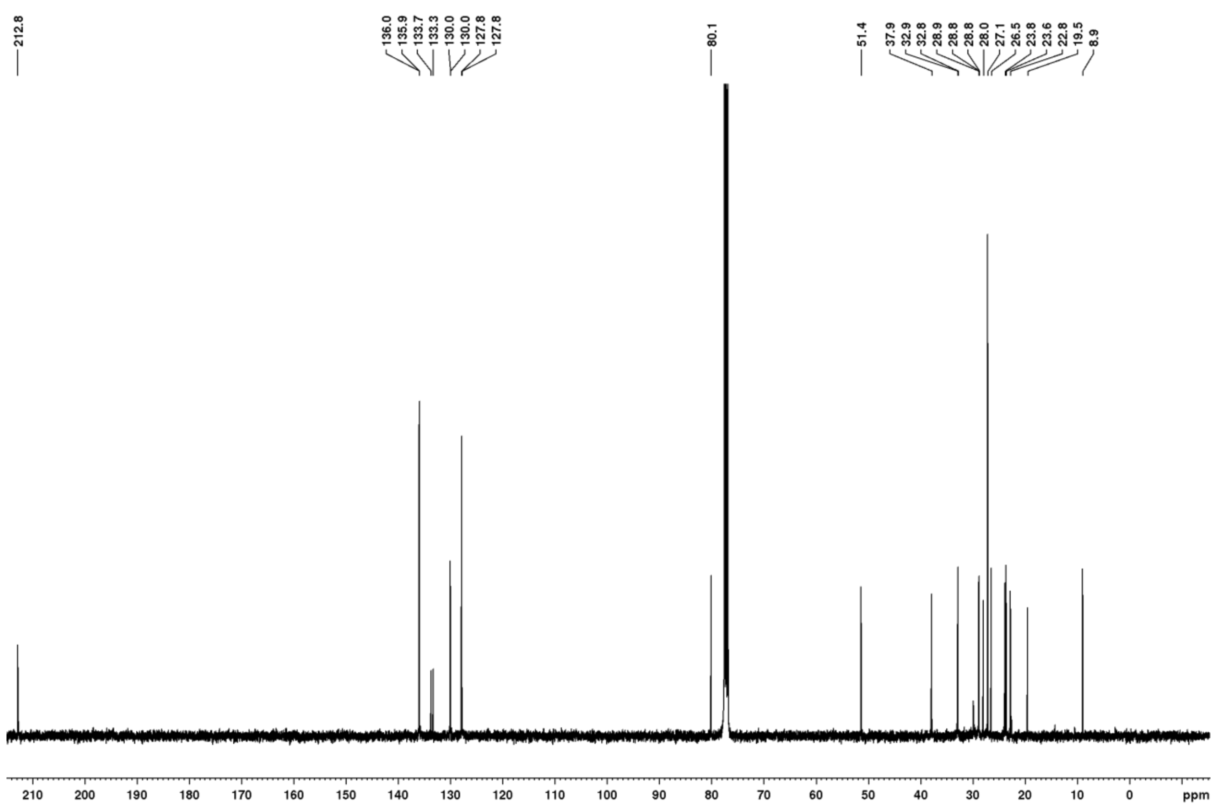

Figure 40: ^{13}C-NMR spectrum (100 MHz, $CDCl_3$) of compound **21**.

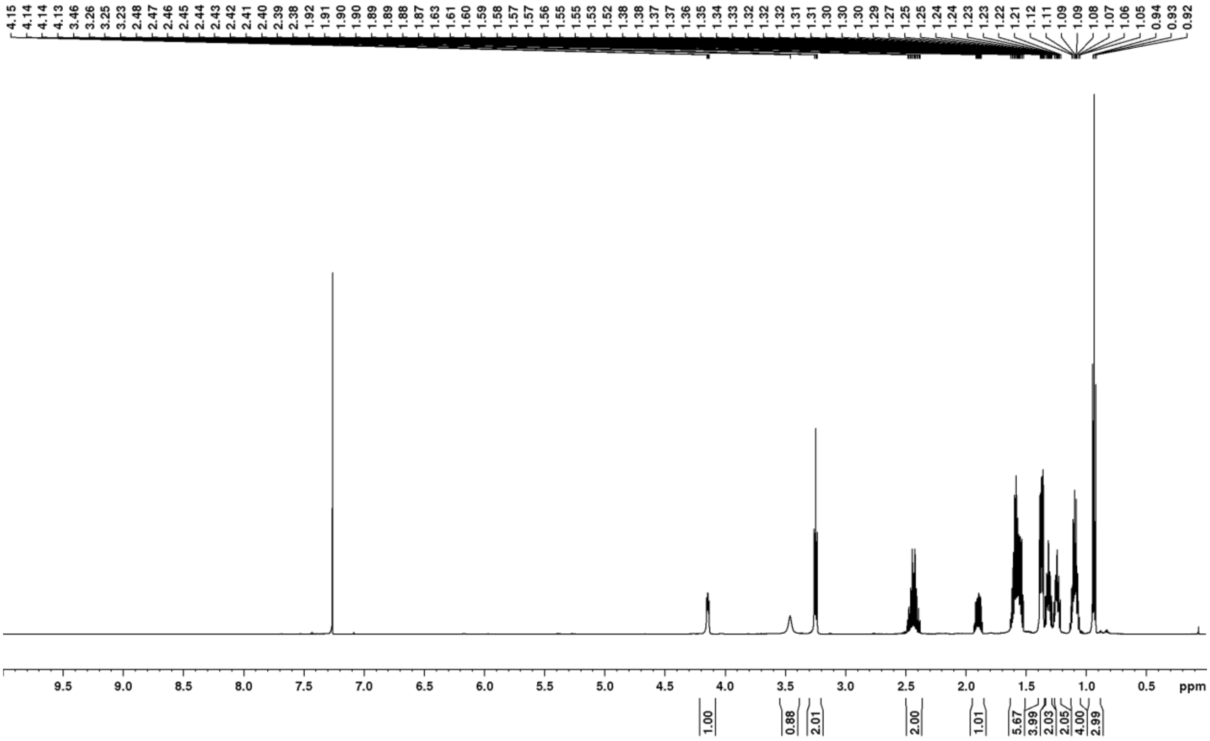

Figure 41: ^{1}H-NMR spectrum (600 MHz, $CDCl_3$) of compound **22**.

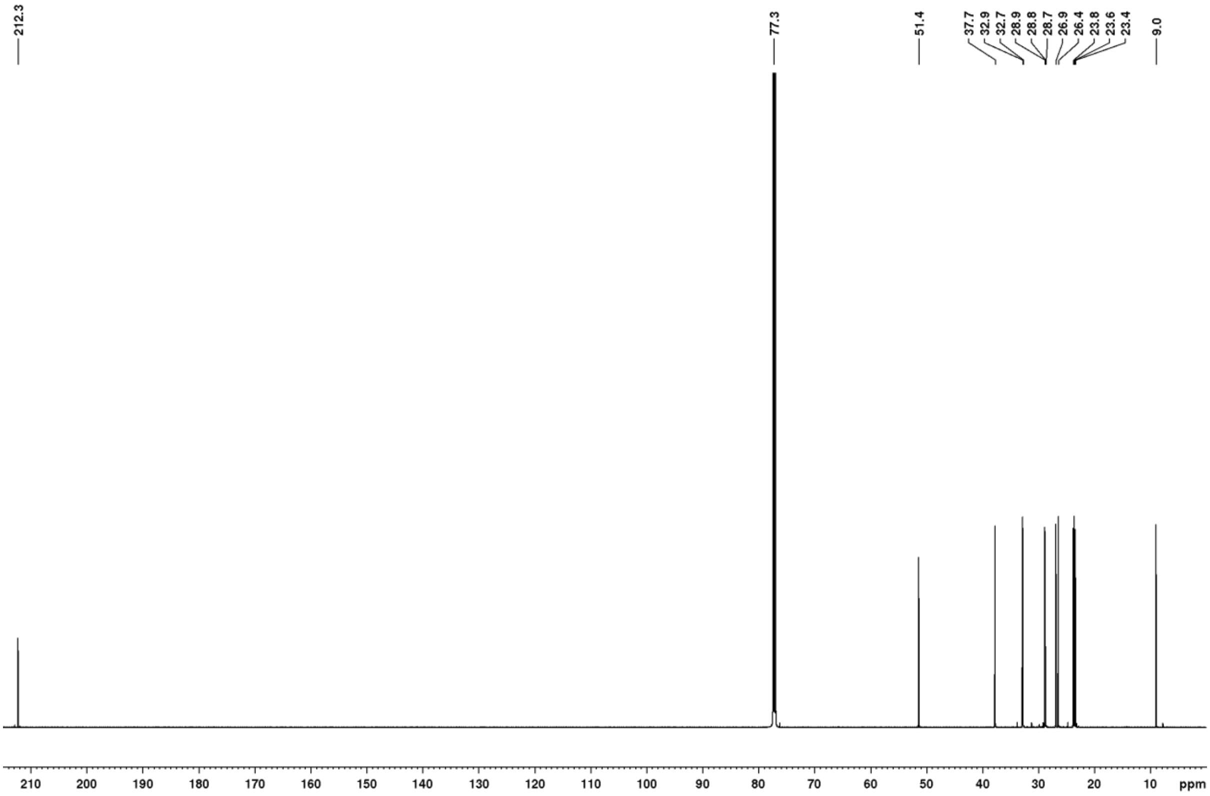

Figure 42: ^{13}C-NMR spectrum (150 MHz, $CDCl_3$) of compound **22**.
